# Long Intergenic Noncoding RNAs Mediate the Human Chondrocyte Inflammatory Response and Are Differentially Expressed in Osteoarthritis Cartilage

**DOI:** 10.1002/art.39520

**Published:** 2016-03-28

**Authors:** Mark J. Pearson, Ashleigh M. Philp, James A. Heward, Benoit T. Roux, David A. Walsh, Edward T. Davis, Mark A. Lindsay, Simon W. Jones

**Affiliations:** ^1^ University of BirminghamBirminghamUK; ^2^ University of BathBathUK; ^3^ University of NottinghamNottinghamUK; ^4^ The Royal Orthopaedic HospitalBirminghamUK

## Abstract

**Objective:**

To identify long noncoding RNAs (lncRNAs), including long intergenic noncoding RNAs (lincRNAs), antisense RNAs, and pseudogenes, associated with the inflammatory response in human primary osteoarthritis (OA) chondrocytes and to explore their expression and function in OA.

**Methods:**

OA cartilage was obtained from patients with hip or knee OA following joint replacement surgery. Non‐OA cartilage was obtained from postmortem donors and patients with fracture of the neck of the femur. Primary OA chondrocytes were isolated by collagenase digestion. LncRNA expression analysis was performed by RNA sequencing (RNAseq) and quantitative reverse transcriptase–polymerase chain reaction. Modulation of lncRNA chondrocyte expression was achieved using LNA longRNA GapmeRs (Exiqon). Cytokine production was measured with Luminex.

**Results:**

RNAseq identified 983 lncRNAs in primary human hip OA chondrocytes, 183 of which had not previously been identified. Following interleukin‐1β (IL‐1β) stimulation, we identified 125 lincRNAs that were differentially expressed. The lincRNA p50‐associated cyclooxygenase 2–extragenic RNA (PACER) and 2 novel chondrocyte inflammation–associated lincRNAs (CILinc01 and CILinc02) were differentially expressed in both knee and hip OA cartilage compared to non‐OA cartilage. In primary OA chondrocytes, these lincRNAs were rapidly and transiently induced in response to multiple proinflammatory cytokines. Knockdown of CILinc01 and CILinc02 expression in human chondrocytes significantly enhanced the IL‐1–stimulated secretion of proinflammatory cytokines.

**Conclusion:**

The inflammatory response in human OA chondrocytes is associated with widespread changes in the profile of lncRNAs, including PACER, CILinc01, and CILinc02. Differential expression of CILinc01 and CIinc02 in hip and knee OA cartilage, and their role in modulating cytokine production during the chondrocyte inflammatory response, suggest that they may play an important role in mediating inflammation‐driven cartilage degeneration in OA.

Osteoarthritis (OA), typified by degenerative loss of cartilage integrity and joint space narrowing, is a leading cause of pain, disability, and shortening of adult working life throughout the world 1, 2, 3. Unfortunately, at present there is no approved treatment that can modify the disease progression, resulting in limited therapeutic options for patients 4.

In attempting to identify novel therapeutics, inflammation is increasingly being recognized as an important driver of OA cartilage pathology. Histologic analysis, ultrasound, and magnetic resonance imaging have all demonstrated evidence of synovitis in OA joints 5, 6, 7, with increased cellular infiltration of activated B cells and T lymphocytes. Indeed, synovitis is reported not only in established OA, but also at the onset of OA, being present in patients with only minimal radiographic signs of the disease 8. Several proinflammatory cytokines are elevated in the synovial fluid of OA joints compared to normal healthy joints 9, and cytokine stimulation of ex vivo cartilage tissue mimics the pathologic changes observed within the OA joint 9, 10. However, the key regulators of the cellular inflammatory response in cartilage tissue are not well defined.

There is now overwhelming evidence that the microRNA (miRNA) family of short noncoding RNAs can regulate the inflammatory response 11, 12. Indeed, our group previously identified differentially expressed miRNAs in human OA cartilage tissue that mediated the production of matrix metalloproteinase 13 (MMP‐13) and tumor necrosis factor (TNF) 13, suggesting a role of miRNAs in regulating inflammation and OA pathology 13. Importantly, RNA sequencing (RNAseq) has now identified multiple families of long noncoding RNAs (lncRNAs), which include antisense RNAs, pseudogenes, and long intergenic noncoding RNAs (lincRNAs) 14, 15. Of interest, earlier reports suggest that these lncRNAs may also be central regulators of biologic processes 16, 17, 18, 19, including the inflammatory response 20. In support of those findings, we recently identified lncRNAs that were differentially expressed upon lipopolysaccharide (LPS)–induced activation of the human innate response and demonstrated that these regulated interleukin‐1β (IL‐1β) and IL‐8 production 21.

Currently, little is known about the expression and functional role of lncRNAs in OA joint tissue. Their potential importance is indicated in a recent report by Fu et al 22, who identified ∼4,700 lncRNAs that were differentially expressed in cartilage from patients with knee OA (compared with controls) using a microarray‐based approach. Although that preliminary study did not examine the function of these lncRNAs, another recent study has identified a lincRNA located upstream of the gene PTGS2 (cyclooxygenase 2 [COX‐2]). This was shown to be increased in phorbol myristate acetate– and LPS‐stimulated monocytes and to positively regulate COX‐2 expression 23 by binding to, and relieving the action of, the repressive p50 component of the NF‐κB complex 23. As a result of this action, the lincRNA was renamed p50‐associated COX‐2–extragenic RNA (PACER). Importantly, COX‐2 is a key regulator of the arachidonic acid pathway and subsequent prostaglandin E_2_ production 24, which is a putative mediator of inflammation and pain in OA cartilage tissue 25, 26. Given these observations, and the key role of inflammation in OA cartilage pathology, we hypothesized that lncRNAs, including PACER, are central regulators of the inflammatory response in cartilage tissue.

The aim of this study was therefore to perform RNAseq in order to identify lncRNAs that are associated with the inflammatory response in primary human OA chondrocytes isolated from the articular cartilage of patients with hip OA. We then proceeded to assess their potential involvement in OA by examining the expression of several “inflammation‐associated” lncRNAs (including PACER) in human articular cartilage from patients with and those without hip or knee OA, profiling their expression in response to multiple proinflammatory cytokines and determining the functional effect of modulating the expression of an inflammation‐associated lncRNA on the chondrocyte inflammatory response.

## PATIENTS AND METHODS

### Patients and tissue samples

Following ethics approval (UK National Research Ethics Committee 14/ES/1044), patients with hip OA (mean ± SEM age 69 ± 3 years; n = 9), patients with knee OA (age 70 ± 3 years; n = 12), and patients with fracture of the neck of the femur without OA (age 74 ± 2 years; n = 6) were recruited prior to elective joint replacement surgery at either The Royal Orthopaedic Hospital (Birmingham, UK) or Russell's Hall Hospital (Dudley, UK). Patients with hip OA had Kellgren/Lawrence (K/L) grades 27 of 3 or 4, patients with knee OA all had K/L grades of 4, and patients with fracture of the neck of the femur had K/L grades of 0. Cartilage from femoral condyles (from knee OA patients) and femoral heads (from hip OA patients) was collected. Ethics approval was also obtained (Derby Research Ethics Committee 1 [11/H0405/2]) to collect non‐OA knee cartilage from postmortem donors (mean ± SEM age 74 ± 5 years; n = 4) (Kings Mill Hospital, Sutton‐in‐Ashfield, UK) with no history of joint pain or evidence of cartilage fibrillation based on chondropathy assessment 28. Consent was obtained from all patients or families. Patient demographic data are provided in Supplementary Table 1, available on the *Arthritis & Rheumatology* web site at http://onlinelibrary.wiley.com/doi/10.1002/art.39520/abstract. A protocol was in place to ensure that samples were all handled in the same way and processed in the same timeframe. For tissue processing, upon separation of cartilage from bone tissue, the cartilage was immediately snap‐frozen in liquid nitrogen.

### Isolation of primary chondrocytes from articular cartilage

Articular cartilage was separated from the subchondral bone using a scalpel and digested using filter‐sterilized collagenase IIA (2 mg/ml; Sigma‐Aldrich) for 5 hours at 37°C. Digested cartilage was then filtered by passing through a 40‐μm cell strainer (BD Biosciences), and the filtrate was centrifuged. Primary chondrocytes were then resuspended in growth media (Dulbecco's modified Eagle's medium supplemented with 10% fetal calf serum [FCS], penicillin [100 units/ml], streptomycin [100 μg/ml], l‐glutamine [2 m*M*], nonessential amino acids [5% volume/volume] [all from Life Technologies], and amphotericin [2 μg/ml; Sigma‐Aldrich]) and grown to 70–80% confluence before being used in subsequent studies.

### RNAseq analysis

**Figure 1:**
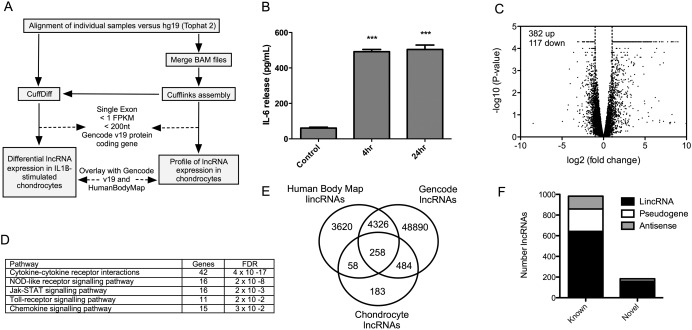
Regulation of long noncoding RNA (lncRNA) expression by interleukin‐1β (IL‐1β) in human osteoarthritis (OA) chondrocytes. **A,** Pipeline for predicting lncRNAs from Cufflinks‐assembled transfrags. FPKM = fragments per kilobase of transcript per million mapped reads. **B,** Release of IL‐6 from primary human hip OA chondrocytes left unstimulated or stimulated with IL‐1β for 4 hours or 24 hours, as measured by enzyme‐linked immunosorbent assay. IL‐6 release indicates activation of the inflammatory response. Bars show the mean ± SEM. ∗∗∗ = *P* < 0.001. **C,** Volcano plot displaying differentially expressed mRNAs (n = 3 IL‐1β–stimulated hip OA chondrocytes and 3 unstimulated hip OA chondrocytes.). **D,** Pathway analysis of differentially expressed mRNAs. FDR = false discovery rate. **E,** Overlap of lncRNAs in OA chondrocytes, Gencode version 19, and the HumanBodyMap catalogs. **F,** Breakdown of differentially expressed lncRNAs based on positional classifications.

Primary hip OA chondrocytes (n = 3 patients) were left unstimulated or stimulated with IL‐1β (1 ng/ml) for 4 hours in 0.1% FCS culture media in the absence of antibiotics and amphotericin. Total RNA was isolated using TRIzol reagent (Life Technologies), further purified (RNeasy column; Qiagen), and the RNA integrity number (RIN) was assessed (Agilent Bioanalyzer). All RIN values were >7, and 260:280 ratios (measured by NanoDrop) were >1.7. Ribosomal RNA was removed using Ribozero (Epicentre Technologies), and RNAseq (100‐bp paired‐end, stranded sequencing) was performed on an Illumina HiSeq 2000 sequencer. Subsequent analysis was undertaken using Tophat2/Cufflinks with alignment against the hg19 reference genome (Figure 1A). LncRNAs were identified using Cufflinks and then compared with known lncRNAs previously annotated in Gencode version 19 and the Human LincRNAs Catalog 29. CuffDiff was used to compare control and IL‐1β–treated cells to identify differentially expressed transcripts (false discovery rate [FDR] <0.05, fold change >2, and change in fragments per kilobase of transcript per million mapped reads [FPKM] >1). Sequence data are available through the GEO database under series number GSE74220.

### Analysis of lncRNA expression in primary chondrocytes and articular cartilage by quantitative reverse transcriptase–polymerase chain reaction (qRT‐PCR)

Articular hip and knee cartilage was snap‐frozen in liquid nitrogen and pulverized using a 6770 Freezer/mill (Spex Sample Prep). Total RNA was extracted from both powdered cartilage and primary chondrocytes using TRIzol and further purified using RNeasy columns. RIN values were >7, and 260:280 ratios were >1.7. Custom primers and FAM‐labeled probes were designed using Primer Express 3 software (Life Technologies) for qRT‐PCR. The qRT‐PCR was performed from 25 ng of total RNA in a one‐step reaction (QuantiFast One‐Step RT‐PCR kit; Qiagen) using a Roche LightCycler 480 II. The relative expression of lncRNAs was determined using the ΔΔC_t_ method, following normalization to 18S RNA. GAPDH expression (relative to 18S RNA) was comparable between non‐OA and OA cartilage in both hip and knee samples (see Supplementary Figure 1, available on the *Arthritis & Rheumatology* web site at http://onlinelibrary.wiley.com/doi/10.1002/art.39520/abstract).

### Inhibition of lincRNA expression in human chondrocytes using locked nucleic acid (LNA) GapmeRs

The human chondrocyte cell line TC28, which was previously characterized by Goldring et al 30, and provided to us as a gift from AstraZeneca, was transfected with either LNAs targeting CILinc01 or CILinc02 (30 n*M*) or with LNA control (30 n*M*) using Lipofectamine 2000 (Life Technologies). Following 24‐hour transfection, cells were stimulated (in 0.1% FCS culture media in the absence of antibiotics and amphotericin) for either 4 hours or 24 hours with IL‐1β (1 ng/ml). Supernatants were collected for subsequent cytokine analysis with Luminex. Cells were lysed with RLT (Qiagen) for subsequent RNA extraction to examine knockdown of CILinc01 expression.

### Analysis of cytokine production in human chondrocyte supernatants

Supernatants from human chondrocyte–transfected cells and media controls were assayed for the concentration of 17 human proinflammatory cytokines using a human cytokine 17‐plex immunoassay (Bio‐Plex Pro; Bio‐Rad). The interassay variability is <15%; intraassay variability is <10%. Cross‐reactivity is <1%, and the dynamic range is between 1 and 2,500 pg/ml. Briefly, nondiluted chondrocyte cell culture supernatants were incubated with a magnetic Bio‐Plex bead cocktail consisting of beads specific for IL‐1β, IL‐2, IL‐4, IL‐5, IL‐6, IL‐7, IL‐8, IL‐10, IL‐12 (p70), IL‐13, IL‐17, granulocyte colony‐stimulating factor (G‐CSF), granulocyte–macrophage colony‐stimulating factor, interferon‐γ, monocyte chemotactic protein 1, macrophage inflammatory protein 1β (MIP‐1β), and TNF. A Bio‐Plex Pro Wash Station was used to wash the beads between incubation steps using the wash buffer supplied with the kit. A biotinylated secondary antibody was added, and quantification was carried out using a streptavidin–phycoerythrin substrate with fluorescence detected on a Bio‐Plex 200 System (Bio‐Rad/Luminex).

### Statistical analysis

Data were analyzed using SPSS software. Analysis of variance was performed throughout, followed by Fisher's least significant difference post hoc test, where appropriate. In all cases, data are presented as the mean ± SEM, and *P* values less than 0.05 were considered significant.

## RESULTS

### RNAseq transcriptome profile of primary human OA chondrocytes in response to stimulation with IL‐1β

IL‐1β stimulation of primary human hip OA chondrocytes (n = 3 patients) induced a rapid release of IL‐6 protein that peaked at 4 hours and remained elevated at 24 hours (Figure 1B). IL‐1β stimulation also induced a significant increase in the release of MMP‐13 at 24 hours (see Supplementary Figure 2, available on the *Arthritis & Rheumatology* web site at http://onlinelibrary.wiley.com/doi/10.1002/art.39520/abstract). Analysis of RNAseq data for Gencode‐annotated messenger RNAs (mRNAs) showed that 499 protein‐coding genes were differentially expressed upon IL‐1β stimulation (382 up‐regulated and 117 down‐regulated) (Figure 1C and Supplementary Table 2, available on the *Arthritis & Rheumatology* web site at http://onlinelibrary.wiley.com/doi/10.1002/art.39520/abstract). As expected, the up‐regulated genes from this set were significantly enriched (FDR <0.05) in Kyoto Encyclopedia of Genes and Genomes pathways involved in the inflammatory response (Figure 1D). There were no significantly enriched pathways in down‐regulated genes. This initial evaluation therefore demonstrated rapid and widespread induction of inflammatory gene expression following IL‐1β stimulation of human chondrocytes.

### Identification of novel lncRNAs in chondrocytes by RNAseq

Using the computational analysis pathway described in Figure 1A, we identified 983 lncRNAs in human chondrocytes, which could be divided into 642 lincRNAs, 124 antisense RNAs, and 217 pseudogenes (see Supplementary Table 3, available on the *Arthritis & Rheumatology* web site at http://onlinelibrary.wiley.com/doi/10.1002/art.39520/abstract). Of these assembled genes, 158 lincRNAs and 25 antisense RNAs had not previously been identified in Gencode version 19 or HumanBodyMap lncRNA (Figures 1E and F). As previously reported 14, 15, the mean FPKM, length, and exon number for lncRNAs were smaller than those for mRNAs (mean FPKM 4.7 for lncRNAs and 29.6 for mRNAs, mean length 1.2 kb for lncRNAs and 2.8 kb for mRNAs, and mean exon number 3.6 for lncRNAs and 16.4 for mRNAs).

Based on sequencing in ∼400 human cell types including chondrocytes, the FANTOM project has recently released an atlas of 43,011 enhancer regions that are characterized by bidirectional transcription of single‐exon efference RNAs (eRNAs) 31. Interestingly, we found that <4% of our identified lncRNAs overlapped with putative eRNA regions (see Supplementary Table 3, available on the *Arthritis & Rheumatology* web site at http://onlinelibrary.wiley.com/doi/10.1002/art.39520/abstract). Furthermore, visual inspection and the fact that our transcripts were unidirectional and multiexonic indicated that these lncRNAs did not represent eRNAs.

### Induction of widespread changes in lncRNA expression by IL‐1β stimulation

**Figure 2:**
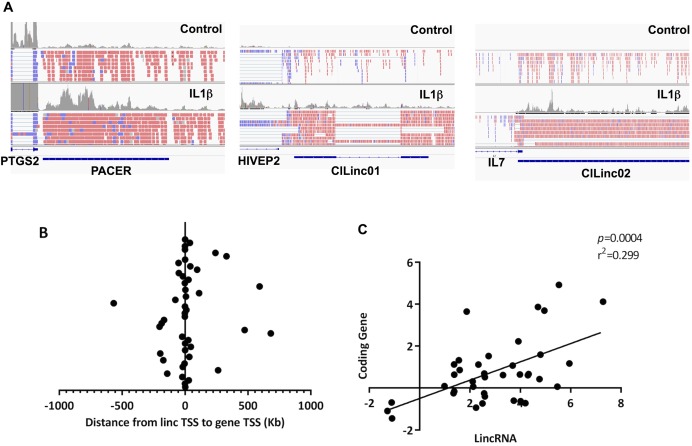
Location and expression of chondrocyte inflammation–associated long intergenic noncoding RNAs (lincRNAs) in relation to their nearest protein‐coding gene. **A,** Integrative Genomics Viewer plots showing the mapping data and relative locations of the long noncoding RNA (lncRNA) and protein‐coding genes in hip osteoarthritis (OA) chondrocyte samples left unstimulated (control) and samples stimulated with interleukin‐1β (IL‐1β) for 4 hours. Colors represent the direction of first read. Red blocks represent forward (positive) strand; blue blocks represent reverse (negative) strand; gray blocks represent reads of unknown status. PACER = p50‐associated cyclooxygenase 2–extragenic RNA. **B,** Dot plot showing the distances between transcription start sites (TSS) of novel lincRNAs and the TSS of their nearest protein‐coding gene. **C,** Pearson's correlation between absolute fold change in expression of lincRNAs and absolute fold change of their nearest expressed protein‐coding gene in primary human OA chondrocytes after stimulation with IL‐1β for 4 hours.

Following IL‐1β stimulation, we identified 125 lncRNAs that were differentially expressed (*P* < 0.05), including 93 lincRNAs (74%), 13 antisense RNAs (11%), and 19 pseudogenes (15%) (see Supplementary Table 4, available on the *Arthritis & Rheumatology* web site at http://onlinelibrary.wiley.com/doi/10.1002/art.39520/abstract). Of these, we observed 106 up‐regulated and 19 down‐regulated lncRNAs, of which 37 (30%) were novel lncRNAs. Using the Integrative Genomics Viewer (Broad Institute), the transcription start sites (TSS) for the majority of the 92 differentially expressed lincRNAs were found to be genomically located <5 kb from the TSS of a coding mRNA (Figures 2A and B). Previously, we have referred to these as mRNA‐flanking lincRNAs 21, and it has been suggested that they may regulate the expression of the nearby mRNA. In support of this notion, we found a significant positive correlation between the fold change in expression of an mRNA‐flanking lincRNA and the fold change in expression of its nearest coding mRNA (Figure 2C). In addition, detailed examination of these differentially expressed mRNA‐flanking lincRNAs identified one as being PACER (Table 1). As previously described, PACER is located upstream of the PTGS2 (COX‐2) gene, is transcribed in a bidirectional manner from the same promoter region, and is known to positively regulate PTGS2 expression 23 (Figure 2A). However, whether this is true of other mRNA‐flanking lincRNAs remains to be elucidated.

**Table 1:** Human chondrocyte inflammation–associated lincRNAs and their expression in human OA and non‐OA cartilage tissue^a^

LincRNA	LncRNA number	Position	Nearest gene (kb to TSS)	Fold change in expression after IL‐1β stimulation, log2
PTGS2‐lincRNA (PACER)	XLOC_081995	chr9:21682903‐21689760	PTGS2 (0.188)	3.1^b^
CILinc01	XLOC_043077	chr6:143267747‐143280112	HIVEP2 (1.409)	6.0^b^
CILinc02	XLOC_078832	chr8:79717154‐79798424	IL‐7 (0.604)	7.9^b^
CILinc03	XLOC_080615	chr8:90627962‐90765918	RIPK2 (4.056)	2.6^b^
CILinc04	XLOC_048072	chr21:43188194‐43194760	RIPK4 (0.928)	3.7^b^
CILinc05	XLOC_072067	chr6:138175998‐138186493	TNFAIP3 (1.857)	1.8^c^
CILinc06	XLOC_076579	chr7:80553659‐80558813	SEMA3C (7.138)	1.4^b^
CILinc07	XLOC_048423	chr21:28984539‐29019990	ADMATS5 (681.158)	5.0^b^

a LincRNA = long intergenic noncoding RNA; OA = osteoarthritis; lncRNA = long noncoding RNA; TSS = transcription start site; IL‐1β = interleukin‐1β; PACER = p50‐associated COX‐2–extragenic RNA.

b = *P* < 0.001 versus unstimulated control chondrocytes.

c = *P* < 0.01 versus unstimulated control chondrocytes.

### Differential expression of inflammation‐associated lincRNAs in human hip OA and knee OA cartilage

We next wished to further characterize the expression of PACER as well as 7 additional chondrocyte inflammation–associated lincRNAs (named CILinc01–CILinc07) that were selected based on being significantly induced in response to IL‐1β stimulation (Table 1) and their nearest coding mRNA being a gene with purported evidence of a role in either inflammation or OA pathology (e.g., IL‐7 and ADAMTS‐5, respectively).

**Figure 3:**
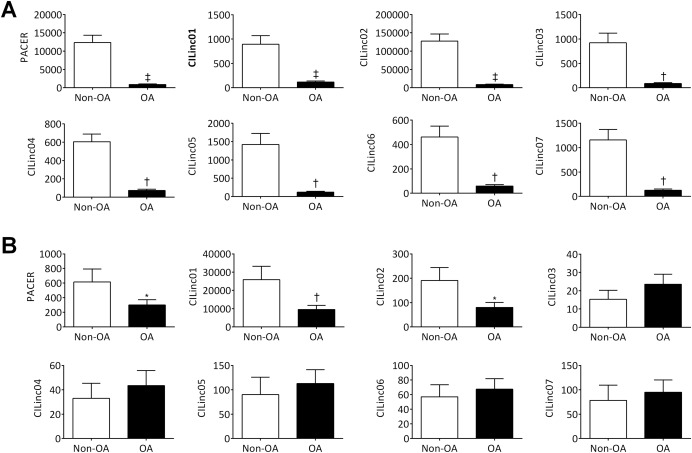
Expression of chondrocyte inflammation–associated long intergenic noncoding RNAs (lincRNAs) in human hip osteoarthritis (OA) and knee OA cartilage compared to non‐OA cartilage. Graphs show the relative expression of 8 chondrocyte inflammation–associated lincRNAs, as determined by quantitative reverse transcriptase–polymerase chain reaction, in **A,** OA hip femoral head articular cartilage (n = 9 patients) compared to non‐OA hip femoral head articular cartilage (n = 6 patients), and **B,** OA knee cartilage (n = 12 patients) compared to non‐OA knee cartilage (n = 4 patients). Bars show the mean ± SEM. ∗ = *P* < 0.05; † = *P* < 0.01; ‡ = *P* < 0.001, by one‐way analysis of variance. PACER = p50‐associated cyclooxygenase 2–extragenic RNA.

We initially determined the potential clinical relevance of these chondrocyte inflammation–associated lincRNAs by measuring their expression in human OA hip cartilage compared to non‐OA hip cartilage. All 8 lincRNAs were found to be significantly down‐regulated in OA hip cartilage (n = 9 patients) compared to non‐OA hip cartilage (n = 6 patients) (Figure 3A). The lincRNAs PACER, CILinc01, and CILinc02 were also significantly down‐regulated (>2‐fold) in OA knee cartilage (n = 12) compared to non‐OA knee cartilage (n = 4) (Figure 3B).

### Rapid, transient induction of lincRNAs by multiple proinflammatory cytokines

**Figure 4:**
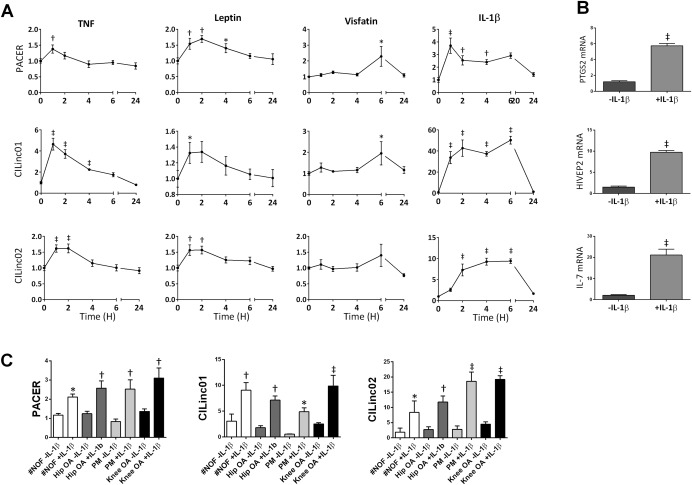
Rapid and transient induction of chondrocyte inflammation–associated long intergenic noncoding RNAs (lincRNAs) in primary human osteoarthritis (OA) chondrocytes in response to proinflammatory cytokines. **A,** Time course of lincRNA expression in primary human hip OA chondrocytes over 24 hours following exposure to either tumor necrosis factor (TNF; 1 ng/ml), leptin (100 ng/ml), visfatin (100 ng/ml), or interleukin‐1β (IL‐1β; 1 ng/ml). Symbols and error bars indicate the mean ± SEM. ∗ = *P* < 0.05; † = *P* < 0.01; ‡ = *P* < 0.001 versus time 0, by two‐way analysis of variance with a least significant difference post hoc test. **B,** Primary OA chondrocyte expression of PTGS2, HIVEP2, and IL‐7 genes in response to 4 hours of stimulation with IL‐1β (1 ng/ml). Bars show the mean ± SEM. ‡ = *P* < 0.001 versus unstimulated samples, by two‐way analysis of variance with a least significant difference post hoc test. **C,** Expression of p50‐associated cyclooxygenase 2–extragenic RNA (PACER), CILinc01, and CILinc02 in primary non‐OA chondrocytes isolated from cartilage from patients with fracture of the neck of the femur (#NOF) and from postmortem (PM) cartilage and in primary OA chondrocytes isolated from hip and knee cartilage. Expression of lincRNAs and genes was determined by quantitative reverse transcriptase–polymerase chain reaction and is shown as fold change compared to control. Bars show the mean ± SEM from 3 independent experiments. ∗ = *P* < 0.05; † = *P* < 0.01; ‡ = *P* < 0.001 versus unstimulated samples, by two‐way analysis of variance with a least significant difference post hoc test.

Based on their induction in response to IL‐1β stimulation, and their differential expression in both hip OA and knee OA cartilage, we next examined the time course of expression of PACER, CILinc01, and CILinc02 in primary OA chondrocytes in response to a panel of proinflammatory cytokines implicated in the pathogenesis of OA. Following stimulation with IL‐1β, TNF, visfatin, and leptin, we observed a rapid and time‐dependent induction of expression of all 3 lincRNAs (Figure 4A). Of note, stimulation with either TNF or leptin led to peak lincRNA expression at ∼2 hours, which had dropped toward baseline levels by 24 hours. Stimulation with IL‐1β or visfatin led to a slightly more prolonged induction of lincRNA expression, with peak induction of CILinc01 and CILinc02 between 4 and 6 hours (Figure 4A). Of note, stimulation with IL‐1β for 4 hours also led to significant (*P* < 0.001) induction of the expression of mRNA for the closest coding genes to PACER, CILinc01, and CILinc02, namely, PTGS2, HIVEP2, and IL‐7, respectively (Figure 4B).

We then assessed whether PACER, CILinc01, and CILinc02 were also present in non‐OA chondrocytes and whether stimulation of these cells with IL‐1β would also induce their expression. PACER, CILinc01, and CILinc02 were expressed in both non‐OA knee chondrocytes (isolated from postmortem cartilage) and non‐OA hip chondrocytes (isolated from patients with fracture of the neck of the femur). Furthermore, 4 hours of IL‐1β stimulation of both non‐OA knee and non‐OA hip chondrocytes led to a significant increase in expression of each of the 3 lincRNAs (Figure 4C).

### Negative regulation of the IL‐1β–stimulated production of proinflammatory cytokines in human chondrocytes by CILinc01 and CILinc02

**Figure 5:**
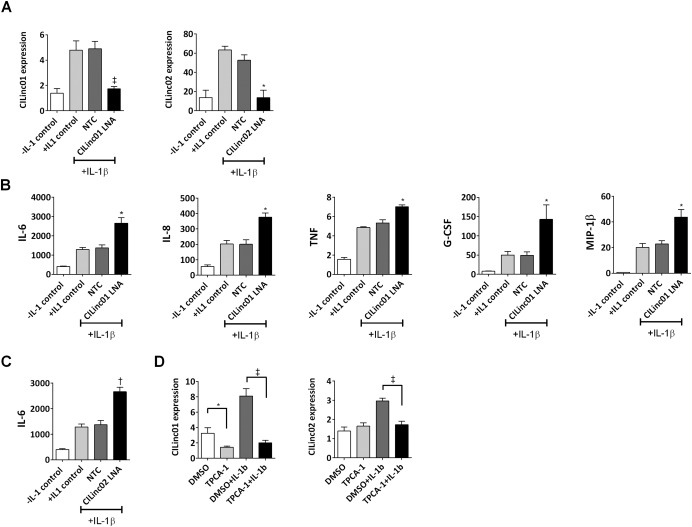
Long intergenic noncoding RNAs (lincRNAs) modulate the interleukin‐1β (IL‐1β)–stimulated induction of proinflammatory cytokines in human chondrocytes and are suppressed by IKK‐2 inhibition. **A,** Knockdown of IL‐1β–stimulated CILinc01 and CILinc02 human chondrocyte TC28 cells using LNA GapmeRs. TC28 cells were transfected overnight either with locked nucleic acids (LNAs) targeting CILinc01 or targeting CILinc02 or with a nontargeting control (NTC) LNA GapmeR. Following transfection, cells were left unstimulated or stimulated with IL‐1β (1 ng/ml) for 4 hours. Bars show the mean ± SEM. ∗ = *P* < 0.05; ‡ = *P* < 0.001, versus nontargeting control LNA–transfected cells. **B** and **C,** Concentration of cytokines (pg/ml) in supernatants from human chondrocytes transfected with either **B,** CILinc01 LNA or **C,** CILinc02 LNA and then stimulated with 1 ng/ml of IL‐1β for 4 hours. Bars show the mean ± SEM (n = 3 samples per group). ∗ = *P* < 0.05; † = *P* < 0.01, versus nontargeting control LNA–transfected cells. **D,** Suppression of the IL‐1β–stimulated induction of CILinc01 and CILinc02 in primary human OA chondrocytes preincubated with the IKK‐2 inhibitor TPCA‐1 (10 μ*M*). Bars show the mean ± SEM (n = 3 samples per group). ∗ = *P* < 0.05; ‡ = *P* < 0.001, by one‐way analysis of variance. TNF = tumor necrosis factor; G‐CSF = granulocyte colony‐stimulating factor; MIP‐1β = macrophage inflammatory protein 1β.

Given the association of CILinc01 and CILinc02 with the IL‐1β chondrocyte inflammatory response, and their down‐regulation in OA cartilage tissue, we speculated that CILinc01 and CILinc02 might mediate the production of proinflammatory cytokines. To test this hypothesis, we examined the effect of knockdown of CILinc01 and CILinc02 expression on the human chondrocyte inflammatory response. For these experiments, we used the human chondrocyte TC28 cell line, which when incubated in low serum (0.1% FCS) without stimulation expressed type II collagen (see Supplementary Figures 3A and B, available on the *Arthritis & Rheumatology* web site at http://onlinelibrary.wiley.com/doi/10.1002/art.39520/abstract). Similar to the findings in primary chondrocytes, IL‐1β stimulation of TC28 cells induced a rapid release of IL‐6 protein (Supplementary Figure 3C) and induction of MMPs and proinflammatory cytokines (Supplementary Figure 3D). TC28 cells were transfected with either LNA GapmeRs targeting CILinc01 or CILinc02, or a nontargeting control LNA GapmeR. Following 24 hours of transfection, cells were stimulated with IL‐1β for 4 hours in order to provoke an inflammatory response. Similar to our findings in primary human chondrocytes, 4 hours of exposure of the TC28 chondrocyte cell line to IL‐1β led to a significant induction of expression of CILinc01 and CILinc02. The IL‐1β–induced expression of CILinc01 was significantly reduced (by 63%) in chondrocytes transfected with an anti‐CILinc01 LNA GapmeR, and CILinc02 expression was significantly reduced (by 74%) in cells transfected with an anti‐CILinc02 GapmeR, compared to an LNA control sequence (Figure 5A).

We then investigated the effect of CILinc01 and CILinc02 knockdown on the inflammatory response, by measuring the secretion of a panel of 17 proinflammatory cytokines in response to 4 hours of IL‐1β stimulation of human chondrocytes (see Supplementary Table 5, available on the *Arthritis & Rheumatology* web site at http://onlinelibrary.wiley.com/doi/10.1002/art.39520/abstract). Knockdown of CILinc01 expression significantly enhanced the IL‐1β–stimulated production of IL‐6, IL‐8, TNF, MIP‐1β, and G‐CSF (Figure 5B), while knockdown of CILinc02 expression significantly enhanced the IL‐1β–stimulated production of IL‐6 (Figure 5C). Since previous studies have shown that NF‐κB activity can regulate the expression of lncRNAs, we then also examined the effect of pharmacologic inhibition of IKK‐2 on the IL‐1β–stimulated production of CILinc01 and CILinc02. To this end we used TPCA‐1, a known IKK‐2 inhibitor 20, 32, 33. In cell‐free enzymatic assays, TPCA‐1 displays 22‐fold selectivity for IKK‐2 over IKK‐1 and a >550‐fold selectivity over other kinases, including MAP kinases and JNK kinases 32, though a recent study showed that in non–small cell lung cancer cell lines TPCA‐1 also inhibited STAT‐3 phosphorylation 34. Preincubation of primary chondrocytes with TPCA‐1 (10 μ*M*) significantly reduced the induction of both CILinc01 and CILinc02 that occurred after 4 hours of stimulation with IL‐1β. (Figure 5D).

## DISCUSSION

This study is the first to use RNAseq to determine the profile of lncRNA expression in primary human OA chondrocytes and has resulted in the cataloging of 983 lncRNAs, including members of the lincRNA, antisense RNA, and pseudogene families. Importantly, we have identified 158 lincRNAs and 25 antisense RNAs that are absent from Gencode version 19 35 and the HumanBodyMap lncRNA catalog 29, and might therefore be unique to chondrocytes and have a cell‐specific function. In addition, this study is the first to examine the changes in lncRNA levels that are associated with the inflammatory response in human chondrocytes. In this regard, 125 lncRNAs were differentially expressed upon IL‐1β stimulation of human OA chondrocytes. Of relevance, Fu et al 22 recently showed a catalog of 4,714 lncRNAs found by microarray analysis to be differentially expressed in knee OA patients compared to non‐OA cartilage. In our RNAseq chondrocyte analysis, if we included lncRNAs with a *P* value of less than 0.05 (rather than an FDR optimized q of <0.05), which was the inclusion criterion used by Fu et al 22, 7 of these lncRNAs (namely, ENST00000426066, ENST00000369884, ENST00000419463, ENST00000421237, ENST00000412485, ENST00000455607, and ENST00000418242) were differentially expressed in chondrocytes upon IL‐1β stimulation. This relatively low number of lncRNAs in common is likely due to differences in conditions (IL‐1β stimulation of chondrocytes versus end‐stage cartilage disease comparison) and methodologic approach (sequencing versus microarrays). As an example, the microarray studies by Fu et al 22 would not have detected changes in PACER, CILinc01, and CILinc02 since these are novel transcripts for which there are no microarray probes. Despite these differences, we speculate that these shared lncRNAs might have a function in OA, which would warrant further investigation.

Importantly, there is now evidence that lncRNAs regulate in *cis* local mRNA expression 21, 36. Indeed, among those lncRNAs differentially expressed upon IL‐1β stimulation was the lincRNA PACER 23, which is located adjacent to and upstream of the gene PTGS2 (COX‐2) and has been shown to regulate PTGS2 production 23. As shown in the present study, PACER appears to be transcribed from the same promoter regions as PTGS2, which results in bidirectional production of both coding and noncoding RNA. Significantly, the majority of the inflammation‐associated lncRNAs we identified were found to be mRNA flanking, several of which (including PACER) were located close to genes relevant to either inflammation or cartilage biology, which could be indicative of a functional role in OA.

Given these observations, we selected PACER and 7 additional inflammation‐associated lincRNAs and proceeded to investigate their potential clinical relevance by comparing their expression in articular hip and knee cartilage obtained from both OA and non‐OA patients. Notably, all 8 of the inflammation‐associated lincRNAs were found to be significantly down‐regulated in hip OA cartilage, while only PACER, CILinc01, and CILinc02 were also down‐regulated in knee OA cartilage. This could indicate that these lincRNAs perform protective roles in preventing inflammation‐mediated cartilage degeneration, but also suggests that there are anatomic site–specific differences in OA cartilage at the level of lncRNA expression. Indeed, a recent report described epigenetic differences between knee and hip OA cartilage based on DNA methylation analyses 37, and previous studies have shown differences in dysregulated mRNA transcripts and pathways between knee OA and hip OA cartilage 38. It should be noted that there were differences in K/L grade between our hip OA and knee OA patients. However, all of our knee OA samples were K/L grade 4, while our hip OA samples were either K/L grade 3 or K/L grade 4, so it would appear unlikely that the differential expression of all 8 inflammation‐associated lncRNAs in hip OA cartilage was due to differences in OA severity.

Subsequent experiments demonstrated that PACER, CILinc01, and CILinc02 were induced in OA chondrocytes by multiple proinflammatory cytokines, which have been reported to be elevated in either OA sera or OA synovial fluid (TNF, visfatin, and leptin as well as IL‐1β). Importantly, the induction of chondrocyte lincRNA expression in response to multiple proinflammatory stimuli was rapid and transient, as might be expected if they were key regulators of the inflammatory response in joint cartilage.

Given that PACER has previously been shown to positively regulate PTGS2 production 23 and that PTGS2 is associated with inflammation, we were initially surprised to discover that PACER was down‐regulated in hip OA cartilage. However, there are reports that PTGS2 expression in OA synovial tissue is significantly lower in late OA compared to early OA 39, suggesting it may play a different role in established human OA. Furthermore, PTGS2 has also been implicated as having an antiinflammatory functional role 40, since the release of prostaglandin D_2_ (PGD_2_) and its breakdown product PGDJ_2_ are associated with the resolution of inflammation 41. Indeed, in stark contrast to their efficacy in blocking proinflammatory responses, inhibitors of COX‐2 have been shown to delay the resolution of inflammation 42. Therefore, the decreased expression of PACER we observed in human hip OA cartilage could represent a pathologic reduction in the ability of the cartilage tissue to resolve aberrant inflammation.

The functional significance of our finding that CILinc02 is down‐regulated in human hip OA cartilage is unclear. Studies in rheumatoid arthritis suggest that IL‐7 (the nearest coding gene to CILinc02) contributes to inflammation 43 and mediates the production of TNF 44, while in OA, IL‐7 has been reported to induce MMP‐13 and proteoglycan loss from cartilage, suggesting that it may promote cartilage degeneration 45. However, we did not detect expression of the IL‐7 gene in either OA or non‐OA hip cartilage samples.

Functional studies to determine the roles of CILinc01 and CILinc02 showed that knockdown of their expression in human chondrocytes significantly increased the IL‐1β–stimulated production of several proinflammatory cytokines, including IL‐6, suggesting that CILinc01 and CILinc02 may negatively regulate the chondrocyte inflammatory response. It is significant, therefore, that we found decreased expression of CILinc01 and CILinc02 in both knee OA and hip OA cartilage compared to normal healthy cartilage, since this could indicate that down‐regulation of CILinc01 and CILinc02 in human articular cartilage leads to an inability to regulate inflammation in the joint. Of interest, the nearest coding gene to CILinc01 is HIVEP2 (also known as Schnurri‐2), which has previously been reported to be a negative regulator of allergic airway inflammation via repression of NF‐κB activity 46, as well as being implicated in mediating chondrocyte differentiation 47. Therefore, it is conceivable that the observed effects of CILinc01 on chondrocyte cytokine production are mediated via repression of NF‐κB activity through modulation of HIVEP2 gene expression. Of note, stimulation of primary chondrocytes with an IKK‐2 inhibitor blocked the IL‐1β–stimulated production of both CILinc01 and CIlinc02, suggesting that NF‐κB activity may regulate their expression in chondrocytes.

In conclusion, these data signify that CILinc01 and CILinc02 may play an important physiologic role in regulating the pathologic response to inflammation within the OA joint, and that its down‐regulation in both knee and hip OA cartilage could contribute to inflammation‐driven cartilage degeneration. Clearly, future studies to determine the mode of action of CILinc01 and CILinc02 as well as other chondrocyte inflammation–associated lincRNAs in mediating OA cartilage pathology and inflammation are warranted and may lead to the identification of novel targets for the development of therapeutic agents.

## AUTHOR CONTRIBUTIONS

All authors were involved in drafting the article or revising it critically for important intellectual content, and all authors approved the final version to be published. Dr. Jones had full access to all of the data in the study and takes responsibility for the integrity of the data and the accuracy of the data analysis.

### Study conception and design

Pearson, Lindsay, Jones.

### Acquisition of data

Pearson, Philp, Heward, Roux, Walsh, Davis, Lindsay, Jones.

### Analysis and interpretation of data

Pearson, Philp, Heward, Roux, Davis, Lindsay, Jones.

## Supporting information


**Supplementary Figure 1.** Expression of GAPDH in (**A**) non‐OA hip (n=6) and OA hip (n=9), and (**B**) non‐OA knee (n=4) and OA knee (n=12) cartilage tissue. Expression was normalised to 18S using the ΔΔCt method.

**Supplementary Figure 2.** Release of MMP13 following 4h and 24h IL‐1β (1ng/m1) stimulation of primary OA chondrocytes as measured by ELISA. Bars represent mean ± SEM (n=3). ‡= *P* < 0.001, significantly different from control.

**Supplementary Figure 3.** (**A**) Relative expression of COL2A1 in freshly isolated primary human chondrocytes (P0) and TC28s. **(B)** Western blot showing COL2A1 (∼55 kDa) in freshly isolated primary chondrocytes (P0) and passage 2 (P2) chondrocytes and TC28s. Mouse monoclonal anti‐COL2A1 (Sigma SAB1403684) was used at 1:1000 dilutiion. (**C**) IL‐6 cytokine release in response to 4h **IL‐1β** stimulation of primary OA chondrocytes and TC28s, as measured by ELISA. **(D)** Induction of pro‐inflammatory cytokines and MMPs in response to 4h **IL‐1β** stimulation of TC28s. Bars represent mean ± SEM, *=*P* < 0.05, †=*P* < 0.01, significantly different from control.

**Supplementary Table 1. Patient demographics.** X‐ray radiographs were assessed by a clinician to determined KL grade. X‐ray radiographs were not available for post‐mortem subjects.

Supplementary Table 2: Differentially expressed protein coding transcripts upon 4h IL‐1β stimulation of primary chondrocytes

Supplementary Table 3

Supplementary Table 4

**Supplementary Table 5.** Human Bio‐plex 17‐plex data following 4h IL‐1β stimulation of human TC28 chondrocytes transfected with LNA GAPmeRs. Fold change is shown for those analytes which were significantly different (*P* < 0.05) relative to the non‐targeting control LNA. Data which were not statistically significant are shown as ‘*NS*’ whilst analytes which were not detectable are shown as ‘n.d’. IL‐1β (1ng/ml) was used as the cell stimulant and thus was discounted from these data.
