# Supplementary material for: Long Intergenic Noncoding RNAs Mediate the Human Chondrocyte Inflammatory Response and Are Differentially Expressed in Osteoarthritis Cartilage
Source: Arthritis Rheumatol. 2016 Mar 28;68(4):845–56. doi: 10.1002/art.39520 (PMC4950001; doi:10.1002/art.39520)
Supplement: Supplementary file 4 — Supplementary Table 1. Patient demographics. X‐ray radiographs were assessed by a clinician to determined KL grade. X‐ray radiographs were not available for post‐mortem subjects. [file ART-68-845-s004.docx]

**Supplementary Table 1. Patient demographics.**  X-ray radiographs were assessed by a clinician to determined KL grade. X-ray radiographs were not available for post-mortem subjects.

| **Patient Group** | **Age**  **(mean ± SEM)** | **Gender**  **(% male, % female)** | **KL Grade** | | | | |
| --- | --- | --- | --- | --- | --- | --- | --- |
|  |  |  | **0** | **1** | **2** | **3** | **4** |
| OA knee | 70 ± 3 years | 77%, 23% | - | - | - | - | 100% |
| Non-OA knee (Post-mortem) | 74 ± 5 years | 75%, 25% |  |  |  |  |  |
| OA Hip | 69 ± 3 years | 40%, 60% | - | - | - | 40% | 60% |
| Non-OA hip (NOF fracture) | 74 ± 2 years | 50%, 50% | 100% | - | - | - | - |
