# Supplementary material for: Long Intergenic Noncoding RNAs Mediate the Human Chondrocyte Inflammatory Response and Are Differentially Expressed in Osteoarthritis Cartilage
Source: Arthritis Rheumatol. 2016 Mar 28;68(4):845–56. doi: 10.1002/art.39520 (PMC4950001; doi:10.1002/art.39520)
Supplement: Supplementary file 5 — Supplementary Table 2: Differentially expressed protein coding transcripts upon 4h IL‐1β stimulation of primary chondrocytes [file ART-68-845-s005.docx]

| **Gene_ID**  **Supplementary Table 2: Differentially expressed protein coding transcripts upon 4h IL-1β stimulation of primary chondrocytes** | **Gene** | **Position** | **FPKM (Control)** | **FPKM (IL1b)** | **FPKM (Absolute Change)** | **Log2**  **(fold change)** | **p-value** | **q-value** |
| --- | --- | --- | --- | --- | --- | --- | --- | --- |
| ENSG00000169429.6 | IL8 | chr4:74606222-74609433 | 13.1643 | 3769.68 | 3756.5157 | 8.16166 | 5.00E-05 | 0.00217928 |
| ENSG00000112096.12 | SOD2 | chr6:160090088-160210781 | 512.342 | 4164.61 | 3652.268 | 3.023 | 5.00E-05 | 0.00217928 |
| ENSG00000115009.7 | CCL20 | chr2:228678557-228682272 | 15.5551 | 3304.71 | 3289.1549 | 7.73099 | 5.00E-05 | 0.00217928 |
| ENSG00000123610.3 | TNFAIP6 | chr2:152214105-152236560 | 201.025 | 2987.76 | 2786.735 | 3.89362 | 5.00E-05 | 0.00217928 |
| ENSG00000162692.6 | VCAM1 | chr1:101185297-101204601 | 146.381 | 2671.66 | 2525.279 | 4.18994 | 5.00E-05 | 0.00217928 |
| ENSG00000149968.7 | MMP3 | chr11:102706531-102714534 | 434.193 | 2118.32 | 1684.127 | 2.28651 | 5.00E-05 | 0.00217928 |
| ENSG00000108691.5 | CCL2 | chr17:32581899-32584222 | 47.6211 | 1617.14 | 1569.5189 | 5.0857 | 5.00E-05 | 0.00217928 |
| ENSG00000073756.7 | PTGS2 | chr1:186640922-186649559 | 80.9176 | 1209.99 | 1129.0724 | 3.9024 | 5.00E-05 | 0.00217928 |
| ENSG00000196611.4 | MMP1 | chr11:102660650-102668891 | 126.055 | 1239.49 | 1113.435 | 3.29762 | 5.00E-05 | 0.00217928 |
| ENSG00000164761.4 | TNFRSF11B | chr8:119935795-119964439 | 231.444 | 1160.26 | 928.816 | 2.32571 | 5.00E-05 | 0.00217928 |
| ENSG00000136244.7 | IL6 | chr7:22765502-22771621 | 2.80295 | 846.417 | 843.61405 | 8.23828 | 5.00E-05 | 0.00217928 |
| ENSG00000090339.4 | ICAM1 | chr19:10381510-10397291 | 17.202 | 808.62 | 791.418 | 5.55481 | 5.00E-05 | 0.00217928 |
| ENSG00000125148.6 | MT2A | chr16:56642110-56643409 | 366.737 | 1153.47 | 786.733 | 1.65315 | 5.00E-05 | 0.00217928 |
| ENSG00000163739.4 | CXCL1 | chr4:74735109-74736959 | 2.14248 | 758.694 | 756.55152 | 8.46809 | 5.00E-05 | 0.00217928 |
| **Gene_ID** | **Gene** | **Position** | **FPKM (Control)** | **FPKM (IL1b)** | **FPKM (Absolute Change)** | **Log2**  **(fold change)** | **p-value** | **q-value** |
| ENSG00000185215.4 | TNFAIP2 | chr14:103589778-103603776 | 18.3565 | 753.903 | 735.5465 | 5.36002 | 5.00E-05 | 0.00217928 |
| ENSG00000122641.9 | INHBA | chr7:41724711-41742706 | 58.6001 | 735.909 | 677.3089 | 3.65055 | 5.00E-05 | 0.00217928 |
| ENSG00000123689.5 | G0S2 | chr1:209848764-209849733 | 9.92855 | 676.483 | 666.55445 | 6.09033 | 5.00E-05 | 0.00217928 |
| ENSG00000145779.7 | TNFAIP8 | chr5:118604386-118735383 | 26.8687 | 618.06 | 591.1913 | 4.52375 | 5.00E-05 | 0.00217928 |
| ENSG00000003989.12 | SLC7A2 | chr8:17354596-17428082 | 50.179 | 636.481 | 586.302 | 3.66496 | 5.00E-05 | 0.00217928 |
| ENSG00000130066.12 | SAT1 | chrX:23801289-23804343 | 385.621 | 932.953 | 547.332 | 1.27462 | 5.00E-05 | 0.00217928 |
| ENSG00000115963.9 | RND3 | chr2:151324708-151395525 | 189.103 | 704.02 | 514.917 | 1.89645 | 5.00E-05 | 0.00217928 |
| ENSG00000100906.6 | NFKBIA | chr14:35870716-35873955 | 10.7855 | 480.534 | 469.7485 | 5.47748 | 5.00E-05 | 0.00217928 |
| ENSG00000128342.4 | LIF | chr22:30636435-30642840 | 11.5671 | 461.869 | 450.3019 | 5.31939 | 5.00E-05 | 0.00217928 |
| ENSG00000163661.3 | PTX3 | chr3:156977530-157251408 | 20.8161 | 458.431 | 437.6149 | 4.46094 | 5.00E-05 | 0.00217928 |
| ENSG00000081041.8 | CXCL2 | chr4:74962751-74965010 | 0.804859 | 396.74 | 395.935141 | 8.94524 | 5.00E-05 | 0.00217928 |
| ENSG00000196136.12 | SERPINA3 | chr14:95027427-95090983 | 162.391 | 518.381 | 355.99 | 1.67454 | 5.00E-05 | 0.00217928 |
| ENSG00000184371.9 | CSF1 | chr1:110452863-110473614 | 23.4627 | 301.659 | 278.1963 | 3.68448 | 5.00E-05 | 0.00217928 |
| ENSG00000115738.5 | ID2 | chr2:8818974-8824583 | 13.4048 | 286.673 | 273.2682 | 4.41859 | 5.00E-05 | 0.00217928 |
| **Gene_ID** | **Gene** | **Position** | **FPKM (Control)** | **FPKM (IL1b)** | **FPKM (Absolute Change)** | **Log2**  **(fold change)** | **p-value** | **q-value** |
| ENSG00000172331.7 | BPGM | chr7:134331559-134364565 | 53.4396 | 315.406 | 261.9664 | 2.56123 | 5.00E-05 | 0.00217928 |
| ENSG00000124145.5 | SDC4 | chr20:43953927-43977064 | 92.5389 | 333.37 | 240.8311 | 1.84899 | 5.00E-05 | 0.00217928 |
| ENSG00000138685.8 | FGF2 | chr4:123747862-123844123 | 65.9531 | 304.02 | 238.0669 | 2.20465 | 5.00E-05 | 0.00217928 |
| ENSG00000109320.7 | NFKB1 | chr4:103422485-103538459 | 27.985 | 265.807 | 237.822 | 3.24765 | 5.00E-05 | 0.00217928 |
| ENSG00000151632.12 | AKR1C2 | chr10:5029966-5060223 | 90.0211 | 313.084 | 223.0629 | 1.79822 | 0.0004 | 0.0133617 |
| ENSG00000144476.5 | ACKR3 | chr2:237476429-237491001 | 91.3085 | 302.897 | 211.5885 | 1.73 | 5.00E-05 | 0.00217928 |
| ENSG00000197249.8 | SERPINA1 | chr14:94843083-94857030 | 90.6159 | 291.957 | 201.3411 | 1.68792 | 5.00E-05 | 0.00217928 |
| ENSG00000104635.9 | SLC39A14 | chr8:22224761-22291642 | 70.6751 | 270.878 | 200.2029 | 1.93837 | 5.00E-05 | 0.00217928 |
| ENSG00000105825.7 | TFPI2 | chr7:93220884-93540577 | 17.7123 | 205.831 | 188.1187 | 3.53863 | 5.00E-05 | 0.00217928 |
| ENSG00000007908.11 | SELE | chr1:169631244-169863408 | 0.371295 | 186.478 | 186.106705 | 8.97222 | 5.00E-05 | 0.00217928 |
| ENSG00000184588.13 | PDE4B | chr1:66258196-66840259 | 25.1627 | 188.576 | 163.4133 | 2.90579 | 0.0002 | 0.00739942 |
| ENSG00000007171.12 | NOS2 | chr17:26083791-26221778 | 0.511921 | 160.824 | 160.312079 | 8.29535 | 5.00E-05 | 0.00217928 |
| ENSG00000169908.6 | TM4SF1 | chr3:149086808-149095652 | 148.72 | 307.809 | 159.089 | 1.04943 | 0.00025 | 0.0089375 |
| ENSG00000164283.8 | ESM1 | chr5:54273691-54318499 | 4.87143 | 162.824 | 157.95257 | 5.06283 | 5.00E-05 | 0.00217928 |
| **Gene_ID** | **Gene** | **Position** | **FPKM (Control)** | **FPKM (IL1b)** | **FPKM (Absolute Change)** | **Log2**  **(fold change)** | **p-value** | **q-value** |
| ENSG00000169715.10 | MT1E | chr16:56659386-56661024 | 94.8176 | 249.069 | 154.2514 | 1.39332 | 5.00E-05 | 0.00217928 |
| ENSG00000125845.6 | BMP2 | chr20:6748310-6760927 | 63.6705 | 212.071 | 148.4005 | 1.73585 | 5.00E-05 | 0.00217928 |
| ENSG00000116690.7 | PRG4 | chr1:186265404-186344825 | 65.6054 | 212.611 | 147.0056 | 1.69633 | 0.0017 | 0.044065 |
| ENSG00000144802.7 | NFKBIZ | chr3:101498045-101579866 | 23.8783 | 165.91 | 142.0317 | 2.79662 | 5.00E-05 | 0.00217928 |
| ENSG00000023445.9 | BIRC3 | chr11:102188214-102210134 | 3.57278 | 138.615 | 135.04222 | 5.27789 | 5.00E-05 | 0.00217928 |
| ENSG00000229644.4 | NAMPTL | chr10:36810648-36813162 | 42.5346 | 176.244 | 133.7094 | 2.05087 | 5.00E-05 | 0.00217928 |
| ENSG00000111912.14 | NCOA7 | chr6:126102306-126252266 | 26.0668 | 159.693 | 133.6262 | 2.61502 | 5.00E-05 | 0.00217928 |
| ENSG00000118503.10 | TNFAIP3 | chr6:138188350-138204449 | 4.53192 | 130.11 | 125.57808 | 4.84346 | 5.00E-05 | 0.00217928 |
| ENSG00000116285.8 | ERRFI1 | chr1:8064463-8086368 | 81.1734 | 204.86 | 123.6866 | 1.33556 | 5.00E-05 | 0.00217928 |
| ENSG00000140379.7 | BCL2A1 | chr15:80253230-80263788 | 3.33959 | 126.04 | 122.70041 | 5.23806 | 5.00E-05 | 0.00217928 |
| ENSG00000145777.10 | TSLP | chr5:110405759-110413722 | 1.12533 | 123.254 | 122.12867 | 6.77514 | 5.00E-05 | 0.00217928 |
| ENSG00000163734.4 | CXCL3 | chr4:74902305-74904524 | 0.288671 | 122.285 | 121.996329 | 8.7266 | 5.00E-05 | 0.00217928 |
| ENSG00000104312.6 | RIPK2 | chr8:90769974-90803291 | 8.74736 | 127.358 | 118.61064 | 3.8639 | 5.00E-05 | 0.00217928 |
| ENSG00000159200.13 | RCAN1 | chr21:35885439-35987441 | 57.0623 | 174.017 | 116.9547 | 1.60862 | 5.00E-05 | 0.00217928 |
| **Gene_ID** | **Gene** | **Position** | **FPKM (Control)** | **FPKM (IL1b)** | **FPKM (Absolute Change)** | **Log2**  **(fold change)** | **p-value** | **q-value** |
| ENSG00000187479.4 | C11orf96 | chr11:43946891-43965888 | 6.1684 | 118.367 | 112.1986 | 4.26223 | 5.00E-05 | 0.00217928 |
| ENSG00000122862.4 | SRGN | chr10:70847861-70864567 | 37.4361 | 144.265 | 106.8289 | 1.94622 | 5.00E-05 | 0.00217928 |
| ENSG00000159231.5 | CBR3 | chr21:37507209-37518864 | 16.376 | 118.888 | 102.512 | 2.85995 | 5.00E-05 | 0.00217928 |
| ENSG00000070961.10 | ATP2B1 | chr12:89981827-90103077 | 36.4465 | 138.861 | 102.4145 | 1.92979 | 0.00095 | 0.0276674 |
| ENSG00000107968.5 | MAP3K8 | chr10:30722865-30750762 | 10.3968 | 112.653 | 102.2562 | 3.43767 | 5.00E-05 | 0.00217928 |
| ENSG00000143333.6 | RGS16 | chr1:182567757-182573543 | 11.7444 | 113.952 | 102.2076 | 3.27839 | 5.00E-05 | 0.00217928 |
| ENSG00000110330.4 | BIRC2 | chr11:102217941-102249401 | 49.9012 | 151.979 | 102.0778 | 1.60673 | 0.0001 | 0.00409228 |
| ENSG00000056558.6 | TRAF1 | chr9:123664670-123691451 | 1.53375 | 101.392 | 99.85825 | 6.04674 | 5.00E-05 | 0.00217928 |
| ENSG00000112715.16 | VEGFA | chr6:43737920-43754224 | 41.0787 | 136.995 | 95.9163 | 1.73766 | 5.00E-05 | 0.00217928 |
| ENSG00000159388.5 | BTG2 | chr1:203274618-203278730 | 11.5707 | 103.07 | 91.4993 | 3.15508 | 5.00E-05 | 0.00217928 |
| ENSG00000145901.10 | TNIP1 | chr5:150409505-150473138 | 26.3588 | 113.236 | 86.8772 | 2.10298 | 5.00E-05 | 0.00217928 |
| ENSG00000086061.11 | DNAJA1 | chr9:33025208-33039905 | 72.905 | 158.962 | 86.057 | 1.12459 | 5.00E-05 | 0.00217928 |
| ENSG00000162645.8 | GBP2 | chr1:89571814-89641723 | 24.2657 | 106.831 | 82.5653 | 2.13834 | 5.00E-05 | 0.00217928 |
| ENSG00000159128.10 | IFNGR2 | chr21:34775201-34852318 | 21.6322 | 102.736 | 81.1038 | 2.24769 | 5.00E-05 | 0.00217928 |
| **Gene_ID** | **Gene** | **Position** | **FPKM (Control)** | **FPKM (IL1b)** | **FPKM (Absolute Change)** | **Log2**  **(fold change)** | **p-value** | **q-value** |
| ENSG00000083799.13 | CYLD | chr16:50775960-50835846 | 24.6046 | 103.484 | 78.8794 | 2.0724 | 5.00E-05 | 0.00217928 |
| ENSG00000131459.8 | GFPT2 | chr5:179727689-179780387 | 19.6049 | 96.1469 | 76.542 | 2.29403 | 5.00E-05 | 0.00217928 |
| ENSG00000085662.9 | AKR1B1 | chr7:134127101-134144036 | 34.942 | 109.102 | 74.16 | 1.64264 | 5.00E-05 | 0.00217928 |
| ENSG00000145632.10 | PLK2 | chr5:57749808-57756087 | 15.8369 | 88.4754 | 72.6385 | 2.48199 | 5.00E-05 | 0.00217928 |
| ENSG00000137745.7 | MMP13 | chr11:102813723-102826463 | 12.4501 | 84.509 | 72.0589 | 2.76294 | 5.00E-05 | 0.00217928 |
| ENSG00000124762.9 | CDKN1A | chr6:36644304-36655116 | 66.838 | 138.298 | 71.46 | 1.04904 | 0.00015 | 0.00576752 |
| ENSG00000167034.9 | NKX3-1 | chr8:23536205-23540440 | 4.68731 | 76.1242 | 71.43689 | 4.02152 | 5.00E-05 | 0.00217928 |
| ENSG00000146278.10 | PNRC1 | chr6:89790469-89794879 | 34.1082 | 105.177 | 71.0688 | 1.62463 | 5.00E-05 | 0.00217928 |
| ENSG00000117226.7 | GBP3 | chr1:89472348-89488577 | 24.6093 | 90.2986 | 65.6893 | 1.8755 | 5.00E-05 | 0.00217928 |
| ENSG00000105835.7 | NAMPT | chr7:105888730-105926772 | 21.0973 | 82.6928 | 61.5955 | 1.9707 | 0.0009 | 0.0265761 |
| ENSG00000185022.7 | MAFF | chr22:38507501-38612518 | 22.3356 | 80.6386 | 58.303 | 1.85213 | 5.00E-05 | 0.00217928 |
| ENSG00000125347.9 | IRF1 | chr5:131817300-131826490 | 3.45639 | 59.6566 | 56.20021 | 4.10934 | 5.00E-05 | 0.00217928 |
| ENSG00000152484.9 | USP12 | chr13:27640292-27746033 | 13.6249 | 69.1654 | 55.5405 | 2.34381 | 5.00E-05 | 0.00217928 |
| ENSG00000113369.4 | ARRDC3 | chr5:90664540-90679176 | 44.7814 | 100.266 | 55.4846 | 1.16287 | 5.00E-05 | 0.00217928 |
| **Gene_ID** | **Gene** | **Position** | **FPKM (Control)** | **FPKM (IL1b)** | **FPKM (Absolute Change)** | **Log2**  **(fold change)** | **p-value** | **q-value** |
| ENSG00000100219.12 | XBP1 | chr22:29190542-29196585 | 45.7639 | 100.088 | 54.3241 | 1.12899 | 5.00E-05 | 0.00217928 |
| ENSG00000186480.8 | INSIG1 | chr7:155089485-155101945 | 40.7269 | 94.9592 | 54.2323 | 1.22133 | 5.00E-05 | 0.00217928 |
| ENSG00000117525.9 | F3 | chr1:94994780-95007356 | 3.47496 | 53.1415 | 49.66654 | 3.93477 | 5.00E-05 | 0.00217928 |
| ENSG00000173193.9 | PARP14 | chr3:122399464-122449687 | 23.9304 | 71.2871 | 47.3567 | 1.57479 | 0.00165 | 0.0430319 |
| ENSG00000187193.8 | MT1X | chr16:56716335-56718108 | 44.1134 | 91.4114 | 47.298 | 1.05116 | 0.0001 | 0.00409228 |
| ENSG00000173559.8 | NABP1 | chr2:192542793-192561385 | 16.8962 | 63.7005 | 46.8043 | 1.9146 | 5.00E-05 | 0.00217928 |
| ENSG00000131979.14 | GCH1 | chr14:55308725-55369570 | 3.05726 | 49.7828 | 46.72554 | 4.02533 | 5.00E-05 | 0.00217928 |
| ENSG00000087074.7 | PPP1R15A | chr19:49375648-49379314 | 25.8974 | 72.1468 | 46.2494 | 1.47813 | 5.00E-05 | 0.00217928 |
| ENSG00000184557.3 | SOCS3 | chr17:76352863-76356158 | 2.5138 | 48.4258 | 45.912 | 4.26784 | 5.00E-05 | 0.00217928 |
| ENSG00000131669.5 | NINJ1 | chr9:95883770-95896570 | 10.6213 | 55.9586 | 45.3373 | 2.3974 | 5.00E-05 | 0.00217928 |
| ENSG00000117394.15 | SLC2A1 | chr1:43391051-43424530 | 5.56476 | 50.3959 | 44.83114 | 3.17892 | 5.00E-05 | 0.00217928 |
| ENSG00000110047.13 | EHD1 | chr11:64619113-64655768 | 17.2932 | 61.1276 | 43.8344 | 1.82162 | 5.00E-05 | 0.00217928 |
| ENSG00000113070.6 | HBEGF | chr5:139712427-139726216 | 4.89 | 47.9488 | 43.0588 | 3.29359 | 5.00E-05 | 0.00217928 |
| ENSG00000112149.5 | CD83 | chr6:14117871-14137149 | 0.995691 | 42.9982 | 42.002509 | 5.43244 | 5.00E-05 | 0.00217928 |
| **Gene_ID** | **Gene** | **Position** | **FPKM (Control)** | **FPKM (IL1b)** | **FPKM (Absolute Change)** | **Log2**  **(fold change)** | **p-value** | **q-value** |
| ENSG00000006459.6 | JHDM1D | chr7:139784545-139876835 | 15.2956 | 56.7649 | 41.4693 | 1.89188 | 0.00055 | 0.0176764 |
| ENSG00000163638.9 | ADAMTS9 | chr3:64501332-64673676 | 3.01833 | 42.0854 | 39.06707 | 3.8015 | 5.00E-05 | 0.00217928 |
| ENSG00000117228.9 | GBP1 | chr1:89518001-89531043 | 19.4558 | 58.4587 | 39.0029 | 1.58722 | 5.00E-05 | 0.00217928 |
| ENSG00000124882.3 | EREG | chr4:75230859-75254468 | 0.802474 | 39.7309 | 38.928426 | 5.62966 | 5.00E-05 | 0.00217928 |
| ENSG00000010818.4 | HIVEP2 | chr6:143072603-143266338 | 11.4022 | 50.2012 | 38.799 | 2.1384 | 0.00015 | 0.00576752 |
| ENSG00000125733.13 | TRIP10 | chr19:6737935-6751537 | 15.1145 | 52.5074 | 37.3929 | 1.79659 | 5.00E-05 | 0.00217928 |
| ENSG00000116514.12 | RNF19B | chr1:33402045-33430286 | 9.80566 | 46.8471 | 37.04144 | 2.25627 | 5.00E-05 | 0.00217928 |
| ENSG00000114784.3 | EIF1B | chr3:40351174-40353915 | 17.8019 | 54.8416 | 37.0397 | 1.62324 | 5.00E-05 | 0.00217928 |
| ENSG00000185947.10 | ZNF267 | chr16:31885078-31928668 | 9.09362 | 45.7479 | 36.65428 | 2.33078 | 5.00E-05 | 0.00217928 |
| ENSG00000011422.7 | PLAUR | chr19:44150246-44174699 | 25.4247 | 60.4875 | 35.0628 | 1.2504 | 5.00E-05 | 0.00217928 |
| ENSG00000171223.4 | JUNB | chr19:12873816-12985765 | 8.41147 | 43.0936 | 34.68213 | 2.35705 | 5.00E-05 | 0.00217928 |
| ENSG00000177606.5 | JUN | chr1:59246464-59249785 | 9.77579 | 44.2988 | 34.52301 | 2.17998 | 5.00E-05 | 0.00217928 |
| ENSG00000185650.8 | ZFP36L1 | chr14:69254376-69263190 | 16.9695 | 50.8335 | 33.864 | 1.58284 | 5.00E-05 | 0.00217928 |
| ENSG00000147454.9 | SLC25A37 | chr8:23386317-23432976 | 16.3558 | 49.6589 | 33.3031 | 1.60225 | 0.00025 | 0.0089375 |
| **Gene_ID** | **Gene** | **Position** | **FPKM (Control)** | **FPKM (IL1b)** | **FPKM (Absolute Change)** | **Log2**  **(fold change)** | **p-value** | **q-value** |
| ENSG00000139318.7 | DUSP6 | chr12:89741008-89747048 | 24.3692 | 57.5699 | 33.2007 | 1.24026 | 0.00135 | 0.037109 |
| ENSG00000120889.8 | TNFRSF10B | chr8:22570768-22926692 | 20.1796 | 52.8325 | 32.6529 | 1.38852 | 0.00055 | 0.0176764 |
| ENSG00000102554.9 | KLF5 | chr13:73629113-73651676 | 12.8256 | 45.3072 | 32.4816 | 1.82072 | 5.00E-05 | 0.00217928 |
| ENSG00000198805.7 | PNP | chr14:20937112-20945253 | 36.8315 | 68.9863 | 32.1548 | 0.90537 | 0.0017 | 0.044065 |
| ENSG00000126561.12 | STAT5A | chr17:40439564-40463961 | 3.41973 | 34.8012 | 31.38147 | 3.34718 | 5.00E-05 | 0.00217928 |
| ENSG00000118515.7 | SGK1 | chr6:134490383-134639250 | 22.6972 | 54.0212 | 31.324 | 1.25101 | 0.00015 | 0.00576752 |
| ENSG00000134070.4 | IRAK2 | chr3:10206548-10285427 | 2.73779 | 33.6734 | 30.93561 | 3.62053 | 5.00E-05 | 0.00217928 |
| ENSG00000137462.6 | TLR2 | chr4:154622651-154626851 | 1.7387 | 32.5777 | 30.839 | 4.2278 | 5.00E-05 | 0.00217928 |
| ENSG00000069493.10 | CLEC2D | chr12:9817564-9848413 | 2.38871 | 32.4513 | 30.06259 | 3.76397 | 5.00E-05 | 0.00217928 |
| ENSG00000114529.8 | C3orf52 | chr3:111805181-111852152 | 6.5158 | 36.3693 | 29.8535 | 2.48071 | 5.00E-05 | 0.00217928 |
| ENSG00000172602.5 | RND1 | chr12:49250927-49259681 | 0.534363 | 30.085 | 29.550637 | 5.81508 | 5.00E-05 | 0.00217928 |
| ENSG00000148926.5 | ADM | chr11:10326226-10328944 | 12.7253 | 42.2034 | 29.4781 | 1.72966 | 5.00E-05 | 0.00217928 |
| ENSG00000166394.10 | CYB5R2 | chr11:7686330-7698453 | 10.1495 | 39.324 | 29.1745 | 1.95401 | 5.00E-05 | 0.00217928 |
| ENSG00000146374.9 | RSPO3 | chr6:127439748-127518910 | 11.7884 | 40.8049 | 29.0165 | 1.79138 | 5.00E-05 | 0.00217928 |
| **Gene_ID** | **Gene** | **Position** | **FPKM (Control)** | **FPKM (IL1b)** | **FPKM (Absolute Change)** | **Log2**  **(fold change)** | **p-value** | **q-value** |
| ENSG00000137193.9 | PIM1 | chr6:37137978-37143202 | 2.84033 | 31.5751 | 28.73477 | 3.47466 | 5.00E-05 | 0.00217928 |
| ENSG00000120129.5 | DUSP1 | chr5:172195092-172198198 | 8.49103 | 37.1707 | 28.67967 | 2.13015 | 5.00E-05 | 0.00217928 |
| ENSG00000197329.7 | PELI1 | chr2:64319785-64371588 | 13.3956 | 41.6829 | 28.2873 | 1.63769 | 0.0005 | 0.0163167 |
| ENSG00000197622.8 | CDC42SE1 | chr1:151020215-151042801 | 20.4335 | 48.6612 | 28.2277 | 1.25183 | 5.00E-05 | 0.00217928 |
| ENSG00000163874.8 | ZC3H12A | chr1:37940152-37949980 | 0.736637 | 28.6235 | 27.886863 | 5.2801 | 5.00E-05 | 0.00217928 |
| ENSG00000162924.9 | REL | chr2:61108655-61158745 | 2.64947 | 29.5312 | 26.88173 | 3.47846 | 5.00E-05 | 0.00217928 |
| ENSG00000146232.10 | NFKBIE | chr6:44225902-44233500 | 5.49956 | 32.1106 | 26.61104 | 2.54566 | 5.00E-05 | 0.00217928 |
| ENSG00000162889.6 | MAPKAPK2 | chr1:206858288-206907628 | 15.2989 | 41.7342 | 26.4353 | 1.4478 | 0.00105 | 0.03003 |
| ENSG00000106258.9 | CYP3A5 | chr7:99245816-99277621 | 4.35094 | 30.6478 | 26.29686 | 2.81638 | 5.00E-05 | 0.00217928 |
| ENSG00000123609.6 | NMI | chr2:152126978-152146571 | 7.55368 | 33.7276 | 26.17392 | 2.15868 | 5.00E-05 | 0.00217928 |
| ENSG00000159110.15 | IFNAR2 | chr21:34602205-34669539 | 4.83885 | 30.6683 | 25.82945 | 2.66401 | 0.0002 | 0.00739942 |
| ENSG00000147853.12 | AK3 | chr9:4711154-4742043 | 13.3724 | 38.1421 | 24.7697 | 1.51213 | 0.0012 | 0.0336396 |
| ENSG00000187689.5 | AMTN | chr4:71384256-71398459 | 13.0612 | 37.7253 | 24.6641 | 1.53025 | 0.00025 | 0.0089375 |
| ENSG00000196878.8 | LAMB3 | chr1:209788214-209825811 | 1.83869 | 26.3211 | 24.48241 | 3.83947 | 5.00E-05 | 0.00217928 |
| **Gene_ID** | **Gene** | **Position** | **FPKM (Control)** | **FPKM (IL1b)** | **FPKM (Absolute Change)** | **Log2**  **(fold change)** | **p-value** | **q-value** |
| ENSG00000136048.9 | DRAM1 | chr12:102271128-102405908 | 22.9116 | 47.2296 | 24.318 | 1.04361 | 0.0009 | 0.0265761 |
| ENSG00000105855.5 | ITGB8 | chr7:20370324-20455377 | 12.1897 | 36.1323 | 23.9426 | 1.56762 | 0.0001 | 0.00409228 |
| ENSG00000122861.11 | PLAU | chr10:75668934-75682535 | 3.62202 | 27.1909 | 23.56888 | 2.90826 | 5.00E-05 | 0.00217928 |
| ENSG00000165434.6 | PGM2L1 | chr11:74041362-74109518 | 11.0662 | 34.2784 | 23.2122 | 1.63113 | 5.00E-05 | 0.00217928 |
| ENSG00000138166.5 | DUSP5 | chr10:112257595-112271302 | 2.22128 | 25.131 | 22.90972 | 3.50001 | 5.00E-05 | 0.00217928 |
| ENSG00000161570.4 | CCL5 | chr17:34198494-34207797 | 0.695635 | 23.4529 | 22.757265 | 5.0753 | 5.00E-05 | 0.00217928 |
| ENSG00000166920.6 | C15orf48 | chr15:45722726-45740959 | 0.647757 | 23.3276 | 22.679843 | 5.17044 | 5.00E-05 | 0.00217928 |
| ENSG00000149289.6 | ZC3H12C | chr11:109964086-110042566 | 4.68395 | 27.0492 | 22.36525 | 2.52979 | 5.00E-05 | 0.00217928 |
| ENSG00000107201.5 | DDX58 | chr9:32455299-32526322 | 10.5249 | 32.8458 | 22.3209 | 1.64191 | 0.00075 | 0.0228903 |
| ENSG00000155130.5 | MARCKS | chr6:114178540-114184648 | 20.3417 | 42.6507 | 22.309 | 1.06813 | 5.00E-05 | 0.00217928 |
| ENSG00000170961.6 | HAS2 | chr8:122624355-122653630 | 8.80966 | 31.0186 | 22.20894 | 1.81597 | 5.00E-05 | 0.00217928 |
| ENSG00000164932.8 | CTHRC1 | chr8:104383742-104395225 | 17.888 | 40.0363 | 22.1483 | 1.16232 | 0.0007 | 0.0215191 |
| ENSG00000173334.3 | TRIB1 | chr8:126442562-126450647 | 5.1879 | 27.26 | 22.0721 | 2.39356 | 5.00E-05 | 0.00217928 |
| ENSG00000168398.5 | BDKRB2 | chr14:96671015-96735304 | 2.22636 | 24.1805 | 21.95414 | 3.44108 | 5.00E-05 | 0.00217928 |
| **Gene_ID** | **Gene** | **Position** | **FPKM (Control)** | **FPKM (IL1b)** | **FPKM (Absolute Change)** | **Log2**  **(fold change)** | **p-value** | **q-value** |
| ENSG00000155287.6 | SLC25A28 | chr10:101370281-101380366 | 9.81436 | 31.7387 | 21.92434 | 1.69328 | 0.00035 | 0.0119103 |
| ENSG00000124479.8 | NDP | chrX:43808021-43832750 | 10.5728 | 32.3975 | 21.8247 | 1.61553 | 0.00065 | 0.020177 |
| ENSG00000133805.11 | AMPD3 | chr11:10329859-10529126 | 2.81863 | 24.4624 | 21.64377 | 3.1175 | 5.00E-05 | 0.00217928 |
| ENSG00000077150.13 | NFKB2 | chr10:104153866-104162281 | 4.32737 | 25.4606 | 21.13323 | 2.5567 | 5.00E-05 | 0.00217928 |
| ENSG00000101384.7 | JAG1 | chr20:10618331-10654694 | 9.76777 | 30.8708 | 21.10303 | 1.66014 | 5.00E-05 | 0.00217928 |
| ENSG00000177426.16 | TGIF1 | chr18:3411605-3458409 | 13.7228 | 34.7676 | 21.0448 | 1.34117 | 5.00E-05 | 0.00217928 |
| ENSG00000166750.5 | SLFN5 | chr17:33570054-33600674 | 12.7569 | 33.7654 | 21.0085 | 1.40427 | 5.00E-05 | 0.00217928 |
| ENSG00000125384.6 | PTGER2 | chr14:52781022-52795324 | 15.3267 | 36.2383 | 20.9116 | 1.24146 | 0.0003 | 0.0104605 |
| ENSG00000100739.6 | BDKRB1 | chr14:96671015-96735304 | 1.2164 | 21.9251 | 20.7087 | 4.1719 | 5.00E-05 | 0.00217928 |
| ENSG00000148344.10 | PTGES | chr9:132500609-132515326 | 4.1098 | 23.9907 | 19.8809 | 2.54534 | 5.00E-05 | 0.00217928 |
| ENSG00000006210.6 | CX3CL1 | chr16:57406369-57418960 | 0.383062 | 20.1411 | 19.758038 | 5.71642 | 5.00E-05 | 0.00217928 |
| ENSG00000119900.7 | OGFRL1 | chr6:71998505-72018653 | 7.06977 | 26.3974 | 19.32763 | 1.90066 | 5.00E-05 | 0.00217928 |
| ENSG00000101665.4 | SMAD7 | chr18:46446222-46477081 | 7.19558 | 26.5213 | 19.32572 | 1.88197 | 5.00E-05 | 0.00217928 |
| ENSG00000088826.13 | SMOX | chr20:4101626-4168394 | 5.11733 | 24.2977 | 19.18037 | 2.24736 | 5.00E-05 | 0.00217928 |
| **Gene_ID** | **Gene** | **Position** | **FPKM (Control)** | **FPKM (IL1b)** | **FPKM (Absolute Change)** | **Log2**  **(fold change)** | **p-value** | **q-value** |
| ENSG00000114019.10 | AMOTL2 | chr3:134074715-134094321 | 6.46775 | 25.6437 | 19.17595 | 1.98727 | 5.00E-05 | 0.00217928 |
| ENSG00000173918.10 | C1QTNF1 | chr17:77015290-77045870 | 2.32925 | 21.3775 | 19.04825 | 3.19815 | 5.00E-05 | 0.00217928 |
| ENSG00000173846.8 | PLK3 | chr1:45265896-45272957 | 3.44712 | 22.126 | 18.67888 | 2.68228 | 5.00E-05 | 0.00217928 |
| ENSG00000137393.8 | RNF144B | chr6:18368778-18469105 | 7.67353 | 26.216 | 18.54247 | 1.77249 | 0.00085 | 0.025454 |
| ENSG00000117594.5 | HSD11B1 | chr1:209859509-209908295 | 2.51557 | 20.875 | 18.35943 | 3.05282 | 5.00E-05 | 0.00217928 |
| ENSG00000164400.4 | CSF2 | chr5:131409482-131411859 | 0 | 18.1995 | 18.1995 | inf | 5.00E-05 | 0.00217928 |
| ENSG00000108688.7 | CCL7 | chr17:32597239-32599261 | 0.625827 | 18.5771 | 17.951273 | 4.89162 | 5.00E-05 | 0.00217928 |
| ENSG00000128335.9 | APOL2 | chr22:36622255-36636000 | 11.7654 | 29.4842 | 17.7188 | 1.32539 | 5.00E-05 | 0.00217928 |
| ENSG00000221869.4 | CEBPD | chr8:48649470-48651648 | 8.48125 | 25.7766 | 17.29535 | 1.60371 | 5.00E-05 | 0.00217928 |
| ENSG00000136158.6 | SPRY2 | chr13:80910110-80915086 | 14.4033 | 31.2686 | 16.8653 | 1.11832 | 5.00E-05 | 0.00217928 |
| ENSG00000198355.4 | PIM3 | chr22:50354160-50357728 | 1.66177 | 18.51 | 16.84823 | 3.47751 | 5.00E-05 | 0.00217928 |
| ENSG00000165195.9 | PIGA | chrX:15337572-15353676 | 2.62263 | 19.2588 | 16.63617 | 2.87643 | 5.00E-05 | 0.00217928 |
| ENSG00000120217.9 | CD274 | chr9:5450502-5470566 | 0.624873 | 17.1591 | 16.534227 | 4.77927 | 5.00E-05 | 0.00217928 |
| ENSG00000143479.11 | DYRK3 | chr1:206808880-206857764 | 8.0587 | 23.9454 | 15.8867 | 1.57113 | 0.00085 | 0.025454 |
| **Gene_ID** | **Gene** | **Position** | **FPKM (Control)** | **FPKM (IL1b)** | **FPKM (Absolute Change)** | **Log2**  **(fold change)** | **p-value** | **q-value** |
| ENSG00000104368.13 | PLAT | chr8:42032235-42065242 | 1.47331 | 17.286 | 15.81269 | 3.55247 | 5.00E-05 | 0.00217928 |
| ENSG00000179833.4 | SERTAD2 | chr2:64858754-64978139 | 8.68883 | 24.4837 | 15.79487 | 1.49459 | 0.00035 | 0.0119103 |
| ENSG00000146476.6 | C6orf211 | chr6:151773421-151791236 | 17.4284 | 33.173 | 15.7446 | 0.928571 | 0.0016 | 0.0420727 |
| ENSG00000175505.9 | CLCF1 | chr11:67131638-67141648 | 5.8531 | 21.5932 | 15.7401 | 1.8833 | 5.00E-05 | 0.00217928 |
| ENSG00000141682.11 | PMAIP1 | chr18:57567179-57571538 | 1.7634 | 17.4521 | 15.6887 | 3.30697 | 5.00E-05 | 0.00217928 |
| ENSG00000154736.5 | ADAMTS5 | chr21:28290230-28338832 | 12.9975 | 28.2931 | 15.2956 | 1.12222 | 0.00015 | 0.00576752 |
| ENSG00000163435.11 | ELF3 | chr1:201977072-201986316 | 0.106365 | 15.215 | 15.108635 | 7.16033 | 5.00E-05 | 0.00217928 |
| ENSG00000168685.10 | IL7R | chr5:35852796-35879705 | 4.53379 | 19.5297 | 14.99591 | 2.10688 | 5.00E-05 | 0.00217928 |
| ENSG00000095739.7 | BAMBI | chr10:28966270-28971868 | 9.51127 | 24.4017 | 14.89043 | 1.35927 | 5.00E-05 | 0.00217928 |
| ENSG00000119139.12 | TJP2 | chr9:71736208-71870124 | 7.45067 | 21.9808 | 14.53013 | 1.5608 | 5.00E-05 | 0.00217928 |
| ENSG00000058085.10 | LAMC2 | chr1:183155372-183214035 | 0.562531 | 15.0452 | 14.482669 | 4.74122 | 5.00E-05 | 0.00217928 |
| ENSG00000166670.5 | MMP10 | chr11:102641233-102651359 | 1.06253 | 15.4172 | 14.35467 | 3.85896 | 5.00E-05 | 0.00217928 |
| ENSG00000158615.8 | PPP1R15B | chr1:204372514-204380919 | 5.16099 | 19.3294 | 14.16841 | 1.90508 | 5.00E-05 | 0.00217928 |
| ENSG00000013588.5 | GPRC5A | chr12:13043715-13070871 | 6.17932 | 20.2743 | 14.09498 | 1.71413 | 5.00E-05 | 0.00217928 |
| **Gene_ID** | **Gene** | **Position** | **FPKM (Control)** | **FPKM (IL1b)** | **FPKM (Absolute Change)** | **Log2**  **(fold change)** | **p-value** | **q-value** |
| ENSG00000125657.3 | TNFSF9 | chr19:6531009-6535931 | 1.00992 | 14.9721 | 13.96218 | 3.88996 | 5.00E-05 | 0.00217928 |
| ENSG00000172216.4 | CEBPB | chr20:48807375-48809212 | 3.90203 | 17.7826 | 13.88057 | 2.18817 | 5.00E-05 | 0.00217928 |
| ENSG00000119508.13 | NR4A3 | chr9:102584136-102629173 | 2.04373 | 15.6289 | 13.58517 | 2.93494 | 5.00E-05 | 0.00217928 |
| ENSG00000059728.6 | MXD1 | chr2:70120691-70170077 | 3.72225 | 17.1314 | 13.40915 | 2.20239 | 0.0009 | 0.0265761 |
| ENSG00000178726.6 | THBD | chr20:23026269-23030378 | 13.5071 | 26.8521 | 13.345 | 0.991321 | 0.0011 | 0.0311104 |
| ENSG00000056736.5 | IL17RB | chr3:53880606-53899827 | 2.02974 | 15.316 | 13.28626 | 2.91567 | 5.00E-05 | 0.00217928 |
| ENSG00000166592.7 | RRAD | chr16:66955581-66959547 | 1.5518 | 14.1664 | 12.6146 | 3.19046 | 5.00E-05 | 0.00217928 |
| ENSG00000168389.13 | MFSD2A | chr1:40420801-40435638 | 0.619236 | 13.0631 | 12.443864 | 4.39887 | 5.00E-05 | 0.00217928 |
| ENSG00000101311.11 | FERMT1 | chr20:6055491-6104191 | 5.01048 | 16.877 | 11.86652 | 1.75204 | 0.00035 | 0.0119103 |
| ENSG00000132510.6 | KDM6B | chr17:7743221-7758114 | 0.528389 | 12.3504 | 11.822011 | 4.54681 | 5.00E-05 | 0.00217928 |
| ENSG00000176597.7 | B3GNT5 | chr3:182895830-183146566 | 3.28303 | 15.0686 | 11.78557 | 2.19845 | 5.00E-05 | 0.00217928 |
| ENSG00000171488.10 | LRRC8C | chr1:90098630-90402170 | 2.88928 | 14.4779 | 11.58862 | 2.32507 | 0.00025 | 0.0089375 |
| ENSG00000111859.12 | NEDD9 | chr6:11183530-11382581 | 3.98582 | 15.301 | 11.31518 | 1.94068 | 5.00E-05 | 0.00217928 |
| ENSG00000162783.8 | IER5 | chr1:181057637-181059977 | 4.71052 | 15.8017 | 11.09118 | 1.74612 | 5.00E-05 | 0.00217928 |
| **Gene_ID** | **Gene** | **Position** | **FPKM (Control)** | **FPKM (IL1b)** | **FPKM (Absolute Change)** | **Log2**  **(fold change)** | **p-value** | **q-value** |
| ENSG00000110218.4 | PANX1 | chr11:93862093-93915138 | 9.13987 | 19.8113 | 10.67143 | 1.11608 | 0.00065 | 0.020177 |
| ENSG00000164181.9 | ELOVL7 | chr5:60047617-60140216 | 0.126607 | 10.7118 | 10.585193 | 6.4027 | 5.00E-05 | 0.00217928 |
| ENSG00000159167.7 | STC1 | chr8:23699427-23712320 | 9.70152 | 20.1552 | 10.45368 | 1.05487 | 0.0002 | 0.00739942 |
| ENSG00000070404.5 | FSTL3 | chr19:676391-683385 | 3.2474 | 13.6082 | 10.3608 | 2.06712 | 5.00E-05 | 0.00217928 |
| ENSG00000124875.5 | CXCL6 | chr4:74702213-74714781 | 1.98789 | 12.2268 | 10.23891 | 2.62074 | 5.00E-05 | 0.00217928 |
| ENSG00000175183.5 | CSRP2 | chr12:77252494-77272840 | 1.74724 | 11.6109 | 9.86366 | 2.73234 | 5.00E-05 | 0.00217928 |
| ENSG00000122035.6 | RASL11A | chr13:27844463-27847827 | 2.61635 | 12.3232 | 9.70685 | 2.23575 | 5.00E-05 | 0.00217928 |
| ENSG00000136653.15 | RASSF5 | chr1:206680878-206785904 | 0.340058 | 9.90356 | 9.563502 | 4.86409 | 0.00145 | 0.0391808 |
| ENSG00000205364.3 | MT1M | chr16:56662970-56667898 | 4.46135 | 14.0124 | 9.55105 | 1.65115 | 5.00E-05 | 0.00217928 |
| ENSG00000171206.9 | TRIM8 | chr10:104404252-104418164 | 10.4937 | 19.9897 | 9.496 | 0.92973 | 0.00035 | 0.0119103 |
| ENSG00000135604.9 | STX11 | chr6:144471662-144509507 | 1.45819 | 10.9493 | 9.49111 | 2.90858 | 5.00E-05 | 0.00217928 |
| ENSG00000259571.1 | BLID | chr11:121986061-121986923 | 10.7097 | 20.1281 | 9.4184 | 0.910294 | 0.0019 | 0.0486545 |
| ENSG00000129521.9 | EGLN3 | chr14:34393436-34931980 | 1.2703 | 10.6206 | 9.3503 | 3.06364 | 0.0003 | 0.0104605 |
| ENSG00000179862.5 | CITED4 | chr1:41326728-41328018 | 0.805989 | 10.094 | 9.288011 | 3.64659 | 5.00E-05 | 0.00217928 |
| **Gene_ID** | **Gene** | **Position** | **FPKM (Control)** | **FPKM (IL1b)** | **FPKM (Absolute Change)** | **Log2**  **(fold change)** | **p-value** | **q-value** |
| ENSG00000146242.5 | TPBG | chr6:83072922-83080545 | 7.41986 | 16.6905 | 9.27064 | 1.16957 | 5.00E-05 | 0.00217928 |
| ENSG00000117479.8 | SLC19A2 | chr1:169433146-169455241 | 5.11183 | 14.0223 | 8.91047 | 1.45581 | 5.00E-05 | 0.00217928 |
| ENSG00000157214.9 | STEAP2 | chr7:89796903-89867451 | 3.87132 | 12.5546 | 8.68328 | 1.69731 | 0.0006 | 0.0189484 |
| ENSG00000162772.12 | ATF3 | chr1:212738675-212794119 | 2.82732 | 11.5007 | 8.67338 | 2.02421 | 5.00E-05 | 0.00217928 |
| ENSG00000145780.6 | FEM1C | chr5:114856607-114880591 | 8.00593 | 16.6479 | 8.64197 | 1.0562 | 5.00E-05 | 0.00217928 |
| ENSG00000198075.5 | SULT1C4 | chr2:108994366-109004513 | 1.33274 | 9.95062 | 8.61788 | 2.90039 | 5.00E-05 | 0.00217928 |
| ENSG00000104856.9 | RELB | chr19:45504687-45541452 | 2.17755 | 10.204 | 8.02645 | 2.22835 | 5.00E-05 | 0.00217928 |
| ENSG00000165997.4 | ARL5B | chr10:18948333-18970568 | 3.82683 | 11.8477 | 8.02087 | 1.63039 | 5.00E-05 | 0.00217928 |
| ENSG00000144655.10 | CSRNP1 | chr3:39183345-39196053 | 1.74474 | 9.67379 | 7.92905 | 2.47107 | 5.00E-05 | 0.00217928 |
| ENSG00000148346.7 | LCN2 | chr9:130911349-130915734 | 2.1361 | 9.97245 | 7.83635 | 2.22297 | 5.00E-05 | 0.00217928 |
| ENSG00000143751.9 | SDE2 | chr1:226170402-226187032 | 8.5406 | 16.2196 | 7.679 | 0.925328 | 0.0004 | 0.0133617 |
| ENSG00000184898.6 | RBM43 | chr2:152104453-152118393 | 2.53877 | 10.1656 | 7.62683 | 2.0015 | 5.00E-05 | 0.00217928 |
| ENSG00000103196.7 | CRISPLD2 | chr16:84853589-84954374 | 2.49413 | 10.1066 | 7.61247 | 2.01868 | 0.0008 | 0.024127 |
| ENSG00000120738.7 | EGR1 | chr5:137801178-137805004 | 3.85919 | 11.381 | 7.52181 | 1.56026 | 5.00E-05 | 0.00217928 |
| **Gene_ID** | **Gene** | **Position** | **FPKM (Control)** | **FPKM (IL1b)** | **FPKM (Absolute Change)** | **Log2**  **(fold change)** | **p-value** | **q-value** |
| ENSG00000179388.8 | EGR3 | chr8:22545171-22550815 | 0.809972 | 8.15784 | 7.347868 | 3.33224 | 5.00E-05 | 0.00217928 |
| ENSG00000113504.15 | SLC12A7 | chr5:1050498-1112150 | 1.94208 | 9.19542 | 7.25334 | 2.24332 | 5.00E-05 | 0.00217928 |
| ENSG00000137802.9 | MAPKBP1 | chr15:42066631-42120053 | 1.96332 | 9.11864 | 7.15532 | 2.21552 | 5.00E-05 | 0.00217928 |
| ENSG00000167378.4 | IRGQ | chr19:44047191-44100287 | 3.77276 | 10.8148 | 7.04204 | 1.51932 | 0.0003 | 0.0104605 |
| ENSG00000143157.7 | POGK | chr1:166808680-166825581 | 6.88355 | 13.7907 | 6.90715 | 1.00247 | 0.0015 | 0.040022 |
| ENSG00000128016.4 | ZFP36 | chr19:39897452-39900052 | 2.37424 | 9.22501 | 6.85077 | 1.95809 | 5.00E-05 | 0.00217928 |
| ENSG00000175040.4 | CHST2 | chr3:142838172-142841800 | 6.20135 | 12.9974 | 6.79605 | 1.06756 | 5.00E-05 | 0.00217928 |
| ENSG00000086544.2 | ITPKC | chr19:41197433-41271294 | 4.0982 | 10.8278 | 6.7296 | 1.40168 | 0.0012 | 0.0336396 |
| ENSG00000100647.7 | KIAA0247 | chr14:70078312-70181859 | 3.13321 | 9.83136 | 6.69815 | 1.64975 | 0.0003 | 0.0104605 |
| ENSG00000170542.5 | SERPINB9 | chr6:2887499-2903514 | 7.08283 | 13.6637 | 6.58087 | 0.947955 | 0.00015 | 0.00576752 |
| ENSG00000185112.4 | FAM43A | chr3:194406621-194409762 | 3.99175 | 10.2053 | 6.21355 | 1.35422 | 5.00E-05 | 0.00217928 |
| ENSG00000172315.5 | TP53RK | chr20:45313003-45318418 | 5.40044 | 11.6137 | 6.21326 | 1.10468 | 0.00035 | 0.0119103 |
| ENSG00000006075.11 | CCL3 | chr17:34415601-34417515 | 0 | 6.10074 | 6.10074 | inf | 5.00E-05 | 0.00217928 |
| ENSG00000101017.9 | CD40 | chr20:44746910-44758502 | 2.86385 | 8.8304 | 5.96655 | 1.62452 | 0.0003 | 0.0104605 |
| **Gene_ID** | **Gene** | **Position** | **FPKM (Control)** | **FPKM (IL1b)** | **FPKM (Absolute Change)** | **Log2**  **(fold change)** | **p-value** | **q-value** |
| ENSG00000168209.4 | DDIT4 | chr10:74033677-74035794 | 2.04306 | 7.98899 | 5.94593 | 1.96728 | 5.00E-05 | 0.00217928 |
| ENSG00000115267.5 | IFIH1 | chr2:163123588-163175213 | 3.25915 | 9.18528 | 5.92613 | 1.49483 | 0.00145 | 0.0391808 |
| ENSG00000095752.2 | IL11 | chr19:55875756-55881831 | 0.918602 | 6.77629 | 5.857688 | 2.88298 | 5.00E-05 | 0.00217928 |
| ENSG00000006128.7 | TAC1 | chr7:97361219-97369784 | 3.31605 | 9.12015 | 5.8041 | 1.45959 | 0.0005 | 0.0163167 |
| ENSG00000162413.12 | KLHL21 | chr1:6650783-6684093 | 6.13305 | 11.9366 | 5.80355 | 0.960717 | 0.0018 | 0.04628 |
| ENSG00000213694.3 | S1PR3 | chr9:91605777-91619925 | 3.10221 | 8.85927 | 5.75706 | 1.51389 | 5.00E-05 | 0.00217928 |
| ENSG00000136634.5 | IL10 | chr1:206940946-206945839 | 0.0799456 | 5.82247 | 5.7425244 | 6.18647 | 0.00015 | 0.00576752 |
| ENSG00000015475.14 | BID | chr22:18216905-18257536 | 2.44932 | 8.10236 | 5.65304 | 1.72596 | 0.0015 | 0.040022 |
| ENSG00000137267.5 | TUBB2A | chr6:3153902-3157760 | 3.93265 | 9.58106 | 5.64841 | 1.28468 | 0.0002 | 0.00739942 |
| ENSG00000134470.15 | IL15RA | chr10:5990854-6020150 | 2.36968 | 7.92928 | 5.5596 | 1.7425 | 0.00115 | 0.0323806 |
| ENSG00000179826.5 | MRGPRX3 | chr11:18142501-18160027 | 0.0435602 | 5.60236 | 5.5587998 | 7.00688 | 5.00E-05 | 0.00217928 |
| ENSG00000156966.6 | B3GNT7 | chr2:232260253-232265875 | 4.98979 | 10.4941 | 5.50431 | 1.07252 | 5.00E-05 | 0.00217928 |
| ENSG00000119714.6 | GPR68 | chr14:91698875-91720269 | 4.23212 | 9.71549 | 5.48337 | 1.19891 | 0.00065 | 0.020177 |
| ENSG00000255874.1 | LINC00346 | chr13:111521577-111522162 | 1.62589 | 6.98229 | 5.3564 | 2.10247 | 0.00015 | 0.00576752 |
| **Gene_ID** | **Gene** | **Position** | **FPKM (Control)** | **FPKM (IL1b)** | **FPKM (Absolute Change)** | **Log2**  **(fold change)** | **p-value** | **q-value** |
| ENSG00000204103.2 | MAFB | chr20:39314487-39317880 | 2.09294 | 7.26521 | 5.17227 | 1.79548 | 5.00E-05 | 0.00217928 |
| ENSG00000144824.15 | PHLDB2 | chr3:111393522-111695364 | 2.03092 | 7.19183 | 5.16091 | 1.82423 | 0.00045 | 0.0148757 |
| ENSG00000122420.5 | PTGFR | chr1:78769567-79005434 | 2.52908 | 7.4723 | 4.94322 | 1.56294 | 0.00075 | 0.0228903 |
| ENSG00000128271.15 | ADORA2A | chr22:24666785-24838328 | 0.0325229 | 4.83926 | 4.8067371 | 7.21718 | 0.00045 | 0.0148757 |
| ENSG00000167604.9 | NFKBID | chr19:36378554-36393205 | 0.8779 | 5.54618 | 4.66828 | 2.65937 | 5.00E-05 | 0.00217928 |
| ENSG00000120337.7 | TNFSF18 | chr1:173009099-173020103 | 0.148297 | 4.64982 | 4.501523 | 4.97061 | 5.00E-05 | 0.00217928 |
| ENSG00000164920.5 | OSR2 | chr8:99956630-99964332 | 2.48741 | 6.95429 | 4.46688 | 1.48326 | 5.00E-05 | 0.00217928 |
| ENSG00000115604.6 | IL18R1 | chr2:102927961-103015218 | 0.948259 | 5.36322 | 4.414961 | 2.49975 | 0.0009 | 0.0265761 |
| ENSG00000109158.6 | GABRA4 | chr4:46920916-47428461 | 0.827623 | 5.23327 | 4.405647 | 2.66067 | 0.0013 | 0.0359676 |
| ENSG00000183421.7 | RIPK4 | chr21:43159528-43187266 | 1.18866 | 5.54243 | 4.35377 | 2.22118 | 5.00E-05 | 0.00217928 |
| ENSG00000070193.4 | FGF10 | chr5:44303645-44389808 | 0.518197 | 4.87099 | 4.352793 | 3.23264 | 5.00E-05 | 0.00217928 |
| ENSG00000125726.6 | CD70 | chr19:6583193-6604114 | 0.33475 | 4.61172 | 4.27697 | 3.78415 | 0.00025 | 0.0089375 |
| ENSG00000142627.9 | EPHA2 | chr1:16450831-16482582 | 2.72066 | 6.90097 | 4.18031 | 1.34284 | 0.00195 | 0.0497348 |
| ENSG00000169242.7 | EFNA1 | chr1:155099935-155107333 | 1.30822 | 5.40639 | 4.09817 | 2.04707 | 5.00E-05 | 0.00217928 |
| **Gene_ID** | **Gene** | **Position** | **FPKM (Control)** | **FPKM (IL1b)** | **FPKM (Absolute Change)** | **Log2**  **(fold change)** | **p-value** | **q-value** |
| ENSG00000105327.11 | BBC3 | chr19:47724080-47736023 | 2.38673 | 6.45726 | 4.07053 | 1.43589 | 0.00035 | 0.0119103 |
| ENSG00000128710.5 | HOXD10 | chr2:176968943-176984670 | 1.39922 | 5.42724 | 4.02802 | 1.9556 | 0.0001 | 0.00409228 |
| ENSG00000125430.4 | HS3ST3B1 | chr17:14204399-14252721 | 0.974368 | 4.82784 | 3.853472 | 2.30884 | 5.00E-05 | 0.00217928 |
| ENSG00000121858.6 | TNFSF10 | chr3:172223297-172241297 | 3.28268 | 7.08973 | 3.80705 | 1.11086 | 0.00165 | 0.0430319 |
| ENSG00000049249.4 | TNFRSF9 | chr1:7979906-8000926 | 0.186705 | 3.95983 | 3.773125 | 4.40661 | 5.00E-05 | 0.00217928 |
| ENSG00000163735.6 | CXCL5 | chr4:74861358-74864496 | 0.107587 | 3.87959 | 3.772003 | 5.17232 | 5.00E-05 | 0.00217928 |
| ENSG00000108551.4 | RASD1 | chr17:17397750-17399709 | 1.08337 | 4.80545 | 3.72208 | 2.14915 | 5.00E-05 | 0.00217928 |
| ENSG00000103257.4 | SLC7A5 | chr16:87863628-87903094 | 2.12259 | 5.80429 | 3.6817 | 1.45129 | 5.00E-05 | 0.00217928 |
| ENSG00000121797.9 | CCRL2 | chr3:46448653-46454488 | 0.939317 | 4.56522 | 3.625903 | 2.281 | 5.00E-05 | 0.00217928 |
| ENSG00000143067.4 | ZNF697 | chr1:120162044-120190396 | 1.78027 | 5.36537 | 3.5851 | 1.59158 | 5.00E-05 | 0.00217928 |
| ENSG00000105371.8 | ICAM4 | chr19:10397642-10399198 | 0.125049 | 3.70062 | 3.575571 | 4.88721 | 5.00E-05 | 0.00217928 |
| ENSG00000188211.4 | NCR3LG1 | chr11:17373272-17398888 | 2.72468 | 6.24492 | 3.52024 | 1.19659 | 0.00015 | 0.00576752 |
| ENSG00000198535.5 | C2CD4A | chr15:62359175-62363116 | 0.145923 | 3.43224 | 3.286317 | 4.55587 | 5.00E-05 | 0.00217928 |
| ENSG00000181649.5 | PHLDA2 | chr11:2949502-2950685 | 2.24376 | 5.457 | 3.21324 | 1.28219 | 0.00145 | 0.0391808 |
| **Gene_ID** | **Gene** | **Position** | **FPKM (Control)** | **FPKM (IL1b)** | **FPKM (Absolute Change)** | **Log2**  **(fold change)** | **p-value** | **q-value** |
| ENSG00000156427.7 | FGF18 | chr5:170846659-170884627 | 1.67134 | 4.83057 | 3.15923 | 1.53119 | 0.0001 | 0.00409228 |
| ENSG00000253276.1 | CCDC71L | chr7:106297210-106301442 | 2.61111 | 5.70532 | 3.09421 | 1.12764 | 0.0002 | 0.00739942 |
| ENSG00000163694.10 | RBM47 | chr4:40425271-40632892 | 0.311676 | 3.3851 | 3.073424 | 3.44108 | 5.00E-05 | 0.00217928 |
| ENSG00000160326.9 | SLC2A6 | chr9:136336216-136344259 | 1.61437 | 4.57781 | 2.96344 | 1.50369 | 5.00E-05 | 0.00217928 |
| ENSG00000154099.13 | DNAAF1 | chr16:84178864-84220669 | 0.136477 | 3.07778 | 2.941303 | 4.49516 | 0.0001 | 0.00409228 |
| ENSG00000167874.6 | TMEM88 | chr17:7758382-7759417 | 0.668325 | 3.60287 | 2.934545 | 2.43052 | 0.0016 | 0.0420727 |
| ENSG00000124466.8 | LYPD3 | chr19:43964938-43969812 | 0.098649 | 2.78224 | 2.683591 | 4.8178 | 5.00E-05 | 0.00217928 |
| ENSG00000136695.10 | IL36RN | chr2:113816214-113822959 | 0.016011 | 2.53181 | 2.515799 | 7.30497 | 0.00015 | 0.00576752 |
| ENSG00000263563.1 | UBBP4 | chr17:21729600-21731762 | 1.18041 | 3.69532 | 2.51491 | 1.64641 | 0.0002 | 0.00739942 |
| ENSG00000160223.12 | ICOSLG | chr21:45642873-45660849 | 0.190777 | 2.66943 | 2.478653 | 3.80657 | 5.00E-05 | 0.00217928 |
| ENSG00000183742.8 | MACC1 | chr7:20174277-20257027 | 0.309522 | 2.77879 | 2.469268 | 3.16634 | 5.00E-05 | 0.00217928 |
| ENSG00000152689.13 | RASGRP3 | chr2:33661390-33789817 | 0.695355 | 3.14984 | 2.454485 | 2.17946 | 0.00055 | 0.0176764 |
| ENSG00000050730.11 | TNIP3 | chr4:122052562-122148621 | 0.349154 | 2.797 | 2.447846 | 3.00195 | 0.00065 | 0.020177 |
| ENSG00000008517.12 | IL32 | chr16:3115297-3131908 | 0.536415 | 2.95296 | 2.416545 | 2.46074 | 5.00E-05 | 0.00217928 |
| **Gene_ID** | **Gene** | **Position** | **FPKM (Control)** | **FPKM (IL1b)** | **FPKM (Absolute Change)** | **Log2**  **(fold change)** | **p-value** | **q-value** |
| ENSG00000176928.4 | GCNT4 | chr5:74323288-74326724 | 0.42166 | 2.80714 | 2.38548 | 2.73495 | 5.00E-05 | 0.00217928 |
| ENSG00000135111.10 | TBX3 | chr12:115108058-115121969 | 0.659473 | 3.04011 | 2.380637 | 2.20474 | 5.00E-05 | 0.00217928 |
| ENSG00000167207.7 | NOD2 | chr16:50727513-50766988 | 0.125196 | 2.50066 | 2.375464 | 4.32005 | 5.00E-05 | 0.00217928 |
| ENSG00000137265.10 | IRF4 | chr6:391738-411447 | 0.199524 | 2.51717 | 2.317646 | 3.65717 | 5.00E-05 | 0.00217928 |
| ENSG00000196155.8 | PLEKHG4 | chr16:67311412-67360666 | 1.03068 | 3.34119 | 2.31051 | 1.69677 | 0.00095 | 0.0276674 |
| ENSG00000122824.6 | NUDT10 | chrX:51075082-51080377 | 1.63945 | 3.9441 | 2.30465 | 1.26648 | 0.0007 | 0.0215191 |
| ENSG00000181634.7 | TNFSF15 | chr9:117546914-117568406 | 0.538542 | 2.72159 | 2.183048 | 2.33732 | 5.00E-05 | 0.00217928 |
| ENSG00000162654.8 | GBP4 | chr1:89646830-89664615 | 0.978248 | 3.1448 | 2.166552 | 1.6847 | 0.00095 | 0.0276674 |
| ENSG00000108700.4 | CCL8 | chr17:32646054-32648421 | 0.178593 | 2.24219 | 2.063597 | 3.65016 | 0.00045 | 0.0148757 |
| ENSG00000115956.9 | PLEK | chr2:68592304-68624585 | 0.0639822 | 2.0969 | 2.0329178 | 5.03444 | 5.00E-05 | 0.00217928 |
| ENSG00000140406.2 | MESDC1 | chr15:81293294-81296342 | 0.562279 | 2.59074 | 2.028461 | 2.20401 | 5.00E-05 | 0.00217928 |
| ENSG00000174792.6 | C4orf26 | chr4:76481257-76491095 | 0.162646 | 2.17838 | 2.015734 | 3.74345 | 0.0005 | 0.0163167 |
| ENSG00000118513.14 | MYB | chr6:135502452-135540311 | 0.102102 | 2.09604 | 1.993938 | 4.35959 | 5.00E-05 | 0.00217928 |
| ENSG00000028277.16 | POU2F2 | chr19:42590262-42700737 | 0.239565 | 1.98257 | 1.743005 | 3.04888 | 5.00E-05 | 0.00217928 |
| **Gene_ID** | **Gene** | **Position** | **FPKM (Control)** | **FPKM (IL1b)** | **FPKM (Absolute Change)** | **Log2**  **(fold change)** | **p-value** | **q-value** |
| ENSG00000180616.4 | SSTR2 | chr17:71161150-71167185 | 0.153126 | 1.85784 | 1.704714 | 3.60083 | 5.00E-05 | 0.00217928 |
| ENSG00000163082.9 | SGPP2 | chr2:223289235-223425667 | 0.0158043 | 1.67882 | 1.6630157 | 6.73098 | 5.00E-05 | 0.00217928 |
| ENSG00000189266.7 | PNRC2 | chr1:24285598-24289952 | 1.27401 | 2.90992 | 1.63591 | 1.19161 | 0.00125 | 0.0348876 |
| ENSG00000115008.5 | IL1A | chr2:113531491-113542167 | 0.0195282 | 1.6542 | 1.6346718 | 6.40443 | 0.0001 | 0.00409228 |
| ENSG00000198205.5 | ZXDA | chrX:57931863-57937067 | 0.923848 | 2.53628 | 1.612432 | 1.45699 | 5.00E-05 | 0.00217928 |
| ENSG00000170054.10 | SERPINA9 | chr14:94929053-94946026 | 0.122032 | 1.71295 | 1.590918 | 3.81114 | 5.00E-05 | 0.00217928 |
| ENSG00000138135.5 | CH25H | chr10:90965693-90967071 | 0.127694 | 1.71781 | 1.590116 | 3.74981 | 0.0015 | 0.040022 |
| ENSG00000152503.5 | TRIM36 | chr5:114460458-114516243 | 0.465357 | 2.05319 | 1.587833 | 2.14146 | 0.00025 | 0.0089375 |
| ENSG00000103710.6 | RASL12 | chr15:65337707-65369028 | 0.614981 | 2.16242 | 1.547439 | 1.81403 | 0.0004 | 0.0133617 |
| ENSG00000139438.5 | FAM222A | chr12:110152032-110208312 | 0.342376 | 1.83319 | 1.490814 | 2.4207 | 0.00105 | 0.03003 |
| ENSG00000151617.11 | EDNRA | chr4:148402068-148466106 | 0.231479 | 1.69492 | 1.463441 | 2.87227 | 0.00015 | 0.00576752 |
| ENSG00000175130.6 | MARCKSL1 | chr1:32799432-32801980 | 0.306237 | 1.70502 | 1.398783 | 2.47707 | 5.00E-05 | 0.00217928 |
| ENSG00000019186.5 | CYP24A1 | chr20:52769987-52790512 | 0.0671135 | 1.42447 | 1.3573565 | 4.40768 | 0.00025 | 0.0089375 |
| ENSG00000197632.4 | SERPINB2 | chr18:61538925-61609601 | 0.0623758 | 1.4087 | 1.3463242 | 4.49723 | 0.00025 | 0.0089375 |
| **Gene_ID** | **Gene** | **Position** | **FPKM (Control)** | **FPKM (IL1b)** | **FPKM (Absolute Change)** | **Log2**  **(fold change)** | **p-value** | **q-value** |
| ENSG00000112499.8 | SLC22A2 | chr6:160592092-160698670 | 0.0474401 | 1.38944 | 1.3419999 | 4.87225 | 5.00E-05 | 0.00217928 |
| ENSG00000142178.7 | SIK1 | chr21:44834394-44847008 | 0.300956 | 1.64175 | 1.340794 | 2.44761 | 5.00E-05 | 0.00217928 |
| ENSG00000154548.8 | SRSF12 | chr6:89805677-89827800 | 0.543177 | 1.87785 | 1.334673 | 1.78958 | 0.00155 | 0.0410976 |
| ENSG00000214900.4 | C14orf182 | chr14:50448429-50474238 | 0.464977 | 1.76235 | 1.297373 | 1.92227 | 0.0008 | 0.024127 |
| ENSG00000188931.3 | C1orf192 | chr1:161334520-161337664 | 0.218767 | 1.50968 | 1.290913 | 2.78678 | 5.00E-05 | 0.00217928 |
| ENSG00000168334.8 | XIRP1 | chr3:39224700-39234087 | 0.0592646 | 1.33391 | 1.2746454 | 4.49235 | 5.00E-05 | 0.00217928 |
| ENSG00000154914.12 | USP43 | chr17:9548014-9633008 | 0.0755333 | 1.34158 | 1.2660467 | 4.15068 | 5.00E-05 | 0.00217928 |
| ENSG00000057657.10 | PRDM1 | chr6:106534194-106557814 | 0.183455 | 1.34868 | 1.165225 | 2.87805 | 0.0002 | 0.00739942 |
| ENSG00000158869.6 | FCER1G | chr1:161185023-161193421 | 0.0206513 | 1.16571 | 1.1450587 | 5.81884 | 0.00025 | 0.0089375 |
| ENSG00000142224.11 | IL19 | chr1:206972214-207016324 | 0.0641104 | 1.20428 | 1.1401696 | 4.23146 | 5.00E-05 | 0.00217928 |
| ENSG00000258691.1 | RP11-404P21.8 | chr14:96671015-96735304 | 0.115842 | 1.18177 | 1.065928 | 3.35072 | 5.00E-05 | 0.00217928 |
| ENSG00000187764.7 | SEMA4D | chr9:91975701-92113045 | 0.264733 | 1.31442 | 1.049687 | 2.31182 | 0.001 | 0.0289909 |
| ENSG00000186407.4 | CD300E | chr17:72606025-72619897 | 0.0474478 | 1.09321 | 1.0457622 | 4.52608 | 5.00E-05 | 0.00217928 |
| ENSG00000175445.10 | LPL | chr8:19759227-19824769 | 0.173972 | 1.19947 | 1.025498 | 2.78548 | 0.00035 | 0.0119103 |
| **Gene_ID** | **Gene** | **Position** | **FPKM (Control)** | **FPKM (IL1b)** | **FPKM (Absolute Change)** | **Log2**  **(fold change)** | **p-value** | **q-value** |
| ENSG00000181656.6 | GPR88 | chr1:101003692-101007574 | 0.717266 | 1.7362 | 1.018934 | 1.27536 | 0.0003 | 0.0104605 |
| ENSG00000128052.8 | KDR | chr4:55944643-55991756 | 0.221827 | 1.21711 | 0.995283 | 2.45595 | 5.00E-05 | 0.00217928 |
| ENSG00000126545.9 | CSN1S1 | chr4:70796798-70812289 | 0.0129425 | 0.993959 | 0.9810165 | 6.26299 | 5.00E-05 | 0.00217928 |
| ENSG00000166396.8 | SERPINB7 | chr18:61420168-61472604 | 0.101541 | 1.05949 | 0.957949 | 3.38323 | 0.0004 | 0.0133617 |
| ENSG00000176678.4 | FOXL1 | chr16:86609973-86615303 | 0.774657 | 1.71949 | 0.944833 | 1.15035 | 0.00125 | 0.0348876 |
| ENSG00000153094.17 | BCL2L11 | chr2:111876954-111926024 | 0.320984 | 1.23132 | 0.910336 | 1.93963 | 0.00115 | 0.0323806 |
| ENSG00000227471.4 | AKR1B15 | chr7:134233887-134264627 | 0.0568899 | 0.95089 | 0.8940001 | 4.06303 | 0.0002 | 0.00739942 |
| ENSG00000100678.14 | SLC8A3 | chr14:70510933-70655787 | 0.17916 | 1.06672 | 0.88756 | 2.57386 | 0.0002 | 0.00739942 |
| ENSG00000150510.11 | FAM124A | chr13:51796502-51858377 | 0.365225 | 1.25202 | 0.886795 | 1.7774 | 0.0009 | 0.0265761 |
| ENSG00000133101.5 | CCNA1 | chr13:37005966-37017019 | 0.0704446 | 0.853211 | 0.7827664 | 3.59834 | 0.0006 | 0.0189484 |
| ENSG00000162711.12 | NLRP3 | chr1:247579457-247612410 | 0.0424302 | 0.805 | 0.7625698 | 4.24583 | 5.00E-05 | 0.00217928 |
| ENSG00000177627.5 | C12orf54 | chr12:48876285-48890295 | 0.0144042 | 0.740078 | 0.7256738 | 5.68312 | 5.00E-05 | 0.00217928 |
| ENSG00000163545.7 | NUAK2 | chr1:205271186-205290883 | 0.0629175 | 0.773365 | 0.7104475 | 3.61962 | 5.00E-05 | 0.00217928 |
| ENSG00000126003.6 | PLAGL2 | chr20:30780305-30795594 | 0.494571 | 1.17777 | 0.683199 | 1.2518 | 0.0013 | 0.0359676 |
| **Gene_ID** | **Gene** | **Position** | **FPKM (Control)** | **FPKM (IL1b)** | **FPKM (Absolute Change)** | **Log2**  **(fold change)** | **p-value** | **q-value** |
| ENSG00000172575.7 | RASGRP1 | chr15:38780303-38857776 | 0.14968 | 0.828665 | 0.678985 | 2.46891 | 0.00015 | 0.00576752 |
| ENSG00000005102.8 | MEOX1 | chr17:41717755-41739322 | 0.0679865 | 0.658875 | 0.5908885 | 3.27669 | 0.0018 | 0.04628 |
| ENSG00000242715.3 | CCDC169 | chr13:36742344-36871979 | 0.128799 | 0.695393 | 0.566594 | 2.43271 | 0.0006 | 0.0189484 |
| ENSG00000137648.12 | TMPRSS4 | chr11:117947752-117992605 | 0.10612 | 0.639955 | 0.533835 | 2.59228 | 0.0001 | 0.00409228 |
| ENSG00000099338.18 | CATSPERG | chr19:38826414-38861589 | 0.0733935 | 0.574468 | 0.5010745 | 2.9685 | 5.00E-05 | 0.00217928 |
| ENSG00000145832.8 | SLC25A48 | chr5:135170337-135224326 | 0.018461 | 0.499139 | 0.480678 | 4.75688 | 0.00065 | 0.020177 |
| ENSG00000243566.2 | UPK3B | chr7:76139744-76648340 | 0.384607 | 0.862389 | 0.477782 | 1.16495 | 5.00E-05 | 0.00217928 |
| ENSG00000229314.4 | ORM1 | chr9:117085335-117088755 | 0 | 0.477272 | 0.477272 | inf | 5.00E-05 | 0.00217928 |
| ENSG00000204365.2 | C10orf126 | chr10:29135336-29170827 | 0.00430331 | 0.481414 | 0.47711069 | 6.80569 | 0.00055 | 0.0176764 |
| ENSG00000185966.3 | LCE3E | chr1:152538129-152539248 | 0 | 0.472331 | 0.472331 | inf | 5.00E-05 | 0.00217928 |
| ENSG00000140795.8 | MYLK3 | chr16:46740890-46824319 | 0.0403633 | 0.502567 | 0.4622037 | 3.6382 | 0.00055 | 0.0176764 |
| ENSG00000161835.6 | GRASP | chr12:52400723-52409673 | 0.109575 | 0.56853 | 0.458955 | 2.37532 | 0.0014 | 0.0380722 |
| ENSG00000137571.6 | SLCO5A1 | chr8:70579281-70747299 | 0.0214849 | 0.479958 | 0.4584731 | 4.48151 | 5.00E-05 | 0.00217928 |
| ENSG00000093134.9 | VNN3 | chr6:133043925-133055904 | 0.0612107 | 0.514962 | 0.4537513 | 3.07261 | 5.00E-05 | 0.00217928 |
| **Gene_ID** | **Gene** | **Position** | **FPKM (Control)** | **FPKM (IL1b)** | **FPKM (Absolute Change)** | **Log2**  **(fold change)** | **p-value** | **q-value** |
| ENSG00000187944.2 | C2orf66 | chr2:197669725-197675000 | 0.10545 | 0.527738 | 0.422288 | 2.32326 | 0.0014 | 0.0380722 |
| ENSG00000143452.11 | HORMAD1 | chr1:150670535-150693364 | 0.0449533 | 0.457451 | 0.4124977 | 3.34712 | 0.00045 | 0.0148757 |
| ENSG00000145911.5 | N4BP3 | chr5:177540443-177553088 | 0.0529067 | 0.437279 | 0.3843723 | 3.04703 | 5.00E-05 | 0.00217928 |
| ENSG00000010379.11 | SLC6A13 | chr12:329788-372039 | 0.0532165 | 0.421226 | 0.3680095 | 2.98465 | 0.0001 | 0.00409228 |
| ENSG00000114739.9 | ACVR2B | chr3:38495341-38534633 | 0.581393 | 0.209785 | -0.371608 | -1.4706 | 0.0001 | 0.00409228 |
| ENSG00000177679.14 | SRRM3 | chr7:75831215-75916605 | 0.406601 | 0.0235593 | -0.3830417 | -4.10924 | 0.00085 | 0.025454 |
| ENSG00000215853.3 | RPTN | chr1:152126070-152131704 | 0.645621 | 0.203566 | -0.442055 | -1.66519 | 0.0015 | 0.040022 |
| ENSG00000133247.9 | SUV420H2 | chr19:55851220-55859488 | 0.534985 | 0.0600286 | -0.4749564 | -3.15578 | 0.0004 | 0.0133617 |
| ENSG00000105997.18 | HOXA3 | chr7:27145802-27192200 | 0.651449 | 0.168271 | -0.483178 | -1.95286 | 0.00015 | 0.00576752 |
| ENSG00000058404.15 | CAMK2B | chr7:44256748-44374176 | 0.614582 | 0.107574 | -0.507008 | -2.51427 | 0.0001 | 0.00409228 |
| ENSG00000074370.13 | ATP2A3 | chr17:3827168-3867736 | 0.583442 | 0.0669394 | -0.5165026 | -3.12366 | 5.00E-05 | 0.00217928 |
| ENSG00000014257.11 | ACPP | chr3:132036210-132087142 | 0.566731 | 0.0467128 | -0.5200182 | -3.60077 | 0.00015 | 0.00576752 |
| ENSG00000179930.5 | ZNF648 | chr1:182023704-182030847 | 0.766794 | 0.244027 | -0.522767 | -1.6518 | 0.00055 | 0.0176764 |
| ENSG00000185730.3 | ZNF696 | chr8:144371845-144380231 | 0.696774 | 0.16297 | -0.533804 | -2.09609 | 0.0002 | 0.00739942 |
| **Gene_ID** | **Gene** | **Position** | **FPKM (Control)** | **FPKM (IL1b)** | **FPKM (Absolute Change)** | **Log2**  **(fold change)** | **p-value** | **q-value** |
| ENSG00000180884.9 | ZNF792 | chr19:35447257-35454953 | 0.786863 | 0.2407 | -0.546163 | -1.70887 | 0.0013 | 0.0359676 |
| ENSG00000111679.12 | PTPN6 | chr12:7055630-7070479 | 0.929769 | 0.344824 | -0.584945 | -1.43101 | 0.00175 | 0.045177 |
| ENSG00000213316.5 | LTC4S | chr5:179159850-179223648 | 0.607404 | 0 | -0.607404 | #NAME? | 0.00015 | 0.00576752 |
| ENSG00000182749.5 | PAQR7 | chr1:26187700-26197744 | 1.07841 | 0.387479 | -0.690931 | -1.47672 | 0.0011 | 0.0311104 |
| ENSG00000115112.7 | TFCP2L1 | chr2:121974162-122042783 | 1.13168 | 0.39592 | -0.73576 | -1.51519 | 0.0006 | 0.0189484 |
| ENSG00000204446.2 | C9orf170 | chr9:89763558-89774471 | 0.914133 | 0.170603 | -0.74353 | -2.42176 | 5.00E-05 | 0.00217928 |
| ENSG00000160051.7 | IQCC | chr1:32671235-32674288 | 1.10279 | 0.323637 | -0.779153 | -1.76871 | 0.00095 | 0.0276674 |
| ENSG00000185875.8 | THNSL1 | chr10:25305586-25315593 | 1.31175 | 0.479057 | -0.832693 | -1.45323 | 0.0007 | 0.0215191 |
| ENSG00000180346.2 | TIGD2 | chr4:90033967-90036050 | 1.31097 | 0.46798 | -0.84299 | -1.48611 | 0.00165 | 0.0430319 |
| ENSG00000130775.11 | THEMIS2 | chr1:28199054-28213196 | 1.28631 | 0.430449 | -0.855861 | -1.57933 | 0.00135 | 0.037109 |
| ENSG00000183508.4 | FAM46C | chr1:118148555-118170994 | 1.43886 | 0.570053 | -0.868807 | -1.33576 | 0.0002 | 0.00739942 |
| ENSG00000164976.8 | KIAA1161 | chr9:34366667-34376851 | 1.7263 | 0.823342 | -0.902958 | -1.06812 | 0.0006 | 0.0189484 |
| ENSG00000129757.8 | CDKN1C | chr11:2904442-2907111 | 1.43279 | 0.470013 | -0.962777 | -1.60806 | 0.0017 | 0.044065 |
| ENSG00000136002.12 | ARHGEF4 | chr2:131594488-131804836 | 1.37071 | 0.333448 | -1.037262 | -2.03939 | 0.0011 | 0.0311104 |
| **Gene_ID** | **Gene** | **Position** | **FPKM (Control)** | **FPKM (IL1b)** | **FPKM (Absolute Change)** | **Log2**  **(fold change)** | **p-value** | **q-value** |
| ENSG00000168612.4 | ZSWIM1 | chr20:44509865-44513905 | 2.12873 | 0.946395 | -1.182335 | -1.16948 | 0.0019 | 0.0486545 |
| ENSG00000004660.10 | CAMKK1 | chr17:3763608-3798185 | 1.63002 | 0.421209 | -1.208811 | -1.95228 | 0.00015 | 0.00576752 |
| ENSG00000104081.9 | BMF | chr15:40380090-40401093 | 1.60451 | 0.350331 | -1.254179 | -2.19534 | 0.00015 | 0.00576752 |
| ENSG00000165572.6 | KBTBD6 | chr13:41701704-41706882 | 2.10083 | 0.839822 | -1.261008 | -1.32281 | 0.0001 | 0.00409228 |
| ENSG00000120696.8 | KBTBD7 | chr13:41763968-41768702 | 2.16761 | 0.873363 | -1.294247 | -1.31145 | 0.0001 | 0.00409228 |
| ENSG00000097096.8 | SYDE2 | chr1:85622555-85666729 | 2.09734 | 0.80075 | -1.29659 | -1.38914 | 0.0006 | 0.0189484 |
| ENSG00000141569.6 | TRIM65 | chr17:73876415-73893084 | 1.87053 | 0.540331 | -1.330199 | -1.79153 | 0.0004 | 0.0133617 |
| ENSG00000106031.6 | HOXA13 | chr7:27233121-27239725 | 1.74554 | 0.388687 | -1.356853 | -2.16699 | 0.0002 | 0.00739942 |
| ENSG00000176595.3 | KBTBD11 | chr8:1922043-1955102 | 2.73456 | 1.00048 | -1.73408 | -1.45062 | 5.00E-05 | 0.00217928 |
| ENSG00000145945.5 | FAM50B | chr6:3849619-3851551 | 2.52552 | 0.777883 | -1.747637 | -1.69895 | 0.0001 | 0.00409228 |
| ENSG00000168061.9 | SAC3D1 | chr11:64808372-64826021 | 2.40133 | 0.604398 | -1.796932 | -1.99026 | 0.0004 | 0.0133617 |
| ENSG00000159208.11 | C1orf51 | chr1:150254952-150259505 | 2.63326 | 0.72321 | -1.91005 | -1.86436 | 0.00095 | 0.0276674 |
| ENSG00000149050.5 | ZNF214 | chr11:7020548-7041599 | 2.58478 | 0.612675 | -1.972105 | -2.07685 | 0.00175 | 0.045177 |
| ENSG00000119669.3 | IRF2BPL | chr14:77490887-77495034 | 4.23657 | 2.11952 | -2.11705 | -0.999159 | 0.0006 | 0.0189484 |
| **Gene_ID** | **Gene** | **Position** | **FPKM (Control)** | **FPKM (IL1b)** | **FPKM (Absolute Change)** | **Log2**  **(fold change)** | **p-value** | **q-value** |
| ENSG00000165617.10 | DACT1 | chr14:59100684-59115039 | 3.25766 | 1.11326 | -2.1444 | -1.54904 | 5.00E-05 | 0.00217928 |
| ENSG00000109705.7 | NKX3-2 | chr4:13542453-13546674 | 2.65418 | 0.500394 | -2.153786 | -2.40713 | 5.00E-05 | 0.00217928 |
| ENSG00000170577.7 | SIX2 | chr2:45232299-45236569 | 3.49919 | 1.30863 | -2.19056 | -1.41897 | 0.0002 | 0.00739942 |
| ENSG00000164086.8 | DUSP7 | chr3:52082934-52090566 | 3.85821 | 1.49523 | -2.36298 | -1.36757 | 5.00E-05 | 0.00217928 |
| ENSG00000005073.5 | HOXA11 | chr7:27221128-27224842 | 2.96936 | 0.594019 | -2.375341 | -2.32157 | 5.00E-05 | 0.00217928 |
| ENSG00000188483.6 | IER5L | chr9:131937834-131940540 | 3.65722 | 1.05426 | -2.60296 | -1.79452 | 5.00E-05 | 0.00217928 |
| ENSG00000130844.12 | ZNF331 | chr19:54024234-54083523 | 4.64317 | 1.85066 | -2.79251 | -1.32707 | 0.00105 | 0.03003 |
| ENSG00000180917.12 | CMTR2 | chr16:71315291-71323618 | 5.87018 | 3.00834 | -2.86184 | -0.964438 | 0.0011 | 0.0311104 |
| ENSG00000005513.9 | SOX8 | chr16:1031807-1036979 | 4.71718 | 1.81419 | -2.90299 | -1.3786 | 5.00E-05 | 0.00217928 |
| ENSG00000188486.3 | H2AFX | chr11:118964563-118966177 | 5.36214 | 2.41115 | -2.95099 | -1.15309 | 0.00025 | 0.0089375 |
| ENSG00000163344.5 | PMVK | chr1:154897209-154909467 | 5.09729 | 2.08013 | -3.01716 | -1.29306 | 0.00155 | 0.0410976 |
| ENSG00000181467.2 | RAP2B | chr3:152880028-152886265 | 5.97181 | 2.87563 | -3.09618 | -1.05429 | 0.00015 | 0.00576752 |
| ENSG00000163947.7 | ARHGEF3 | chr3:56761445-57113357 | 4.39347 | 1.29695 | -3.09652 | -1.76024 | 0.0014 | 0.0380722 |
| ENSG00000169515.5 | CCDC8 | chr19:46913628-46916841 | 4.1469 | 0.746504 | -3.400396 | -2.47381 | 5.00E-05 | 0.00217928 |
| **Gene_ID** | **Gene** | **Position** | **FPKM (Control)** | **FPKM (IL1b)** | **FPKM (Absolute Change)** | **Log2**  **(fold change)** | **p-value** | **q-value** |
| ENSG00000010295.15 | IFFO1 | chr12:6647540-6665239 | 5.83165 | 2.42319 | -3.40846 | -1.267 | 5.00E-05 | 0.00217928 |
| ENSG00000147145.8 | LPAR4 | chrX:78003205-78012591 | 4.68853 | 1.16989 | -3.51864 | -2.00277 | 0.0008 | 0.024127 |
| ENSG00000134222.12 | PSRC1 | chr1:109822177-109825808 | 5.06046 | 1.51148 | -3.54898 | -1.74331 | 5.00E-05 | 0.00217928 |
| ENSG00000132436.7 | FIGNL1 | chr7:50511830-50518088 | 7.02224 | 3.16504 | -3.8572 | -1.14971 | 0.0003 | 0.0104605 |
| ENSG00000140368.8 | PSTPIP1 | chr15:77285699-77329673 | 6.07969 | 2.21198 | -3.86771 | -1.45866 | 0.0008 | 0.024127 |
| ENSG00000100625.8 | SIX4 | chr14:61176245-61191066 | 5.91776 | 1.95979 | -3.95797 | -1.59435 | 5.00E-05 | 0.00217928 |
| ENSG00000107816.13 | LZTS2 | chr10:102756374-102790890 | 5.63914 | 1.65505 | -3.98409 | -1.7686 | 5.00E-05 | 0.00217928 |
| ENSG00000100100.8 | PIK3IP1 | chr22:31677578-31688520 | 8.43524 | 4.13584 | -4.2994 | -1.02825 | 0.00105 | 0.03003 |
| ENSG00000183578.5 | TNFAIP8L3 | chr15:51348794-51397473 | 6.06992 | 1.73232 | -4.3376 | -1.80897 | 5.00E-05 | 0.00217928 |
| ENSG00000198728.6 | LDB1 | chr10:103867316-103880210 | 7.87601 | 3.5349 | -4.34111 | -1.15579 | 0.0003 | 0.0104605 |
| ENSG00000258947.2 | TUBB3 | chr16:89978526-90005169 | 7.6059 | 3.13898 | -4.46692 | -1.27682 | 0.0001 | 0.00409228 |
| ENSG00000189184.7 | PCDH18 | chr4:138440071-138453648 | 8.52658 | 3.97751 | -4.54907 | -1.1001 | 5.00E-05 | 0.00217928 |
| ENSG00000165507.8 | C10orf10 | chr10:45454854-45491339 | 5.47173 | 0.694515 | -4.777215 | -2.97792 | 5.00E-05 | 0.00217928 |
| ENSG00000112559.9 | MDFI | chr6:41604619-41621984 | 6.71822 | 1.83818 | -4.88004 | -1.8698 | 0.00195 | 0.0497348 |
| **Gene_ID** | **Gene** | **Position** | **FPKM (Control)** | **FPKM (IL1b)** | **FPKM (Absolute Change)** | **Log2**  **(fold change)** | **p-value** | **q-value** |
| ENSG00000174282.7 | ZBTB4 | chr17:7362684-7387582 | 11.6065 | 6.5104 | -5.0961 | -0.834115 | 0.00095 | 0.0276674 |
| ENSG00000108819.9 | PPP1R9B | chr17:48211103-48227991 | 9.20912 | 3.59824 | -5.61088 | -1.35577 | 0.0016 | 0.0420727 |
| ENSG00000189060.4 | H1F0 | chr22:38201113-38203442 | 13.2008 | 7.38594 | -5.81486 | -0.837769 | 0.0016 | 0.0420727 |
| ENSG00000100439.6 | ABHD4 | chr14:23067145-23081265 | 12.254 | 6.17004 | -6.08396 | -0.989901 | 0.0007 | 0.0215191 |
| ENSG00000107819.9 | SFXN3 | chr10:102790990-102800998 | 13.6119 | 7.15642 | -6.45548 | -0.927555 | 0.00155 | 0.0410976 |
| ENSG00000123485.7 | HJURP | chr2:234684369-234763212 | 12.315 | 5.7629 | -6.5521 | -1.09554 | 0.0013 | 0.0359676 |
| ENSG00000141232.4 | TOB1 | chr17:48939583-48945339 | 12.1427 | 5.568 | -6.5747 | -1.12486 | 0.00035 | 0.0119103 |
| ENSG00000128567.12 | PODXL | chr7:131185020-131242976 | 11.8008 | 5.03737 | -6.76343 | -1.22814 | 0.00065 | 0.020177 |
| ENSG00000166292.7 | TMEM100 | chr17:53796987-53809482 | 9.01707 | 2.17319 | -6.84388 | -2.05285 | 5.00E-05 | 0.00217928 |
| ENSG00000168916.11 | ZNF608 | chr5:123972607-124084500 | 9.60289 | 2.50833 | -7.09456 | -1.93674 | 0.00065 | 0.020177 |
| ENSG00000138172.6 | CALHM2 | chr10:105206542-105212660 | 11.7407 | 4.52654 | -7.21416 | -1.37504 | 5.00E-05 | 0.00217928 |
| ENSG00000197837.3 | HIST4H4 | chr12:14920932-14924065 | 11.9422 | 4.65026 | -7.29194 | -1.36069 | 0.0011 | 0.0311104 |
| ENSG00000180447.5 | GAS1 | chr9:89559278-89562104 | 14.4323 | 6.57587 | -7.85643 | -1.13405 | 5.00E-05 | 0.00217928 |
| ENSG00000177283.4 | FZD8 | chr10:35927176-35930362 | 13.8506 | 5.86017 | -7.99043 | -1.24093 | 5.00E-05 | 0.00217928 |
| **Gene_ID** | **Gene** | **Position** | **FPKM (Control)** | **FPKM (IL1b)** | **FPKM (Absolute Change)** | **Log2**  **(fold change)** | **p-value** | **q-value** |
| ENSG00000166483.6 | WEE1 | chr11:9595227-9615004 | 13.0446 | 4.75996 | -8.28464 | -1.45443 | 0.0015 | 0.040022 |
| ENSG00000065054.9 | SLC9A3R2 | chr16:2075356-2089027 | 14.6538 | 6.2038 | -8.45 | -1.24005 | 0.0001 | 0.00409228 |
| ENSG00000173281.4 | PPP1R3B | chr8:8993764-9009084 | 10.3097 | 1.76123 | -8.54847 | -2.54934 | 5.00E-05 | 0.00217928 |
| ENSG00000184702.13 | Sep-05 | chr22:19701986-19712295 | 15.6953 | 6.66035 | -9.03495 | -1.23666 | 0.0005 | 0.0163167 |
| ENSG00000108840.11 | HDAC5 | chr17:42154113-42201070 | 14.3332 | 5.27193 | -9.06127 | -1.44296 | 0.0005 | 0.0163167 |
| ENSG00000125965.4 | GDF5 | chr20:34020826-34042568 | 15.0017 | 5.49714 | -9.50456 | -1.44837 | 5.00E-05 | 0.00217928 |
| ENSG00000124610.3 | HIST1H1A | chr6:26017259-26018040 | 20.1136 | 10.2605 | -9.8531 | -0.971064 | 0.001 | 0.0289909 |
| ENSG00000117289.7 | TXNIP | chr1:145438468-145442635 | 12.7938 | 2.35157 | -10.44223 | -2.44374 | 5.00E-05 | 0.00217928 |
| ENSG00000146411.5 | SLC2A12 | chr6:134273307-134373774 | 21.841 | 10.931 | -10.91 | -0.998615 | 0.00165 | 0.0430319 |
| ENSG00000164442.8 | CITED2 | chr6:139693392-139695757 | 26.2005 | 13.3883 | -12.8122 | -0.968622 | 0.00025 | 0.0089375 |
| ENSG00000157514.12 | TSC22D3 | chrX:106956450-107020572 | 21.3287 | 8.14901 | -13.17969 | -1.3881 | 0.00015 | 0.00576752 |
| ENSG00000004799.7 | PDK4 | chr7:95212810-95225803 | 18.4864 | 4.8543 | -13.6321 | -1.92913 | 5.00E-05 | 0.00217928 |
| ENSG00000162614.14 | NEXN | chr1:78354197-78409580 | 30.5769 | 14.1462 | -16.4307 | -1.11202 | 0.00135 | 0.037109 |
| ENSG00000139263.7 | LRIG3 | chr12:59265930-59314303 | 23.9509 | 7.4206 | -16.5303 | -1.69047 | 0.0009 | 0.0265761 |
| **Gene_ID** | **Gene** | **Position** | **FPKM (Control)** | **FPKM (IL1b)** | **FPKM (Absolute Change)** | **Log2**  **(fold change)** | **p-value** | **q-value** |
| ENSG00000140465.9 | CYP1A1 | chr15:75011882-75017951 | 32.816 | 15.9567 | -16.8593 | -1.04024 | 0.00075 | 0.0228903 |
| ENSG00000173848.14 | NET1 | chr10:5454513-5500426 | 23.4937 | 6.26921 | -17.22449 | -1.90592 | 5.00E-05 | 0.00217928 |
| ENSG00000112759.12 | SLC29A1 | chr6:44187241-44201888 | 29.6498 | 12.1359 | -17.5139 | -1.28874 | 5.00E-05 | 0.00217928 |
| ENSG00000198374.3 | HIST1H2AL | chr6:27833033-27833606 | 38.1513 | 19.4317 | -18.7196 | -0.973321 | 0.0015 | 0.040022 |
| ENSG00000138449.6 | SLC40A1 | chr2:190425304-190448484 | 37.6905 | 17.2319 | -20.4586 | -1.12912 | 5.00E-05 | 0.00217928 |
| ENSG00000106211.8 | HSPB1 | chr7:75931860-75933612 | 35.9792 | 14.6563 | -21.3229 | -1.29565 | 5.00E-05 | 0.00217928 |
| ENSG00000126778.7 | SIX1 | chr14:61110132-61124977 | 32.5929 | 8.31476 | -24.27814 | -1.97081 | 5.00E-05 | 0.00217928 |
| ENSG00000101335.5 | MYL9 | chr20:35169886-35178228 | 49.3388 | 22.0707 | -27.2681 | -1.16059 | 0.0001 | 0.00409228 |
| ENSG00000197061.3 | HIST1H4C | chr6:26104103-26104518 | 60.3797 | 30.4211 | -29.9586 | -0.988991 | 0.0014 | 0.0380722 |
| ENSG00000196532.4 | HIST1H3C | chr6:26045638-26046097 | 69.4929 | 36.4939 | -32.999 | -0.929209 | 0.0014 | 0.0380722 |
| ENSG00000115641.14 | FHL2 | chr2:105974168-106054970 | 79.0866 | 39.3374 | -39.7492 | -1.00753 | 0.0008 | 0.024127 |
| ENSG00000112773.11 | FAM46A | chr6:82201155-82462491 | 80.2087 | 39.0179 | -41.1908 | -1.03962 | 0.0001 | 0.00409228 |
| ENSG00000184258.5 | CDR1 | chrX:139865424-139866723 | 94.4043 | 48.645 | -45.7593 | -0.956561 | 0.00015 | 0.00576752 |
| ENSG00000176046.7 | NUPR1 | chr16:28548605-28550495 | 113.463 | 64.5758 | -48.8872 | -0.813158 | 0.00105 | 0.03003 |
| **Gene_ID** | **Gene** | **Position** | **FPKM (Control)** | **FPKM (IL1b)** | **FPKM (Absolute Change)** | **Log2**  **(fold change)** | **p-value** | **q-value** |
| ENSG00000179010.10 | MRFAP1 | chr4:6641817-6644472 | 99.6499 | 43.6431 | -56.0068 | -1.19111 | 0.00035 | 0.0119103 |
| ENSG00000142871.11 | CYR61 | chr1:86046443-86049645 | 68.8936 | 12.1456 | -56.748 | -2.50394 | 5.00E-05 | 0.00217928 |
| ENSG00000198373.8 | WWP2 | chr16:69796208-69975644 | 71.923 | 14.7769 | -57.1461 | -2.2831 | 5.00E-05 | 0.00217928 |
| ENSG00000119938.8 | PPP1R3C | chr10:93388198-93392811 | 116.433 | 59.2704 | -57.1626 | -0.974117 | 0.0001 | 0.00409228 |
| ENSG00000144136.6 | SLC20A1 | chr2:113403433-113421404 | 104.233 | 44.5146 | -59.7184 | -1.22746 | 0.00105 | 0.03003 |
| ENSG00000184840.7 | TMED9 | chr5:177019158-177023125 | 146.011 | 75.4864 | -70.5246 | -0.951784 | 0.00015 | 0.00576752 |
| ENSG00000134294.9 | SLC38A2 | chr12:46751971-46766650 | 192.347 | 53.3827 | -138.9643 | -1.84926 | 5.00E-05 | 0.00217928 |
| ENSG00000100292.12 | HMOX1 | chr22:35776353-35790207 | 326.248 | 154.124 | -172.124 | -1.08188 | 0.00025 | 0.0089375 |
| ENSG00000198695.2 | MT-ND6 | chrM:14148-14673 | 370.863 | 189.678 | -181.185 | -0.967337 | 0.0003 | 0.0104605 |
