# Supplementary material for: Long Intergenic Noncoding RNAs Mediate the Human Chondrocyte Inflammatory Response and Are Differentially Expressed in Osteoarthritis Cartilage
Source: Arthritis Rheumatol. 2016 Mar 28;68(4):845–56. doi: 10.1002/art.39520 (PMC4950001; doi:10.1002/art.39520)
Supplement: Supplementary file 6 — Supplementary Table 3 [file ART-68-845-s006.docx]

Supplementary Table 3

| **LncRNA number** | **Position** | **Strand** | **Control (FPKM)** | **IL1b (FPKM)** | **LncRNA Class** | **GenCode Number** | **Gencode Name** | **Human Body Map Number** | **Human Body Map Name** | **FANTOM eRNA** |
| --- | --- | --- | --- | --- | --- | --- | --- | --- | --- | --- |
| XLOC_000003 | chr1:778587-793065 | + | 2.33774 | 2.36005 | LincRNA | ENSG00000228794.4 | RP11-206L10.11 | . | . | . |
| XLOC_000013 | chr1:998476-1001791 | + | 0.382595 | 1.88678 | Pseudogene | ENSG00000217801.5 | RP11-465B22.3 | . | . | . |
| XLOC_000101 | chr1:9233846-9252222 | + | 2.88599 | 1.49779 | LincRNA | ENSG00000234546.2 | RP3-510D11.2 | . | . | . |
| XLOC_000289 | chr1:22351819-22358062 | + | 1.89569 | 1.16701 | LincRNA | ENSG00000218510.3 | LINC00339 | XLOC_000732 | linc-HSPG2 | . |
| XLOC_000593 | chr1:39325641-39342454 | + | 2.74969 | 1.65458 | Antisense | ENSG00000228436.2 | RP5-864K19.4 | . | . | . |
| XLOC_000615 | chr1:39507977-39513981 | + | 1.65335 | 0.944742 | LincRNA | . | . | XLOC_000152 | linc-MACF1-2 | . |
| XLOC_000695 | chr1:41898869-41930479 | + | 1.00471 | 0.540618 | LincRNA | . | . | XLOC_000160 | linc-GUCA2B | . |
| XLOC_000722 | chr1:43390943-43439830 | + | 0.425435 | 1.19544 | LincRNA | ENSG00000227533.1 | SLC2A1-AS1 | . | . | . |
| XLOC_000753 | chr1:44513009-44542306 | + | 5.26866 | 4.78485 | LincRNA | ENSG00000230615.2 | RP5-1198O20.4 | . | . | . |
| XLOC_000831 | chr1:46908508-46948163 | + | 1.46941 | 0.776173 | LincRNA | ENSG00000225667.1 | LINC00505 | XLOC_000170 | linc-DMBX1 | . |
| XLOC_000852 | chr1:51443540-51477175 | + | 2.93753 | 1.81668 | LincRNA | . | . | XLOC_000833 | linc-FAF1-1 | . |
| XLOC_000992 | chr1:59180564-59181401 | + | 0.332379 | 1.90473 | LincRNA | . | . | XLOC_000218 | linc-FGGY-4 | . |
| XLOC_001015 | chr1:59486083-59508449 | + | 1.78934 | 1.09247 | LincRNA | ENSG00000230812.1 | RP4-794H19.4 | . | . | . |
| XLOC_001518 | chr1:89873116-89891927 | + | 0.58582 | 5.96536 | Pseudogene | ENSG00000225492.2 | GBP1P1 | . | . | . |
| XLOC_001588 | chr1:94792769-94800291 | + | 1.90713 | 4.57882 | LincRNA | ENSG00000226835.1 | RP11-148B18.3 | XLOC_000301 | linc-ABCD3-1 | . |
| XLOC_001595 | chr1:95392966-95429184 | + | 2.55538 | 2.12831 | LincRNA | ENSG00000235501.1 | RP4-639F20.1 | XLOC_000304 | linc-TMEM56-3 | . |
| XLOC_001610 | chr1:96208581-96227515 | + | 1.2109 | 2.87229 | LincRNA | ENSG00000271252.1 | RP11-286B14.2 | . | . | . |
| XLOC_001719 | chr1:101544309-101552822 | + | 1.59551 | 2.1641 | LincRNA | ENSG00000233184.2 | RP11-421L21.3 | . | . | . |
| XLOC_001882 | chr1:113498995-113511291 | + | 1.48856 | 1.4722 | LincRNA | ENSG00000226419.2 | RP11-31F15.1 | . | . | . |
| XLOC_001957 | chr1:116082085-116088417 | + | 0.551295 | 2.05392 | LincRNA | . | . | . | . | . |
| XLOC_002027 | chr1:119573396-119726625 | + | 4.18836 | 2.06279 | Antisense | ENSG00000231365.1 | RP11-418J17.1 | . | . | . |
| XLOC_002085 | chr1:120904664-120910305 | + | 1.07655 | 1.06632 | Pseudogene | ENSG00000213244.3 | HIST2H3DP1 | . | . | . |
| XLOC_002093 | chr1:121091626-121136919 | + | 2.60625 | 2.41491 | LincRNA | ENSG00000227082.1 | AL592494.5 | . | . | . |
| XLOC_002166 | chr1:143913830-144048991 | + | 5.71203 | 3.87511 | Pseudogene | ENSG00000196369.6 | SRGAP2B | . | . | . |
| XLOC_002188 | chr1:144598516-144609914 | + | 2.4876 | 2.3641 | Pseudogene | ENSG00000225241.3 | RP11-640M9.2 | . | . | . |
| XLOC_002232 | chr1:145096307-145119491 | + | 47.9428 | 55.5743 | LincRNA | . | . | . | . | . |
| XLOC_002240 | chr1:145382754-145383571 | + | 1.11593 | 3.29549 | LincRNA | . | . | . | . | . |
| XLOC_002261 | chr1:146626687-146651766 | + | 90.5861 | 80.3722 | Antisense | ENSG00000237188.3 | RP11-337C18.8 | XLOC_000385 | linc-CHD1L-1 | . |
| XLOC_002281 | chr1:147751388-147760095 | + | 2.15642 | 1.38053 | LincRNA | ENSG00000234283.1 | RP11-495P10.6 | . | . | . |
| XLOC_002293 | chr1:148928251-148954459 | + | 1.82033 | 0.764077 | LincRNA | ENSG00000232527.3 | RP11-14N7.2 | XLOC_001009 | linc-PPIAL4F-1 | . |
| XLOC_002413 | chr1:151810686-151811665 | + | 1.01667 | 0.546612 | Antisense | . | . | . | . | . |
| XLOC_002414 | chr1:151811839-151824315 | + | 4.13449 | 3.66661 | Antisense | ENSG00000234614.1 | AL450992.2 | . | . | . |
| XLOC_002443 | chr1:152221970-152223217 | + | 1.77033 | 1.56929 | LincRNA | ENSG00000237975.2 | FLG-AS1 | . | . | . |
| XLOC_002480 | chr1:152627932-152628889 | + | 0.421291 | 14.6233 | LincRNA | ENSG00000176075.6 | LINC00302 | XLOC_000411 | linc-LCE2D | . |
| XLOC_002535 | chr1:155533056-155721690 | + | 37.5031 | 48.4699 | Antisense | ENSG00000227673.1 | RP11-243J18.2 | . | . | . |
| XLOC_002602 | chr1:159930940-159948646 | + | 5.71044 | 4.29194 | LincRNA | ENSG00000224259.1 | RP11-48O20.4 | . | . | . |
| XLOC_002882 | chr1:176187675-176317855 | + | 0.606 | 1.19258 | Pseudogene | ENSG00000232463.1 | RP11-195C7.2 | XLOC_000471 | linc-PAPPA2-1 | . |
| XLOC_003055 | chr1:186393736-186404456 | + | 1.20559 | 1.24092 | LincRNA | . | . | . | . | . |
| XLOC_003097 | chr1:186649747-186654651 | + | 0.743088 | 4.33362 | LincRNA | ENSG00000273129.1 | RP5-973M2.2 | . | . | . |
| XLOC_003111 | chr1:192484855-192533053 | + | 0.257149 | 1.2721 | LincRNA | ENSG00000236069.1 | RP5-1011O1.3 | XLOC_001133 | linc-FAM5C-2 | . |
| XLOC_003348 | chr1:207540496-207549020 | + | 2.0128 | 5.19924 | LincRNA | . | . | . | . | . |
| XLOC_003393 | chr1:211564862-211605865 | + | 11.9605 | 11.0784 | Pseudogene | ENSG00000236809.2 | SNX25P1 | . | . | . |
| XLOC_003450 | chr1:219347189-219501046 | + | 207.86 | 63.6557 | LincRNA | ENSG00000223842.1 | RP11-135J2.3 | XLOC_001207 | linc-GPATCH2-1 | . |
| XLOC_003605 | chr1:222763330-222766883 | + | 1.24752 | 0.839124 | LincRNA | ENSG00000225265.1 | RP11-378J18.3 | XLOC_000576 | linc-MIA3-1 | . |
| XLOC_003618 | chr1:224180499-224208266 | + | 1.48301 | 1.3736 | LincRNA | ENSG00000185495.6 | RP11-504P24.4 | . | . | . |
| XLOC_003636 | chr1:225888343-225925896 | + | 2.57069 | 2.08314 | LincRNA | ENSG00000227496.1 | RP11-145A3.1 | XLOC_001231 | linc-ENAH | . |
| XLOC_003914 | chr1:247517260-247521512 | + | 0.709165 | 1.4553 | LincRNA | . | . | XLOC_000648 | linc-NLRP3-3 | . |
| XLOC_003940 | chr1:703947-762575 | - | 2.45451 | 2.24795 | LincRNA | ENSG00000237491.4 | RP11-206L10.9 | XLOC_000008 | linc-SAMD11-4 | . |
| XLOC_004008 | chr1:3653573-3663923 | - | 2.40693 | 1.5964 | LincRNA | ENSG00000227372.6 | TP73-AS1 | . | . | . |
| XLOC_004137 | chr1:16793578-16805433 | - | 1.97739 | 0.713471 | Pseudogene | ENSG00000080947.10 | CROCCP3 | . | . | . |
| XLOC_004149 | chr1:16943388-16971194 | - | 5.76153 | 4.83419 | LincRNA | ENSG00000215908.5 | CROCCP2 | . | . | . |
| XLOC_004158 | chr1:17011093-17013964 | - | 21.9692 | 20.5595 | Pseudogene | ENSG00000268869.1 | ESPNP | . | . | . |
| XLOC_004221 | chr1:23604933-23620014 | - | 1.43512 | 1.34875 | LincRNA | ENSG00000261326.2 | RP5-1057J7.6 | XLOC_000734 | linc-HTR1D | . |
| XLOC_004322 | chr1:28905072-28908382 | - | 15.29 | 15.2207 | LincRNA | ENSG00000197989.9 | SNHG12 | . | . | . |
| XLOC_004383 | chr1:32704674-32706111 | - | 1.002 | 0.412666 | Pseudogene | ENSG00000220785.3 | MTMR9LP | . | . | . |
| XLOC_004480 | chr1:37920479-37948615 | - | 1.10117 | 3.91064 | LincRNA | ENSG00000233621.1 | RP11-422J8.1 | XLOC_000787 | linc-GRIK3-2 | eRNA |
| XLOC_004507 | chr1:39987857-40008313 | - | 1.4294 | 2.46082 | LincRNA | ENSG00000182109.3 | RP11-69E11.4 | . | . | . |
| XLOC_004518 | chr1:40012505-40025342 | - | 1.34277 | 1.71574 | LincRNA | ENSG00000182109.3 | RP11-69E11.4 | . | . | . |
| XLOC_004669 | chr1:47846304-47874016 | - | 0.710761 | 1.10438 | LincRNA | ENSG00000225762.1 | RP11-511I2.2 | XLOC_000822 | linc-STIL-2 | . |
| XLOC_004670 | chr1:47874121-47903334 | - | 1.11553 | 0.432375 | Antisense | ENSG00000225762.1 | RP11-511I2.2 | XLOC_000822 | linc-STIL-2 | . |
| XLOC_004949 | chr1:56880628-56925281 | - | 0.416454 | 2.46429 | LincRNA | ENSG00000223956.1 | RP4-710M16.2 | XLOC_000214 | linc-PRKAA2-1 | . |
| XLOC_004994 | chr1:59349249-59377695 | - | 1.07509 | 0.324291 | LincRNA | ENSG00000272226.1 | RP11-63G10.3 | . | . | eRNA |
| XLOC_005041 | chr1:65437492-65468317 | - | 1.2302 | 4.67309 | Pseudogene | ENSG00000185031.6 | SLC2A3P2 | . | . | . |
| XLOC_005304 | chr1:89006045-89273429 | - | 0.413348 | 1.72515 | Antisense | ENSG00000237505.2 | RP11-76N22.2 | . | . | . |
| XLOC_005391 | chr1:91248564-91254989 | - | 1.16323 | 1.67762 | LincRNA | ENSG00000233593.2 | RP4-665J23.1 | XLOC_000293 | linc-CDC7 | . |
| XLOC_005395 | chr1:91297301-91317175 | - | 1.35975 | 1.09066 | LincRNA | ENSG00000233593.2 | RP4-665J23.1 | XLOC_000293 | linc-CDC7 | . |
| XLOC_005453 | chr1:93691222-93739064 | - | 1.40759 | 1.71818 | Antisense | ENSG00000223745.3 | RP4-717I23.3 | . | . | . |
| XLOC_005477 | chr1:93791326-93811287 | - | 4.5695 | 4.25173 | LincRNA | ENSG00000223745.3 | RP4-717I23.3 | XLOC_000918 | linc-TMED5 | . |
| XLOC_005601 | chr1:100724075-100731683 | - | 1.80544 | 0.653495 | LincRNA | ENSG00000224616.1 | RP11-305E17.6 | XLOC_000939 | linc-DBT-3 | . |
| XLOC_005753 | chr1:111905991-111911108 | - | 0 | 2.499 | Pseudogene | ENSG00000234020.1 | CHIAP3 | . | . | . |
| XLOC_006031 | chr1:119349587-119409083 | - | 1.92565 | 9.23502 | LincRNA | . | . | . | . | . |
| XLOC_006147 | chr1:144518606-144521345 | - | 1.97971 | 1.97455 | Antisense | ENSG00000236943.2 | RP11-640M9.1 | . | . | . |
| XLOC_006191 | chr1:146494290-146495720 | - | 2.09365 | 1.92586 | Pseudogene | ENSG00000227242.2 | NBPF13P | . | . | . |
| XLOC_006193 | chr1:146527977-146542193 | - | 14.2817 | 34.1757 | Pseudogene | ENSG00000227242.2 | NBPF13P | . | . | . |
| XLOC_006385 | chr1:153761245-153762791 | - | 0.0864682 | 2.95862 | LincRNA | . | . | . | . | eRNA |
| XLOC_006759 | chr1:173408867-173431580 | - | 1.10833 | 1.34484 | LincRNA | ENSG00000203739.3 | RP11-296O14.3 | . | . | . |
| XLOC_006784 | chr1:173830721-173837141 | - | 17.5871 | 11.4845 | LincRNA | ENSG00000234741.3 | GAS5 | XLOC_000467 | linc-ZBTB37 | . |
| XLOC_006814 | chr1:175873617-175877940 | - | 0.860181 | 1.65357 | LincRNA | ENSG00000227740.1 | RP11-318C24.2 | XLOC_001099 | linc-TNR-2 | . |
| XLOC_006875 | chr1:180918817-180925073 | - | 1.12737 | 1.19915 | Antisense | ENSG00000243155.1 | RP11-46A10.5 | . | . | . |
| XLOC_007192 | chr1:201452654-201485577 | - | 0.63472 | 1.12183 | LincRNA | ENSG00000224536.1 | RP11-134G8.7 | . | . | eRNA |
| XLOC_007267 | chr1:205658191-205681581 | - | 2.09234 | 1.22425 | LincRNA | . | . | . | . | . |
| XLOC_007344 | chr1:207970992-207999455 | - | 3.99971 | 2.97588 | LincRNA | ENSG00000203709.5 | C1orf132 | XLOC_001181 | linc-YOD1-2 | eRNA |
| XLOC_007353 | chr1:208028011-208042402 | - | 1.17797 | 1.06073 | LincRNA | ENSG00000203709.5 | C1orf132 | . | . | . |
| XLOC_007397 | chr1:210404824-210407067 | - | 1.59228 | 2.9157 | Antisense | ENSG00000203706.4 | SERTAD4-AS1 | . | . | . |
| XLOC_007400 | chr1:210463634-210466365 | - | 0.293409 | 1.34162 | LincRNA | . | . | XLOC_001188 | linc-IRF6 | . |
| XLOC_007419 | chr1:212398629-212408088 | - | 1.7695 | 0.713506 | LincRNA | ENSG00000226251.1 | RP11-15I11.3 | XLOC_001191 | linc-INTS7 | . |
| XLOC_007431 | chr1:213009355-213031499 | - | 3.33011 | 1.27945 | LincRNA | ENSG00000198468.3 | FLVCR1-AS1 | XLOC_001197 | linc-C1orf227-1 | . |
| XLOC_007469 | chr1:219259841-219323400 | - | 1.90364 | 0.742316 | LincRNA | ENSG00000228063.1 | RP11-135J2.4 | . | . | . |
| XLOC_007478 | chr1:219589002-219589815 | - | 1.74182 | 2.2518 | LincRNA | . | . | XLOC_001209 | linc-GPATCH2-8 | . |
| XLOC_007480 | chr1:219597501-219613213 | - | 2.04872 | 6.20003 | LincRNA | ENSG00000230024.1 | RP11-95P13.1 | XLOC_000567 | linc-IARS2-2 | . |
| XLOC_007589 | chr1:222212915-222238159 | - | 1.29329 | 2.03043 | LincRNA | ENSG00000232679.1 | RP11-400N13.3 | XLOC_001215 | linc-DUSP10-2 | . |
| XLOC_007649 | chr1:224148739-224150419 | - | 1.0899 | 1.10603 | LincRNA | . | . | . | . | . |
| XLOC_007657 | chr1:224161555-224164705 | - | 0.894702 | 1.55074 | LincRNA | . | . | . | . | . |
| XLOC_007881 | chr1:235096832-235100692 | - | 0.129156 | 1.12895 | LincRNA | ENSG00000238005.2 | RP11-443B7.1 | XLOC_000627 | linc-GGPS1-1 | . |
| XLOC_007932 | chr1:238635649-238649582 | - | 3.22789 | 2.07717 | LincRNA | ENSG00000231877.1 | RP11-177F15.1 | XLOC_001272 | linc-ZP4-1 | . |
| XLOC_008032 | chr1:247350550-247374185 | - | 2.69746 | 3.91837 | LincRNA | ENSG00000259865.1 | RP11-488L18.10 | . | . | . |
| XLOC_008063 | chr10:1068687-1073896 | + | 0.998609 | 1.40944 | Antisense | ENSG00000232656.3 | IDI2-AS1 | . | . | . |
| XLOC_008242 | chr10:20105109-20648712 | + | 76.8247 | 77.3556 | Pseudogene | ENSG00000228339.1 | AMD1P1 | . | . | . |
| XLOC_008349 | chr10:27854924-27867588 | + | 0.704728 | 1.38642 | LincRNA | . | . | . | . | . |
| XLOC_008421 | chr10:30397102-30411761 | + | 1.08409 | 1.4629 | Antisense | . | . | . | . | . |
| XLOC_008452 | chr10:30485570-30495964 | + | 1.72542 | 1.65484 | LincRNA | . | . | . | . | . |
| XLOC_008471 | chr10:30974223-30978375 | + | 32.4573 | 39.5892 | Pseudogene | ENSG00000234814.3 | SVILP1 | . | . | . |
| XLOC_008562 | chr10:38658006-38669044 | + | 1.32 | 0.826135 | Pseudogene | ENSG00000099251.10 | HSD17B7P2 | . | . | . |
| XLOC_008795 | chr10:67330280-67449270 | + | 8.61988 | 4.62393 | LincRNA | ENSG00000228065.6 | RP11-222A11.1 | XLOC_008500 | linc-LRRTM3-3 | . |
| XLOC_008954 | chr10:74927928-75105290 | + | 1.35905 | 0.823181 | Antisense | ENSG00000236756.4 | DNAJC9-AS1 | . | . | . |
| XLOC_008960 | chr10:75255476-75257968 | + | 3.1781 | 2.46135 | Antisense | ENSG00000221817.5 | RP11-137L10.6 | . | . | . |
| XLOC_009009 | chr10:77055501-77121514 | + | 2.3013 | 2.53528 | Pseudogene | ENSG00000233313.2 | HMGA1P5 | . | . | . |
| XLOC_009027 | chr10:77191281-77211897 | + | 0.72112 | 1.65377 | LincRNA | ENSG00000236842.1 | RP11-399K21.10 | . | . | . |
| XLOC_009132 | chr10:81677835-81681741 | + | 1.60301 | 0.847622 | Pseudogene | ENSG00000242600.2 | MBL1P | XLOC_008531 | linc-C10orf57-2 | . |
| XLOC_009168 | chr10:88753116-88769995 | + | 1.94565 | 1.53513 | Pseudogene | ENSG00000240089.2 | BMS1P3 | . | . | . |
| XLOC_009179 | chr10:89102740-89114919 | + | 2.21763 | 1.74072 | LincRNA | ENSG00000224914.2 | LINC00863 | . | . | . |
| XLOC_009289 | chr10:93284331-93335376 | + | 1.1459 | 0.677805 | LincRNA | . | . | XLOC_008562 | linc-TNKS2-2 | . |
| XLOC_009337 | chr10:97889491-97928287 | + | 6.74107 | 5.25127 | LincRNA | . | . | . | . | . |
| XLOC_009384 | chr10:100209111-100213468 | + | 0.647623 | 1.40533 | LincRNA | . | . | . | . | . |
| XLOC_009474 | chr10:104593023-104594458 | + | 0.384285 | 2.05774 | Antisense | ENSG00000203886.4 | CYP17A1-AS1 | . | . | . |
| XLOC_009674 | chr10:119806495-119859452 | + | 1.19567 | 0.526931 | LincRNA | ENSG00000177640.11 | CASC2 | . | . | . |
| XLOC_009752 | chr10:123499939-123711472 | + | 1.2455 | 1.06278 | LincRNA | ENSG00000226864.1 | RP11-500G22.2 | . | . | . |
| XLOC_010005 | chr10:1095490-1102645 | - | 1.65434 | 1.33968 | Antisense | . | . | . | . | . |
| XLOC_010019 | chr10:4248108-4271817 | - | 2.20624 | 1.06196 | LincRNA | ENSG00000233117.2 | LINC00702 | XLOC_008700 | linc-KLF6-2 | . |
| XLOC_010032 | chr10:4276289-4286146 | - | 3.07599 | 2.39321 | LincRNA | ENSG00000233117.2 | LINC00702 | XLOC_008701 | linc-KLF6-1 | . |
| XLOC_010053 | chr10:4693099-4700247 | - | 0.881569 | 1.3004 | LincRNA | ENSG00000231298.2 | LINC00704 | XLOC_008362 | linc-AKR1E2-1 | . |
| XLOC_010108 | chr10:5317547-5328142 | - | 0.847387 | 1.9453 | Pseudogene | ENSG00000215267.4 | AKR1C7P | . | . | . |
| XLOC_010345 | chr10:18802237-18830740 | - | 1.4578 | 1.0761 | Antisense | ENSG00000225527.1 | RP11-383B4.4 | . | . | . |
| XLOC_010441 | chr10:25279059-25351246 | - | 0 | 1.03969 | LincRNA | . | . | . | . | . |
| XLOC_010506 | chr10:28808737-28820772 | - | 8.49569 | 6.49201 | LincRNA | ENSG00000254635.1 | WAC-AS1 | . | . | . |
| XLOC_010579 | chr10:31605335-31608060 | - | 1.36223 | 1.2788 | Antisense | ENSG00000237036.4 | ZEB1-AS1 | . | . | . |
| XLOC_010614 | chr10:33178524-33187638 | - | 2.0147 | 2.23861 | LincRNA | . | . | . | . | . |
| XLOC_010712 | chr10:43007054-43046615 | - | 3.03774 | 1.87775 | Pseudogene | ENSG00000234420.3 | ZNF37BP | . | . | . |
| XLOC_011112 | chr10:75459480-75478135 | - | 8.59944 | 6.16859 | Pseudogene | ENSG00000242338.2 | BMS1P4 | . | . | . |
| XLOC_011114 | chr10:75480294-75487244 | - | 3.65346 | 4.63933 | Pseudogene | ENSG00000242338.2 | BMS1P4 | . | . | . |
| XLOC_011116 | chr10:75493017-75536024 | - | 4.95676 | 7.59095 | LincRNA | . | . | . | . | . |
| XLOC_011186 | chr10:80703082-80722609 | - | 6.37564 | 3.31202 | LincRNA | ENSG00000224596.3 | ZMIZ1-AS1 | XLOC_008863 | linc-POLR3A-1 | . |
| XLOC_011255 | chr10:81477107-81569392 | - | 4.90876 | 4.96663 | LincRNA | ENSG00000226381.3 | RP11-119F19.2 | . | . | . |
| XLOC_011334 | chr10:89076441-89102298 | - | 6.26824 | 7.41849 | LincRNA | ENSG00000223482.3 | NUTM2A-AS1 | . | . | . |
| XLOC_011563 | chr10:101405432-101419029 | - | 0.720763 | 1.878 | LincRNA | ENSG00000229278.1 | RP11-483F11.7 | . | . | . |
| XLOC_011636 | chr10:106061587-106065086 | - | 0.197672 | 2.30482 | Antisense | . | . | . | . | . |
| XLOC_011641 | chr10:106110780-106113335 | - | 0.347492 | 2.32445 | LincRNA | ENSG00000231233.1 | CCDC147-AS1 | XLOC_008937 | linc-ITPRIP-1 | . |
| XLOC_011671 | chr10:110930813-110964109 | - | 0.170375 | 1.07001 | LincRNA | . | . | . | . | . |
| XLOC_011761 | chr10:116552178-116621238 | - | 2.67508 | 3.22935 | Pseudogene | ENSG00000215760.2 | TAF9BP2 | XLOC_008957 | linc-ABLIM1-1 | eRNA |
| XLOC_011786 | chr10:118590561-118609810 | - | 1.49315 | 0.756474 | LincRNA | ENSG00000225302.1 | RP11-539I5.1 | . | . | . |
| XLOC_011947 | chr10:128101773-128148058 | - | 3.41687 | 2.32781 | LincRNA | ENSG00000235180.1 | LINC00601 | XLOC_008985 | linc-ADAM12 | . |
| XLOC_012100 | chr11:8987385-8989682 | + | 1.17951 | 1.16677 | LincRNA | ENSG00000254860.1 | TMEM9B-AS1 | . | . | . |
| XLOC_012132 | chr11:10879770-10900813 | + | 4.462 | 4.8172 | Antisense | ENSG00000250041.2 | CTD-2003C8.2 | XLOC_009065 | linc-USP47-3 | . |
| XLOC_012170 | chr11:13984432-14290449 | + | 1.23411 | 1.10727 | LincRNA | ENSG00000254418.1 | RP11-21L19.1 | . | . | . |
| XLOC_012230 | chr11:18610338-18625523 | + | 4.01446 | 4.47658 | Pseudogene | ENSG00000256282.1 | RP11-504G3.4 | XLOC_009389 | linc-UEVLD | . |
| XLOC_012307 | chr11:26849887-26876549 | + | 0.634679 | 1.85909 | LincRNA | . | . | . | . | . |
| XLOC_012325 | chr11:27528390-27535595 | + | 1.69749 | 1.37063 | LincRNA | ENSG00000245573.3 | BDNF-AS | XLOC_009092 | linc-METT5D1 | . |
| XLOC_012496 | chr11:35038376-35054428 | + | 0.0519299 | 1.04754 | LincRNA | . | . | . | . | eRNA |
| XLOC_012569 | chr11:45377019-45378356 | + | 1.39379 | 0.418016 | LincRNA | ENSG00000254427.1 | RP11-430H10.1 | XLOC_009120 | linc-SLC35C1-5 | . |
| XLOC_012660 | chr11:59383622-59387921 | + | 0.321051 | 1.0675 | LincRNA | ENSG00000255139.1 | AP000442.1 | . | . | . |
| XLOC_012771 | chr11:64013307-64015669 | + | 10.0941 | 14.7543 | Antisense | ENSG00000256940.1 | RP11-783K16.5 | . | . | . |
| XLOC_012805 | chr11:65190220-65213012 | + | 50.7982 | 45.3741 | LincRNA | ENSG00000245532.4 | NEAT1 | . | . | . |
| XLOC_013080 | chr11:82783117-82799004 | + | 2.06277 | 2.13748 | LincRNA | ENSG00000246067.3 | RAB30-AS1 | XLOC_009233 | linc-PCF11-2 | . |
| XLOC_013218 | chr11:87078182-87083856 | + | 1.00693 | 0 | LincRNA | . | . | . | . | . |
| XLOC_013397 | chr11:94800003-94804314 | + | 6.52769 | 4.40991 | Pseudogene | ENSG00000180771.10 | SRSF8 | . | . | . |
| XLOC_013413 | chr11:94966057-94967229 | + | 1.28768 | 1.40934 | LincRNA | ENSG00000245552.2 | RP11-712B9.2 | . | . | . |
| XLOC_013478 | chr11:102251554-102261900 | + | 1.74929 | 6.39946 | LincRNA | . | . | . | . | . |
| XLOC_013525 | chr11:102917615-102962923 | + | 0.537629 | 4.63491 | Antisense | . | . | . | . | . |
| XLOC_013599 | chr11:112161030-112165508 | + | 2.65608 | 2.88679 | LincRNA | . | . | . | . | . |
| XLOC_013668 | chr11:114332438-114339861 | + | 2.00733 | 1.21843 | LincRNA | . | . | . | . | . |
| XLOC_013671 | chr11:114344162-114353855 | + | 1.54209 | 0.753449 | LincRNA | . | . | . | . | . |
| XLOC_013682 | chr11:114371275-114435843 | + | 2.43998 | 2.09813 | Pseudogene | ENSG00000255982.1 | NXPE2P1 | . | . | . |
| XLOC_013801 | chr11:121107905-121116704 | + | 1.66215 | 0.660911 | Pseudogene | ENSG00000254515.1 | RP11-775A1.2 | . | . | . |
| XLOC_013897 | chr11:124670576-124679313 | + | 1.27356 | 1.09802 | LincRNA | ENSG00000245498.2 | RP11-677M14.7 | . | . | . |
| XLOC_013921 | chr11:125962203-125978095 | + | 0.439307 | 23.7498 | LincRNA | ENSG00000261257.1 | RP11-673E11.2 | XLOC_009320 | linc-FAM118B | . |
| XLOC_014030 | chr11:2016399-2019050 | - | 147.449 | 109.977 | LincRNA | ENSG00000130600.11 | H19 | . | . | . |
| XLOC_014099 | chr11:5680484-5683866 | - | 1.32671 | 1.33242 | LincRNA | . | . | . | . | . |
| XLOC_014285 | chr11:9777555-9779383 | - | 2.05333 | 1.22533 | LincRNA | ENSG00000246273.2 | SBF2-AS1 | XLOC_009371 | linc-TMEM41B | . |
| XLOC_014608 | chr11:35038376-35054428 | - | 0.0347015 | 1.47552 | Antisense | . | . | . | . | eRNA |
| XLOC_014755 | chr11:49455144-49456058 | - | 2.15276 | 4.0711 | Pseudogene | ENSG00000226268.2 | RP11-61N20.3 | . | . | . |
| XLOC_014801 | chr11:58901039-58904262 | - | 1.52211 | 0.89158 | Pseudogene | ENSG00000255381.1 | AP001258.5 | XLOC_009445 | linc-GLYATL2 | . |
| XLOC_014868 | chr11:62619472-62623358 | - | 10.6004 | 11.3273 | LincRNA | ENSG00000255717.2 | SNHG1 | . | . | . |
| XLOC_014878 | chr11:63448941-63531576 | - | 2.48766 | 2.14527 | Antisense | ENSG00000188070.8 | C11orf95 | . | . | . |
| XLOC_014981 | chr11:66238709-66247704 | - | 0.936322 | 1.09239 | Antisense | ENSG00000255517.2 | CTD-3074O7.5 | . | . | . |
| XLOC_015020 | chr11:67481385-67771589 | - | 1.02981 | 0.516026 | Pseudogene | ENSG00000160172.6 | FAM86C2P | XLOC_009181 | linc-ALDH3B1-1 | . |
| XLOC_015079 | chr11:72522741-72525354 | - | 1.05821 | 1.14931 | Antisense | . | . | XLOC_009498 | linc-STARD10 | . |
| XLOC_015178 | chr11:82868148-82904678 | - | 1.60376 | 1.86784 | Antisense | ENSG00000247137.4 | RP11-727A23.5 | . | . | . |
| XLOC_015533 | chr11:107175812-107186366 | - | 1.43306 | 1.02397 | LincRNA | ENSG00000261098.1 | RP11-819C21.1 | XLOC_009544 | linc-GUCY1A2 | . |
| XLOC_015552 | chr11:110962945-110968055 | - | 6.31642 | 0.866295 | LincRNA | ENSG00000271584.1 | RP11-89C3.4 | XLOC_009549 | linc-ARHGAP20-2 | . |
| XLOC_015619 | chr11:118476724-118529056 | - | 0.446391 | 1.00961 | Antisense | . | . | . | . | eRNA |
| XLOC_015775 | chr11:122104817-122238580 | - | 2.13761 | 4.64704 | LincRNA | ENSG00000255090.1 | RP11-820L6.1 | XLOC_009307 | linc-UBASH3B-2 | . |
| XLOC_015977 | chr11:125793401-125800822 | - | 0.200828 | 1.2109 | LincRNA | ENSG00000255027.2 | RP11-680F20.9 | XLOC_009594 | linc-PUS3-2 | . |
| XLOC_016045 | chr11:133890833-133917442 | - | 0.743462 | 19.3298 | LincRNA | ENSG00000204241.3 | RP11-713P17.3 | XLOC_009341 | linc-JAM3-2 | . |
| XLOC_016068 | chr12:83767-91221 | + | 1.5257 | 1.33735 | Pseudogene | ENSG00000226210.3 | ABC7-42389800N19.1 | . | . | . |
| XLOC_016225 | chr12:7262020-7264377 | + | 3.38571 | 3.53363 | LincRNA | ENSG00000205885.3 | C1RL-AS1 | . | . | . |
| XLOC_016227 | chr12:7267515-7271591 | + | 2.55234 | 2.46841 | LincRNA | ENSG00000205885.3 | C1RL-AS1 | . | . | . |
| XLOC_016228 | chr12:7272731-7310979 | + | 6.18555 | 7.59697 | Antisense | ENSG00000205885.3 | C1RL-AS1 | XLOC_010001 | linc-RBP5-2 | . |
| XLOC_016262 | chr12:8382301-8395455 | + | 1.23285 | 0.786201 | Pseudogene | ENSG00000164845.12 | FAM86FP | . | . | . |
| XLOC_016290 | chr12:9447033-9453710 | + | 1.48785 | 1.77148 | Pseudogene | ENSG00000111788.9 | RP11-22B23.1 | . | . | . |
| XLOC_016314 | chr12:9745353-9770063 | + | 1.39586 | 1.3126 | Antisense | . | . | . | . | . |
| XLOC_016326 | chr12:9855072-9866376 | + | 0.065488 | 1.888572 | LincRNA | ENSG00000256582.1 | RP11-75L1.1 | . | . | . |
| XLOC_016482 | chr12:13076910-13153261 | + | 1.51953 | 2.96559 | Antisense | ENSG00000247498.5 | RP11-392P7.6 | . | . | eRNA |
| XLOC_016532 | chr12:14721025-14818870 | + | 2.38731 | 1.69817 | Antisense | ENSG00000256751.1 | RP11-695J4.2 | . | . | . |
| XLOC_016752 | chr12:25557338-25605277 | + | 0.312595 | 1.36201 | Pseudogene | ENSG00000255988.1 | TUBB4BP1 | . | . | . |
| XLOC_017022 | chr12:46780768-47270629 | + | 16.9423 | 12.0087 | LincRNA | ENSG00000272369.1 | RP11-446N19.1 | XLOC_009723 | linc-FAM113B-3 | . |
| XLOC_017288 | chr12:48747116-48837821 | + | 1.20029 | 1.59086 | LincRNA | ENSG00000257735.1 | RP11-370I10.6 | . | . | . |
| XLOC_017465 | chr12:54519906-54526627 | + | 3.04845 | 6.04331 | LincRNA | ENSG00000250742.1 | RP11-834C11.4 | . | . | . |
| XLOC_017486 | chr12:56122972-56125008 | + | 2.77276 | 2.0225 | Antisense | ENSG00000258056.1 | RP11-644F5.11 | . | . | . |
| XLOC_017490 | chr12:56225178-56236773 | + | 1.36773 | 1.13429 | Pseudogene | ENSG00000182796.8 | TMEM198B | . | . | . |
| XLOC_017630 | chr12:65996581-66002490 | + | 1.29002 | 1.79898 | LincRNA | ENSG00000250748.2 | RP11-230G5.2 | XLOC_009788 | linc-HMGA2 | . |
| XLOC_017660 | chr12:67716916-67743440 | + | 1.37957 | 1.79344 | LincRNA | . | . | . | . | . |
| XLOC_017736 | chr12:68322843-68415832 | + | 2.35275 | 1.65709 | LincRNA | ENSG00000255772.1 | GS1-410F4.4 | XLOC_009792 | linc-RAP1B-5 | . |
| XLOC_018006 | chr12:90354075-90465585 | + | 0.0465498 | 1.99163 | Pseudogene | ENSG00000258290.1 | RP11-654D12.1 | XLOC_009828 | linc-CLLU1-7 | . |
| XLOC_018058 | chr12:90485171-90507675 | + | 0.0346874 | 1.83942 | LincRNA | ENSG00000257194.2 | RP11-567C2.1 | XLOC_009829 | linc-CLLU1-6 | . |
| XLOC_018102 | chr12:92539860-92541264 | + | 1.25126 | 1.08222 | LincRNA | ENSG00000245904.2 | RP11-796E2.4 | . | . | . |
| XLOC_018205 | chr12:102675067-102676048 | + | 0 | 10.4673 | LincRNA | . | . | . | . | . |
| XLOC_018457 | chr12:120639112-120647677 | + | 7.8417 | 3.09507 | LincRNA | ENSG00000255857.1 | PXN-AS1 | . | . | . |
| XLOC_018564 | chr12:132907669-132908802 | + | 0.0320968 | 1.37522 | LincRNA | ENSG00000255916.1 | RP13-895J2.7 | . | . | . |
| XLOC_018661 | chr12:6547939-6553126 | - | 1.31017 | 1.14894 | LincRNA | ENSG00000215039.2 | CD27-AS1 | . | . | . |
| XLOC_018668 | chr12:6560074-6560747 | - | 4.19014 | 9.79103 | Antisense | ENSG00000215039.2 | CD27-AS1 | . | . | . |
| XLOC_018703 | chr12:8382301-8395455 | - | 1.28119 | 0.887129 | Pseudogene | ENSG00000164845.12 | FAM86FP | . | . | . |
| XLOC_018712 | chr12:9381199-9382950 | - | 0 | 3.01365 | LincRNA | ENSG00000256427.1 | RP11-118B22.4 | . | . | . |
| XLOC_018755 | chr12:10740721-10753880 | - | 1.9813 | 1.35718 | Pseudogene | ENSG00000256667.2 | KLRAP1 | XLOC_009663 | linc-PRH2-1 | . |
| XLOC_018974 | chr12:24160799-24298161 | - | 5.50561 | 4.81767 | LincRNA | . | . | . | . | . |
| XLOC_018975 | chr12:24298301-24362765 | - | 3.99282 | 3.45942 | LincRNA | . | . | . | . | . |
| XLOC_019162 | chr12:26348384-26424796 | - | 0.133753 | 4.0208 | Antisense | ENSG00000255750.1 | RP11-283G6.5 | . | . | . |
| XLOC_019309 | chr12:31267625-31270542 | - | 0 | 1.36645 | Pseudogene | ENSG00000177359.13 | RP11-551L14.1 | . | . | . |
| XLOC_019445 | chr12:47781827-47787677 | - | 0.0320559 | 1.11176 | LincRNA | . | . | . | . | . |
| XLOC_019531 | chr12:49681018-49687077 | - | 1.38919 | 0.24335 | LincRNA | ENSG00000258334.1 | RP11-161H23.9 | XLOC_010069 | linc-TUBA1A-3 | . |
| XLOC_019659 | chr12:54377711-54378821 | - | 2.119 | 1.01424 | LincRNA | ENSG00000251151.2 | HOXC-AS3 | . | . | . |
| XLOC_019748 | chr12:58325385-58329964 | - | 27.7377 | 16.4496 | LincRNA | ENSG00000257698.1 | RP11-620J15.3 | XLOC_010102 | linc-CTDSP2-1 | . |
| XLOC_019921 | chr12:67300788-67331986 | - | 1.37071 | 1.20833 | LincRNA | ENSG00000256248.1 | RP11-123O10.4 | XLOC_010109 | linc-GRIP1-1 | . |
| XLOC_020092 | chr12:72065447-72079801 | - | 4.54157 | 0 | Antisense | . | . | . | . | . |
| XLOC_020226 | chr12:89441932-89447746 | - | 0.225138 | 1.57043 | LincRNA | ENSG00000257156.1 | RP11-13A1.3 | . | . | . |
| XLOC_020319 | chr12:92652498-92682330 | - | 1.11431 | 0.928723 | LincRNA | . | . | XLOC_010151 | linc-BTG1-1 | . |
| XLOC_020327 | chr12:93583761-93585311 | - | 1.22779 | 0.917545 | LincRNA | ENSG00000257345.2 | RP11-511B23.1 | XLOC_009840 | linc-NUDT4-1 | . |
| XLOC_020345 | chr12:93936247-93971322 | - | 1.33847 | 1.72428 | Antisense | ENSG00000246985.3 | SOCS2-AS1 | . | . | . |
| XLOC_020415 | chr12:100559569-100570862 | - | 1.20421 | 0.938554 | Pseudogene | ENSG00000238105.3 | GOLGA2B | . | . | . |
| XLOC_020582 | chr12:112277135-112279783 | - | 3.67505 | 3.8756 | LincRNA | ENSG00000234608.3 | MAPKAPK5-AS1 | XLOC_010202 | linc-BRAP-2 | . |
| XLOC_020819 | chr13:21872277-21906750 | + | 1.24768 | 1.10132 | Pseudogene | ENSG00000215571.4 | GRK6P1 | . | . | . |
| XLOC_020859 | chr13:24304355-24466225 | + | 1.36499 | 0.708714 | Antisense | ENSG00000205861.7 | C1QTNF9B-AS1 | . | . | . |
| XLOC_020932 | chr13:31443220-31473057 | + | 1.92453 | 2.04135 | LincRNA | ENSG00000224743.2 | TEX26-AS1 | XLOC_010338 | linc-C13orf33-1 | . |
| XLOC_020940 | chr13:32420853-32535721 | + | 1.36606 | 2.17077 | Pseudogene | ENSG00000229715.4 | EEF1DP3 | . | . | . |
| XLOC_021107 | chr13:42916696-42958604 | + | 3.30001 | 0 | Pseudogene | ENSG00000233259.3 | FABP3P2 | . | . | . |
| XLOC_021135 | chr13:44716445-44722156 | + | 1.21958 | 1.44463 | Antisense | ENSG00000227258.1 | SMIM2-AS1 | . | . | . |
| XLOC_021268 | chr13:52027453-52037077 | + | 2.26735 | 1.34108 | Antisense | ENSG00000236778.3 | INTS6-AS1 | . | . | . |
| XLOC_021529 | chr13:92001273-92021299 | + | 0.696672 | 1.38164 | LincRNA | ENSG00000215417.6 | MIR17HG | . | . | . |
| XLOC_021949 | chr13:38626832-38633852 | - | 1.62235 | 1.98106 | LincRNA | ENSG00000223685.1 | LINC00571 | . | . | . |
| XLOC_021952 | chr13:38635521-38802218 | - | 6.49247 | 3.9889 | LincRNA | ENSG00000223685.1 | LINC00571 | . | . | . |
| XLOC_022109 | chr13:41031620-41097506 | - | 0.961792 | 1.67638 | LincRNA | ENSG00000215483.4 | LINC00598 | . | . | . |
| XLOC_022164 | chr13:41400661-41447905 | - | 1.22996 | 0.80678 | Pseudogene | ENSG00000239827.4 | SUGT1P3 | . | . | . |
| XLOC_022182 | chr13:41448019-41461964 | - | 2.30607 | 1.41259 | Pseudogene | ENSG00000239827.4 | SUGT1P3 | XLOC_010579 | linc-MRPS31 | . |
| XLOC_022197 | chr13:41486155-41496146 | - | 2.02604 | 1.51069 | Pseudogene | ENSG00000239827.4 | SUGT1P3 | . | . | . |
| XLOC_022261 | chr13:45258902-45290099 | - | 0.379947 | 1.3517 | LincRNA | ENSG00000237585.1 | LINC00407 | XLOC_010372 | linc-KIAA1704 | eRNA |
| XLOC_022366 | chr13:50548921-50557525 | - | 1.30562 | 1.21716 | LincRNA | . | . | . | . | . |
| XLOC_022372 | chr13:50571130-50699556 | - | 11.2201 | 7.33117 | LincRNA | . | . | XLOC_010389 | linc-RNASEH2B-2 | . |
| XLOC_022458 | chr13:52741183-52768573 | - | 3.24816 | 2.20318 | Pseudogene | ENSG00000243406.2 | MRPS31P5 | . | . | . |
| XLOC_022937 | chr14:22788454-22789283 | + | 1.09603 | 0.441127 | LincRNA | . | . | . | . | . |
| XLOC_022940 | chr14:22933246-22934850 | + | 1.10265 | 0.997883 | LincRNA | ENSG00000249446.2 | TRAJ60 | . | . | . |
| XLOC_023430 | chr14:53622876-53635089 | + | 0.957329 | 1.27647 | LincRNA | ENSG00000258731.1 | RP11-547D23.1 | . | . | . |
| XLOC_023700 | chr14:64108790-64118204 | + | 3.63351 | 0.940421 | LincRNA | . | . | . | . | . |
| XLOC_023743 | chr14:69328336-69329195 | + | 2.07072 | 0.375775 | Pseudogene | ENSG00000270975.1 | RP11-723P16.3 | . | . | . |
| XLOC_023790 | chr14:71164588-71179825 | + | 1.4539 | 0.699799 | LincRNA | ENSG00000258689.1 | RP6-65G23.1 | XLOC_010866 | linc-PCNX | eRNA |
| XLOC_023820 | chr14:73929077-73933619 | + | 0.284817 | 1.56993 | Antisense | ENSG00000251393.3 | RP1-240K6.3 | XLOC_011068 | linc-NUMB | . |
| XLOC_023883 | chr14:77425975-77432557 | + | 0.442897 | 1.98968 | LincRNA | ENSG00000258602.1 | RP11-7F17.7 | XLOC_010881 | linc-KIAA1737-2 | . |
| XLOC_024029 | chr14:94959361-94964321 | + | 0.122587 | 1.7013 | Antisense | . | . | . | . | . |
| XLOC_024127 | chr14:101383059-101427505 | + | 15.2254 | 15.9263 | LincRNA | ENSG00000225746.4 | AL132709.5 | XLOC_010934 | linc-DIO3-6 | . |
| XLOC_024138 | chr14:101434993-101448689 | + | 63.0556 | 76.4738 | LincRNA | ENSG00000271417.1 | RP11-909M7.3 | . | . | . |
| XLOC_024142 | chr14:101473626-101499772 | + | 1.20775 | 1.93621 | LincRNA | . | . | . | . | . |
| XLOC_024151 | chr14:101519879-101526921 | + | 1.35262 | 1.91696 | LincRNA | . | . | . | . | . |
| XLOC_024348 | chr14:24408145-24423392 | - | 2.21478 | 1.24199 | Antisense | . | . | . | . | . |
| XLOC_024440 | chr14:35020973-35026463 | - | 1.28292 | 0.104883 | LincRNA | ENSG00000259135.1 | RP11-671J11.4 | . | . | . |
| XLOC_024451 | chr14:35385039-35451755 | - | 2.98658 | 1.47123 | Pseudogene | ENSG00000258493.2 | RP11-73E17.3 | . | . | . |
| XLOC_024483 | chr14:35862864-35867358 | - | 0.0337287 | 2.44857 | LincRNA | . | . | . | . | . |
| XLOC_024539 | chr14:42056652-42075504 | - | 0.900455 | 2.70922 | LincRNA | ENSG00000258636.1 | CTD-2298J14.2 | . | . | . |
| XLOC_024596 | chr14:50500383-50507672 | - | 0.650167 | 3.29792 | Pseudogene | ENSG00000270788.1 | PDLIM1P1 | . | . | . |
| XLOC_024712 | chr14:52649279-52655863 | - | 0.323122 | 1.03484 | LincRNA | . | . | . | . | . |
| XLOC_024761 | chr14:53490200-53620024 | - | 21.1233 | 21.9324 | Pseudogene | ENSG00000258993.1 | RP11-368P15.2 | . | . | . |
| XLOC_025014 | chr14:65620141-65637448 | - | 1.8995 | 6.98621 | LincRNA | . | . | . | . | eRNA |
| XLOC_025183 | chr14:71759465-71777388 | - | 2.17027 | 2.47198 | LincRNA | ENSG00000259146.2 | RP1-261D10.2 | XLOC_011064 | linc-MAP3K9-1 | . |
| XLOC_025239 | chr14:75079546-75083568 | - | 1.21382 | 1.0851 | LincRNA | ENSG00000258976.1 | CTD-2207P18.2 | . | . | . |
| XLOC_025659 | chr15:25264092-25267441 | + | 1.95216 | 1.318 | LincRNA | ENSG00000224078.8 | SNHG14 | XLOC_011183 | linc-GABRA5-7 | . |
| XLOC_025665 | chr15:25294163-25306886 | + | 1.14497 | 1.16821 | LincRNA | ENSG00000224078.8 | SNHG14 | . | . | . |
| XLOC_025669 | chr15:25309936-25351423 | + | 7.17509 | 6.1926 | LincRNA | ENSG00000224078.8 | SNHG14 | . | . | . |
| XLOC_025679 | chr15:25352633-25353187 | + | 6.83011 | 5.48723 | LincRNA | ENSG00000224078.8 | SNHG14 | . | . | . |
| XLOC_025680 | chr15:25354142-25366065 | + | 4.06522 | 3.93802 | LincRNA | ENSG00000224078.8 | SNHG14 | XLOC_011185 | linc-GABRA5-5 | . |
| XLOC_025730 | chr15:25756883-25803767 | + | 1.77324 | 1.73045 | LincRNA | ENSG00000235731.1 | AC124997.1 | XLOC_011405 | linc-UBE3A | . |
| XLOC_025794 | chr15:28912705-28914177 | + | 1.10857 | 0.872184 | Pseudogene | ENSG00000254398.1 | RP11-578F21.2 | . | . | . |
| XLOC_025801 | chr15:28982959-29004586 | + | 1.83868 | 1.84687 | Antisense | ENSG00000261480.1 | RP11-578F21.6 | . | . | . |
| XLOC_025813 | chr15:30395911-30402607 | + | 1.1037 | 0 | Pseudogene | ENSG00000178081.8 | ULK4P3 | . | . | . |
| XLOC_025957 | chr15:36003926-36150446 | + | 1.0569 | 0.163776 | LincRNA | ENSG00000248079.2 | DPH6-AS1 | . | . | . |
| XLOC_025981 | chr15:38656159-38665564 | + | 0.24804 | 1.40212 | LincRNA | . | . | . | . | . |
| XLOC_026112 | chr15:40331500-40352362 | + | 2.01395 | 2.27217 | LincRNA | ENSG00000248508.2 | SRP14-AS1 | XLOC_011219 | linc-BUB1B | . |
| XLOC_026156 | chr15:41576164-41598796 | + | 13.7025 | 11.5747 | LincRNA | ENSG00000247556.2 | OIP5-AS1 | XLOC_011448 | linc-EXD1 | . |
| XLOC_026242 | chr15:45825678-45848355 | + | 0.0859807 | 1.72905 | LincRNA | ENSG00000259354.1 | RP11-519G16.3 | . | . | . |
| XLOC_026700 | chr15:78190102-78194959 | + | 0.931848 | 1.37157 | Pseudogene | ENSG00000260139.2 | CSPG4P13 | . | . | . |
| XLOC_026755 | chr15:81474680-81614195 | + | 1.77964 | 0.220379 | Antisense | . | . | . | . | . |
| XLOC_026774 | chr15:83419321-83426033 | + | 2.68832 | 2.45575 | Antisense | ENSG00000250988.3 | RP11-752G15.6 | XLOC_011333 | linc-WHAMM-1 | . |
| XLOC_026812 | chr15:84749018-84773891 | + | 1.39921 | 1.02494 | Pseudogene | ENSG00000259404.1 | EFTUD1P1 | . | . | . |
| XLOC_026887 | chr15:89584278-89622914 | + | 1.90048 | 1.14021 | LincRNA | ENSG00000260123.1 | RP11-326A19.4 | XLOC_011344 | linc-ABHD2 | . |
| XLOC_026975 | chr15:100347865-100422749 | + | 11.3629 | 5.94215 | LincRNA | . | . | XLOC_011383 | linc-ASB7-4 | . |
| XLOC_027039 | chr15:102306219-102306677 | + | 1.44149 | 0.193086 | Pseudogene | ENSG00000259660.2 | DNM1P47 | . | . | . |
| XLOC_027049 | chr15:102515123-102516757 | + | 1.79372 | 1.81833 | Pseudogene | ENSG00000185596.12 | WASH3P | . | . | . |
| XLOC_027063 | chr15:20867317-20961425 | - | 0.442233 | 2.35798 | LincRNA | ENSG00000260409.1 | RP11-403B2.7 | . | . | . |
| XLOC_027094 | chr15:23185269-23212472 | - | 4.1573 | 5.14916 | Pseudogene | ENSG00000187667.6 | WHAMMP3 | . | . | . |
| XLOC_027099 | chr15:23281891-23295987 | - | 2.68125 | 2.30159 | Pseudogene | ENSG00000140181.10 | HERC2P2 | . | . | . |
| XLOC_027168 | chr15:32812777-32828866 | - | 5.83588 | 5.82092 | Pseudogene | ENSG00000223509.4 | RP11-632K20.7 | . | . | . |
| XLOC_027305 | chr15:38330295-38365459 | - | 0.0983273 | 1.94247 | LincRNA | ENSG00000259225.2 | RP11-1008C21.1 | XLOC_011213 | linc-SPRED1 | . |
| XLOC_027307 | chr15:38330295-38365459 | - | 0.53219 | 7.43766 | LincRNA | ENSG00000259225.2 | RP11-1008C21.1 | XLOC_011213 | linc-SPRED1 | . |
| XLOC_027362 | chr15:40985298-40987276 | - | 1.22414 | 1.0118 | Antisense | ENSG00000245849.2 | RAD51-AS1 | . | . | . |
| XLOC_027432 | chr15:44826662-44829071 | - | 5.48267 | 2.92891 | LincRNA | ENSG00000179523.4 | EIF3J-AS1 | . | . | . |
| XLOC_027700 | chr15:61927478-61945508 | - | 1.50911 | 0.497728 | LincRNA | ENSG00000259616.1 | RP11-507B12.2 | XLOC_011491 | linc-RORA | . |
| XLOC_027831 | chr15:67709270-67793136 | - | 1.42317 | 0.324459 | Antisense | ENSG00000259673.1 | IQCH-AS1 | . | . | . |
| XLOC_028110 | chr15:84954220-84957517 | - | 2.26847 | 0.122311 | Pseudogene | ENSG00000235370.5 | DNM1P51 | . | . | . |
| XLOC_028112 | chr15:85049565-85113762 | - | 6.56881 | 5.54066 | Pseudogene | ENSG00000259728.1 | LINC00933 | XLOC_011339 | linc-ZSCAN2-2 | . |
| XLOC_028232 | chr15:89339410-89342290 | - | 3.17848 | 3.41877 | LincRNA | ENSG00000259676.1 | RP11-343B18.2 | XLOC_011570 | linc-DET1-2 | . |
| XLOC_028403 | chr16:2014947-2015510 | + | 2.72196 | 2.22435 | LincRNA | ENSG00000255198.3 | SNHG9 | . | . | . |
| XLOC_028525 | chr16:14393350-14422671 | + | 1.80781 | 2.41523 | LincRNA | ENSG00000262454.1 | RP11-65J21.3 | XLOC_011650 | linc-BFAR | . |
| XLOC_028604 | chr16:21312463-21358608 | + | 1.70092 | 2.79615 | Pseudogene | ENSG00000257403.2 | CTD-2547E10.3 | . | . | . |
| XLOC_028694 | chr16:29097913-29128104 | + | 1.59769 | 1.14653 | Pseudogene | ENSG00000103472.5 | RRN3P2 | . | . | . |
| XLOC_028824 | chr16:31711899-31717488 | + | 1.42769 | 4.20848 | LincRNA | ENSG00000261731.2 | CTD-2358C21.4 | . | . | . |
| XLOC_028838 | chr16:31993259-31995463 | + | 5.97487 | 3.20768 | Pseudogene | ENSG00000260628.1 | RP11-1166P10.1 | . | . | . |
| XLOC_028931 | chr16:56651381-56652154 | + | 47.386 | 133.375 | Pseudogene | ENSG00000260549.1 | MT1L | . | . | . |
| XLOC_029455 | chr16:79635230-79639631 | + | 2.53536 | 1.78519 | LincRNA | . | . | . | . | . |
| XLOC_029461 | chr16:79747725-79781054 | + | 4.32071 | 1.62331 | LincRNA | ENSG00000261390.1 | RP11-345M22.2 | XLOC_011797 | linc-DYNLRB2-3 | . |
| XLOC_029615 | chr16:86715542-86730050 | + | 0.396287 | 1.08415 | LincRNA | . | . | XLOC_011815 | linc-MAP1LC3B-6 | . |
| XLOC_029677 | chr16:88729748-88741609 | + | 2.23942 | 0.801275 | LincRNA | ENSG00000260630.2 | SNAI3-AS1 | . | . | . |
| XLOC_029708 | chr16:90038990-90052087 | + | 2.15732 | 2.57212 | Pseudogene | ENSG00000223959.4 | AFG3L1P | . | . | . |
| XLOC_029715 | chr16:90056770-90063045 | + | 1.73687 | 1.3879 | Pseudogene | ENSG00000223959.4 | AFG3L1P | . | . | . |
| XLOC_029777 | chr16:2203370-2205360 | - | 1.56807 | 1.42544 | Antisense | ENSG00000260260.1 | RP11-304L19.5 | . | . | . |
| XLOC_029799 | chr16:3177432-3192775 | - | 4.19993 | 3.10569 | Antisense | ENSG00000263072.1 | RP11-473M20.14 | . | . | . |
| XLOC_029874 | chr16:10522758-10592329 | - | 0.818083 | 8.20796 | Antisense | . | . | . | . | . |
| XLOC_029978 | chr16:21495693-21531804 | - | 7.03098 | 5.35506 | Pseudogene | ENSG00000258186.2 | SLC7A5P2 | . | . | . |
| XLOC_029984 | chr16:21813869-21821304 | - | 1.89345 | 1.90019 | Pseudogene | ENSG00000248124.3 | RRN3P1 | . | . | . |
| XLOC_029985 | chr16:21821399-21825190 | - | 1.45293 | 1.37413 | Pseudogene | ENSG00000248124.3 | RRN3P1 | . | . | . |
| XLOC_029987 | chr16:21827738-21831762 | - | 2.16914 | 2.53109 | Pseudogene | ENSG00000248124.3 | RRN3P1 | . | . | . |
| XLOC_030020 | chr16:25114642-25122787 | - | 1.12149 | 0.349916 | LincRNA | ENSG00000260448.1 | RP11-449H11.1 | . | . | . |
| XLOC_030176 | chr16:33500666-33509663 | - | 1.32602 | 1.68098 | Pseudogene | ENSG00000260518.1 | BMS1P8 | . | . | . |
| XLOC_030312 | chr16:54952961-54963012 | - | 17.2893 | 19.8653 | LincRNA | ENSG00000245694.4 | CRNDE | XLOC_011950 | linc-IRX3-5 | . |
| XLOC_030453 | chr16:70010227-70048487 | - | 4.0201 | 4.70471 | Pseudogene | ENSG00000255185.1 | PDXDC2P | . | . | . |
| XLOC_030467 | chr16:70051950-70100044 | - | 5.16292 | 5.37833 | Pseudogene | ENSG00000255185.1 | PDXDC2P | . | . | . |
| XLOC_030478 | chr16:70247413-70258934 | - | 1.19795 | 0.753593 | Pseudogene | ENSG00000261556.4 | RP11-296I10.6 | XLOC_011997 | linc-NOB1 | . |
| XLOC_030516 | chr16:72318609-72425649 | - | 1.05996 | 0.749326 | LincRNA | . | . | XLOC_012003 | linc-PMFBP1-2 | . |
| XLOC_030548 | chr16:72574479-72698894 | - | 8.86419 | 3.81672 | LincRNA | ENSG00000261008.2 | AC004158.2 | . | . | . |
| XLOC_030573 | chr16:74358417-74402234 | - | 3.38267 | 2.52347 | LincRNA | ENSG00000259972.1 | AC009120.6 | . | . | . |
| XLOC_030791 | chr16:87098044-87099101 | - | 2.28041 | 0.979191 | LincRNA | . | . | . | . | . |
| XLOC_030803 | chr16:88210935-88220364 | - | 1.20078 | 1.65364 | LincRNA | ENSG00000260162.2 | RP11-863P13.1 | XLOC_011826 | linc-ZNF469-3 | . |
| XLOC_030913 | chr17:4891063-4894104 | + | 0.838932 | 1.07287 | Antisense | ENSG00000262227.1 | RP5-1050D4.5 | . | . | . |
| XLOC_030917 | chr17:5015219-5017670 | + | 3.88147 | 3.17776 | Antisense | ENSG00000234327.3 | AC012146.7 | . | . | . |
| XLOC_030918 | chr17:5095341-5126811 | + | 2.2469 | 0.481684 | Antisense | ENSG00000261879.1 | RP11-333E1.1 | . | . | . |
| XLOC_031059 | chr17:14285012-14326180 | + | 0.0952417 | 2.30413 | LincRNA | ENSG00000230647.1 | AC022816.2 | XLOC_012116 | linc-ZNF286A-6 | . |
| XLOC_031110 | chr17:16342173-16344913 | + | 201.547 | 207.254 | Antisense | ENSG00000175061.13 | C17orf76-AS1 | . | . | . |
| XLOC_031192 | chr17:20254246-20336381 | + | 2.88776 | 2.80348 | Pseudogene | ENSG00000154898.11 | CCDC144CP | . | . | . |
| XLOC_031283 | chr17:28927892-28964592 | + | 2.71815 | 2.33793 | Pseudogene | ENSG00000250462.4 | LRRC37BP1 | . | . | . |
| XLOC_031292 | chr17:29058984-29106781 | + | 3.20634 | 3.67669 | Pseudogene | ENSG00000264538.2 | SUZ12P | XLOC_012162 | linc-ATAD5-2 | . |
| XLOC_031315 | chr17:29335686-29386706 | + | 1.6225 | 0.836037 | Pseudogene | ENSG00000265798.2 | RP11-271K11.5 | . | . | . |
| XLOC_031342 | chr17:29956710-29965823 | + | 0.0949079 | 1.20545 | LincRNA | ENSG00000266877.1 | RP1-41C23.1 | XLOC_012166 | linc-SUZ12-3 | . |
| XLOC_031408 | chr17:33895072-33901400 | + | 2.0086 | 1.34694 | LincRNA | ENSG00000267321.1 | RP11-1094M14.11 | . | . | . |
| XLOC_031427 | chr17:35848670-35849330 | + | 0.358577 | 1.12454 | LincRNA | . | . | . | . | . |
| XLOC_031509 | chr17:40107297-40117727 | + | 1.32147 | 0.737208 | LincRNA | . | . | . | . | . |
| XLOC_031517 | chr17:40576908-40588410 | + | 2.33355 | 1.76819 | LincRNA | . | . | . | . | . |
| XLOC_031548 | chr17:41277613-41292983 | + | 2.03737 | 1.73344 | Pseudogene | ENSG00000267681.1 | CTD-3199J23.6 | . | . | . |
| XLOC_031876 | chr17:66097699-66129608 | + | 8.53594 | 6.48596 | Pseudogene | ENSG00000267023.1 | LRRC37A16P | . | . | . |
| XLOC_032007 | chr17:74553849-74562561 | + | 10.3274 | 14.1196 | Antisense | ENSG00000163597.10 | SNHG16 | . | . | . |
| XLOC_032065 | chr17:76840091-76843218 | + | 0.27879 | 1.31911 | Pseudogene | ENSG00000267355.2 | RPL9P29 | . | . | . |
| XLOC_032153 | chr17:6018-50202 | - | 1.17159 | 2.33501 | LincRNA | ENSG00000273172.1 | AC108004.2 | XLOC_012064 | linc-C17orf97-3 | . |
| XLOC_032200 | chr17:1607632-1619558 | - | 34.1506 | 65.7719 | LincRNA | ENSG00000186594.8 | MIR22HG | . | . | . |
| XLOC_032268 | chr17:6918075-6923151 | - | 1.9986 | 1.7479 | Antisense | ENSG00000262089.1 | RP11-589P10.5 | XLOC_012373 | linc-TEKT1-1 | . |
| XLOC_032312 | chr17:8123887-8126218 | - | 1.48214 | 1.51787 | LincRNA | ENSG00000178977.3 | LINC00324 | . | . | . |
| XLOC_032350 | chr17:10563455-10583143 | - | 1.32268 | 1.81144 | LincRNA | ENSG00000273290.1 | CTC-297N7.8 | . | . | . |
| XLOC_032516 | chr17:18414392-18499571 | - | 2.90244 | 2.58043 | Pseudogene | ENSG00000227077.2 | AC107983.4 | . | . | . |
| XLOC_032533 | chr17:18501941-18528939 | - | 8.22915 | 4.4326 | Pseudogene | ENSG00000227077.2 | AC107983.4 | . | . | . |
| XLOC_032934 | chr17:43351775-43394363 | - | 1.25453 | 0.621694 | LincRNA | . | . | . | . | . |
| XLOC_032969 | chr17:45095045-45134992 | - | 3.9071 | 2.54183 | Antisense | ENSG00000262879.1 | RP11-156P1.3 | . | . | . |
| XLOC_032985 | chr17:45148978-45177580 | - | 1.89719 | 1.40281 | LincRNA | ENSG00000262879.1 | RP11-156P1.3 | . | . | . |
| XLOC_033023 | chr17:45534147-45569881 | - | 1.26799 | 0.863539 | Pseudogene | ENSG00000228782.3 | MRPL45P2 | . | . | . |
| XLOC_033048 | chr17:45968185-45973637 | - | 1.67372 | 0.914883 | LincRNA | ENSG00000264920.1 | RP11-6N17.4 | . | . | . |
| XLOC_033049 | chr17:45973838-46018747 | - | 1.65343 | 1.25069 | Antisense | ENSG00000234494.3 | AC003665.1 | . | . | . |
| XLOC_033102 | chr17:48247501-48258586 | - | 1.36099 | 0.0622485 | Pseudogene | ENSG00000188662.5 | HILS1 | . | . | . |
| XLOC_033170 | chr17:55149421-55162411 | - | 1.8004 | 1.20073 | LincRNA | ENSG00000263004.1 | RP11-166P13.3 | XLOC_012530 | linc-COIL-2 | . |
| XLOC_033206 | chr17:58160894-58165757 | - | 5.04225 | 12.6891 | LincRNA | ENSG00000261040.2 | CTD-2319I12.1 | XLOC_012538 | linc-HEATR6-2 | . |
| XLOC_033261 | chr17:62745791-62749178 | - | 1.5724 | 2.07442 | Pseudogene | ENSG00000266820.1 | RP13-104F24.1 | . | . | . |
| XLOC_033262 | chr17:62750142-62771304 | - | 6.14733 | 6.88441 | Pseudogene | ENSG00000266820.1 | RP13-104F24.1 | . | . | . |
| XLOC_033268 | chr17:62776899-62833299 | - | 2.29653 | 2.55895 | Pseudogene | ENSG00000214176.5 | PLEKHM1P | . | . | . |
| XLOC_033305 | chr17:62964154-62971551 | - | 2.74821 | 2.43176 | Pseudogene | ENSG00000214174.4 | AMZ2P1 | . | . | . |
| XLOC_033343 | chr17:66686917-66691401 | - | 2.96227 | 11.0901 | LincRNA | ENSG00000267659.1 | RP11-118B18.1 | . | . | . |
| XLOC_033411 | chr17:70017943-70023288 | - | 2.98701 | 1.73143 | LincRNA | ENSG00000234899.5 | AC005152.2 | XLOC_012299 | linc-SOX9-2 | . |
| XLOC_033434 | chr17:70067191-70071807 | - | 1.48375 | 1.32324 | LincRNA | ENSG00000234899.5 | AC005152.2 | XLOC_012300 | linc-SOX9-1 | . |
| XLOC_033436 | chr17:70071857-70078996 | - | 2.27914 | 1.25598 | LincRNA | ENSG00000234899.5 | AC005152.2 | XLOC_012300 | linc-SOX9-1 | . |
| XLOC_033439 | chr17:70080048-70116925 | - | 8.99957 | 18.3499 | LincRNA | ENSG00000234899.5 | AC005152.2 | XLOC_012300 | linc-SOX9-1 | . |
| XLOC_033441 | chr17:70080048-70116925 | - | 1.03049 | 0.940165 | LincRNA | ENSG00000234899.5 | AC005152.2 | XLOC_012300 | linc-SOX9-1 | . |
| XLOC_033481 | chr17:70416093-70589000 | - | 1.2927 | 1.21055 | LincRNA | ENSG00000227036.2 | LINC00511 | XLOC_012568 | linc-ABCA5-3 | . |
| XLOC_033926 | chr18:3466229-3478952 | + | 1.34871 | 0.589663 | LincRNA | ENSG00000266835.1 | RP11-838N2.4 | XLOC_012747 | linc-MYOM1-2 | . |
| XLOC_033940 | chr18:5238078-5246501 | + | 4.6087 | 5.7811 | LincRNA | ENSG00000263753.2 | LINC00667 | . | . | . |
| XLOC_034112 | chr18:32870276-32897287 | + | 3.34464 | 3.48333 | Pseudogene | ENSG00000257267.1 | ZNF271 | . | . | . |
| XLOC_034184 | chr18:44790051-44810875 | + | 1.19758 | 1.383 | LincRNA | . | . | . | . | . |
| XLOC_034244 | chr18:53833409-53838003 | + | 0.0528565 | 2.20493 | LincRNA | ENSG00000267327.1 | CTD-2008L17.1 | XLOC_012687 | linc-WDR7-1 | . |
| XLOC_034491 | chr18:9318635-9334169 | - | 1.04633 | 0.565297 | LincRNA | ENSG00000264964.1 | RP11-888D10.3 | XLOC_012755 | linc-LAMA1-4 | . |
| XLOC_034506 | chr18:12039392-12048926 | - | 2.18602 | 1.03507 | Pseudogene | ENSG00000266995.1 | RP11-703I16.3 | . | . | . |
| XLOC_034872 | chr18:53865316-53893620 | - | 0.117971 | 1.71416 | LincRNA | . | . | . | . | . |
| XLOC_035007 | chr18:67332445-67392454 | - | 1.11129 | 4.03935 | Antisense | ENSG00000266840.1 | RP11-543H23.2 | . | . | . |
| XLOC_035100 | chr19:2328703-2339960 | + | 3.07109 | 3.20757 | LincRNA | . | . | . | . | . |
| XLOC_035102 | chr19:2340967-2345305 | + | 2.38528 | 0.378754 | LincRNA | . | . | . | . | . |
| XLOC_035107 | chr19:2351432-2353933 | + | 1.91069 | 1.4754 | LincRNA | ENSG00000273116.1 | LLfos-48D6.1 | . | . | . |
| XLOC_035144 | chr19:4909513-4941934 | + | 3.55426 | 2.5544 | LincRNA | . | . | . | . | . |
| XLOC_035292 | chr19:12098436-12146480 | + | 2.12233 | 4.15975 | Antisense | ENSG00000219665.4 | CTD-2006C1.2 | . | . | . |
| XLOC_035308 | chr19:12305858-12348222 | + | 0.774856 | 1.04578 | LincRNA | ENSG00000234773.2 | CTD-2666L21.1 | . | . | . |
| XLOC_035363 | chr19:14247855-14304723 | + | 0.76859 | 1.73585 | Antisense | ENSG00000267169.1 | CTB-55O6.12 | . | . | . |
| XLOC_035492 | chr19:20368525-20370009 | + | 7.1881 | 3.82365 | Pseudogene | ENSG00000269274.1 | AC078899.3 | . | . | . |
| XLOC_035554 | chr19:28284306-28301882 | + | 4.93245 | 3.89131 | Pseudogene | ENSG00000266906.1 | AC005357.1 | XLOC_013283 | linc-ZNF681-5 | . |
| XLOC_035733 | chr19:37288441-37341170 | + | 4.21545 | 4.04387 | LincRNA | ENSG00000267254.1 | CTD-2162K18.5 | . | . | . |
| XLOC_035770 | chr19:38307947-38323091 | + | 1.25979 | 0.998527 | LincRNA | ENSG00000225868.2 | AC016582.2 | . | . | . |
| XLOC_035852 | chr19:42901314-42912856 | + | 2.30681 | 1.22657 | Antisense | ENSG00000213904.4 | LIPE-AS1 | . | . | . |
| XLOC_035913 | chr19:44694701-44703104 | + | 1.04741 | 0.639881 | LincRNA | . | . | XLOC_013093 | linc-ZNF227 | . |
| XLOC_036064 | chr19:52197578-52207092 | + | 0.985914 | 1.34876 | LincRNA | ENSG00000269959.1 | hsa-mir-125a | . | . | . |
| XLOC_036109 | chr19:53700353-53719695 | + | 2.53007 | 1.71251 | LincRNA | ENSG00000269051.1 | CTD-2245F17.3 | . | . | . |
| XLOC_036169 | chr19:56879602-56891393 | + | 6.26391 | 5.07099 | Pseudogene | ENSG00000240225.6 | ZNF542 | . | . | . |
| XLOC_036176 | chr19:56989418-57007841 | + | 2.78082 | 1.96261 | Antisense | ENSG00000166770.6 | ZNF667-AS1 | . | . | . |
| XLOC_036239 | chr19:58892833-58897869 | + | 2.05781 | 1.70791 | LincRNA | . | . | . | . | . |
| XLOC_036264 | chr19:59086685-59116602 | + | 2.8619 | 2.75133 | Pseudogene | ENSG00000213753.6 | RPL23AP79 | . | . | . |
| XLOC_036475 | chr19:10762539-10764544 | - | 2.03223 | 1.54501 | LincRNA | ENSG00000267100.1 | ILF3-AS1 | XLOC_013222 | linc-AP1M2 | . |
| XLOC_036583 | chr19:17596874-17632097 | - | 0.963841 | 1.50464 | Antisense | ENSG00000269439.1 | CTD-3131K8.2 | . | . | . |
| XLOC_036700 | chr19:23239345-23448008 | - | 0.998866 | 1.9797 | LincRNA | ENSG00000213971.3 | RP11-15H20.6 | XLOC_013018 | linc-ZNF726-5 | . |
| XLOC_036715 | chr19:27731916-27740280 | - | 13.0123 | 10.3394 | LincRNA | . | . | . | . | . |
| XLOC_036743 | chr19:28258403-28268784 | - | 2.05478 | 1.50219 | Pseudogene | ENSG00000266906.1 | AC005357.1 | XLOC_013283 | linc-ZNF681-5 | . |
| XLOC_036766 | chr19:32880730-32896443 | - | 0.660716 | 1.11252 | Antisense | ENSG00000267213.3 | AC007773.2 | XLOC_013300 | linc-TSHZ3-6 | . |
| XLOC_036828 | chr19:36031201-36036418 | - | 1.3762 | 0.879627 | Antisense | ENSG00000236144.2 | AD000090.2 | . | . | . |
| XLOC_037412 | chr1_gl000192_random:354710-397660 | - | 1.39512 | 0.554967 | LincRNA | . | . | . | . | . |
| XLOC_037459 | chr2:3606099-3610115 | + | 1.99677 | 2.28757 | Antisense | ENSG00000234171.1 | AC108488.3 | XLOC_001323 | linc-RPS7-1 | . |
| XLOC_037680 | chr2:19100476-19251982 | + | 1.30023 | 1.1848 | LincRNA | ENSG00000236204.1 | AC092594.1 | XLOC_001374 | linc-RHOB-7 | . |
| XLOC_037732 | chr2:25194985-25202221 | + | 2.20843 | 1.10879 | LincRNA | ENSG00000224165.1 | DNAJC27-AS1 | XLOC_001398 | linc-EFR3B | . |
| XLOC_037837 | chr2:30569482-30582371 | + | 1.83506 | 1.16067 | LincRNA | ENSG00000235997.2 | AC109642.1 | XLOC_001412 | linc-LCLAT1-1 | . |
| XLOC_037958 | chr2:39664480-39827363 | + | 3.50573 | 2.42173 | LincRNA | ENSG00000231312.2 | AC007246.3 | . | . | . |
| XLOC_038048 | chr2:46871684-46879692 | + | 1.15962 | 0.889644 | LincRNA | . | . | XLOC_001457 | linc-SOCS5 | . |
| XLOC_038238 | chr2:65710010-65919669 | + | 0.774758 | 1.0662 | LincRNA | ENSG00000234255.4 | AC012370.3 | XLOC_002135 | linc-SPRED2-3 | . |
| XLOC_038665 | chr2:87754303-87883362 | + | 54.2394 | 70.0468 | LincRNA | ENSG00000222041.6 | LINC00152 | . | . | . |
| XLOC_038735 | chr2:95873143-95888872 | + | 1.56552 | 0.731502 | LincRNA | ENSG00000233757.2 | AC092835.2 | . | . | . |
| XLOC_038805 | chr2:98289789-98319989 | + | 0.909248 | 2.42461 | LincRNA | ENSG00000228486.5 | AC017099.3 | . | . | . |
| XLOC_038899 | chr2:107103525-107125909 | + | 6.03897 | 5.70801 | Pseudogene | ENSG00000254126.2 | CD8BP | . | . | . |
| XLOC_039211 | chr2:113993023-114034254 | + | 3.8645 | 5.9968 | Antisense | ENSG00000258395.1 | RP11-65I12.1 | . | . | . |
| XLOC_039267 | chr2:114341679-114357851 | + | 0.92219 | 1.04209 | Pseudogene | ENSG00000236397.2 | DDX11L2 | . | . | . |
| XLOC_039395 | chr2:128145785-128147876 | + | 1.47101 | 1.59323 | Antisense | ENSG00000236682.1 | AC068282.3 | . | . | . |
| XLOC_039406 | chr2:128619205-128651349 | + | 2.46451 | 3.8502 | LincRNA | . | . | . | . | . |
| XLOC_039452 | chr2:132271398-132279126 | + | 1.14998 | 0.802598 | Pseudogene | ENSG00000152117.13 | AC093838.4 | . | . | . |
| XLOC_039546 | chr2:145780653-145942522 | + | 2.87056 | 2.68689 | LincRNA | ENSG00000226674.4 | TEX41 | . | . | . |
| XLOC_039822 | chr2:162931381-162934450 | + | 0.780231 | 1.00628 | LincRNA | ENSG00000230918.1 | AC008063.2 | . | . | . |
| XLOC_039823 | chr2:162949700-162951548 | + | 0.686334 | 1.13505 | LincRNA | ENSG00000233397.1 | AC008063.3 | XLOC_001731 | linc-GCA-2 | . |
| XLOC_039849 | chr2:168149496-168730549 | + | 3.00436 | 3.5352 | LincRNA | ENSG00000228222.1 | AC074363.1 | XLOC_001742 | linc-B3GALT1 | . |
| XLOC_040162 | chr2:171649386-171655588 | + | 1.15225 | 0.946131 | LincRNA | ENSG00000239467.1 | AC007405.6 | . | . | . |
| XLOC_040301 | chr2:179278401-179315400 | + | 1.71795 | 1.21274 | Antisense | ENSG00000223960.2 | AC009948.5 | . | . | . |
| XLOC_040330 | chr2:179459826-179496198 | + | 2.84518 | 2.28809 | Antisense | ENSG00000237298.4 | TTN-AS1 | . | . | . |
| XLOC_040700 | chr2:191399622-191435539 | + | 1.14376 | 1.01711 | LincRNA | ENSG00000233654.1 | AC093388.3 | . | . | . |
| XLOC_040757 | chr2:196603472-196622911 | + | 1.05294 | 0.774732 | Antisense | . | . | . | . | . |
| XLOC_040838 | chr2:202835756-202845480 | + | 0 | 1.04083 | LincRNA | . | . | . | . | . |
| XLOC_040907 | chr2:207661812-207734815 | + | 3.42514 | 3.78119 | LincRNA | ENSG00000229321.1 | AC008269.2 | XLOC_001822 | linc-CPO-2 | . |
| XLOC_040939 | chr2:207743510-207791470 | + | 1.20627 | 1.41609 | LincRNA | . | . | XLOC_001823 | linc-CPO-1 | . |
| XLOC_040968 | chr2:208104033-208113615 | + | 0.287034 | 1.26093 | LincRNA | ENSG00000223725.2 | AC007879.5 | XLOC_002473 | linc-KLF7-2 | . |
| XLOC_041112 | chr2:218142867-218160680 | + | 0.817261 | 2.9568 | Antisense | ENSG00000233143.1 | AC009492.1 | XLOC_001864 | linc-RUFY4-2 | . |
| XLOC_041210 | chr2:225016338-225035395 | + | 0.15341 | 1.18994 | Pseudogene | ENSG00000224826.1 | AC019109.1 | . | . | . |
| XLOC_041224 | chr2:225102634-225115967 | + | 0.0149379 | 2.29395 | LincRNA | . | . | . | . | . |
| XLOC_041304 | chr2:228520421-228523892 | + | 0.180699 | 3.84837 | LincRNA | . | . | . | . | . |
| XLOC_041470 | chr2:242626493-242634156 | + | 3.03725 | 1.87061 | LincRNA | ENSG00000228989.1 | AC133528.2 | . | . | . |
| XLOC_041483 | chr2:243030830-243061941 | + | 2.4471 | 1.31407 | Pseudogene | ENSG00000220804.4 | AC093642.5 | . | . | . |
| XLOC_041501 | chr2:243064101-243101572 | + | 1.28412 | 0.700854 | Pseudogene | ENSG00000220804.4 | AC093642.5 | . | . | . |
| XLOC_041552 | chr2:8142787-8144459 | - | 0.0299976 | 1.67768 | LincRNA | ENSG00000236790.1 | LINC00299 | XLOC_001332 | linc-ID2-2 | . |
| XLOC_042001 | chr2:36580757-36582747 | - | 2.39798 | 1.65857 | LincRNA | ENSG00000260025.1 | RP11-490M8.1 | XLOC_002048 | linc-FAM98A-3 | . |
| XLOC_042034 | chr2:38421248-38468349 | - | 7.77557 | 11.6519 | LincRNA | ENSG00000227292.1 | AC009229.5 | . | . | . |
| XLOC_042424 | chr2:64989523-64995661 | - | 0.244116 | 1.46934 | LincRNA | . | . | . | . | . |
| XLOC_042586 | chr2:70257040-70313601 | - | 4.15442 | 3.71593 | LincRNA | ENSG00000179818.9 | PCBP1-AS1 | . | . | . |
| XLOC_042660 | chr2:74153943-74209862 | - | 2.17985 | 4.84241 | Antisense | ENSG00000237883.1 | DGUOK-AS1 | . | . | . |
| XLOC_042725 | chr2:81883247-81885379 | - | 0.471744 | 2.22109 | LincRNA | . | . | . | . | . |
| XLOC_042748 | chr2:85614050-85619333 | - | 1.57392 | 1.05859 | Antisense | ENSG00000273196.1 | RP11-717A5.2 | . | . | . |
| XLOC_042930 | chr2:98163528-98167878 | - | 3.1025 | 3.24435 | Pseudogene | ENSG00000196912.8 | ANKRD36B | . | . | . |
| XLOC_042931 | chr2:98167944-98206744 | - | 4.40218 | 5.7372 | Pseudogene | ENSG00000196912.8 | ANKRD36B | . | . | . |
| XLOC_043002 | chr2:101339479-101359483 | - | 0.0800317 | 1.47531 | LincRNA | . | . | XLOC_001591 | linc-NPAS2-1 | . |
| XLOC_043077 | chr6:143267747-143280112 |  | 0.959518 | 59.511 | LincRNA | ENSG00000232618.1 | RP11-439L18.1 | - | - | . |
| XLOC_043152 | chr2:108943626-108970643 | - | 3.12128 | 1.81349 | Pseudogene | ENSG00000237223.2 | SULT1C2P1 | . | . | . |
| XLOC_043199 | chr2:110969103-110970123 | - | 17.7547 | 20.3968 | LincRNA | ENSG00000175701.6 | LINC00116 | XLOC_002263 | linc-NPHP1-3 | . |
| XLOC_043218 | chr2:111963612-112252629 | - | 41.1609 | 52.2653 | LincRNA | ENSG00000172965.10 | MIR4435-1HG | XLOC_001625 | linc-MERTK-2 | . |
| XLOC_043318 | chr2:114363031-114384745 | - | 4.82575 | 3.67137 | Pseudogene | ENSG00000240356.2 | RPL23AP7 | . | . | . |
| XLOC_043336 | chr2:114632728-114641302 | - | 1.74732 | 0.737257 | LincRNA | . | . | . | . | . |
| XLOC_043456 | chr2:135624654-135676255 | - | 1.24219 | 0.624214 | Antisense | ENSG00000224043.3 | AC016725.4 | . | . | . |
| XLOC_043678 | chr2:151560131-151567657 | - | 0.17628 | 3.03823 | LincRNA | . | . | . | . | . |
| XLOC_043698 | chr2:152127077-152196200 | - | 0.39188 | 10.9272 | LincRNA | . | . | . | . | . |
| XLOC_044103 | chr2:177494329-177502603 | - | 5.56593 | 4.68787 | LincRNA | ENSG00000224577.1 | AC017048.4 | XLOC_002408 | linc-EVX2-8 | . |
| XLOC_044279 | chr2:186898388-187173382 | - | 3.3574 | 3.83106 | LincRNA | ENSG00000237877.2 | AC097500.2 | XLOC_001781 | linc-ZC3H15 | . |
| XLOC_044481 | chr2:189757751-189765861 | - | 0.0134959 | 1.51344 | LincRNA | . | . | . | . | . |
| XLOC_044540 | chr2:190147030-190203182 | - | 1.72835 | 0.922382 | Pseudogene | ENSG00000213601.3 | KRT18P19 | XLOC_002433 | linc-COL5A2 | . |
| XLOC_044575 | chr2:190252753-190260012 | - | 1.05366 | 0.259826 | LincRNA | . | . | XLOC_002433 | linc-COL5A2 | . |
| XLOC_044989 | chr2:216601201-216642209 | - | 1.62504 | 0.909054 | LincRNA | ENSG00000235770.1 | LINC00607 | . | . | . |
| XLOC_045024 | chr2:216706938-216708800 | - | 1.72881 | 0.836894 | LincRNA | ENSG00000235770.1 | LINC00607 | . | . | . |
| XLOC_045044 | chr2:216781666-216807071 | - | 1.13632 | 1.07807 | LincRNA | ENSG00000226276.1 | AC093382.1 | . | . | . |
| XLOC_045202 | chr2:228132458-228170366 | - | 1.13458 | 0.279691 | Antisense | ENSG00000236432.3 | AC097662.2 | . | . | . |
| XLOC_045330 | chr2:232371935-232379098 | - | 1.42231 | 0.875137 | LincRNA | . | . | . | . | . |
| XLOC_045353 | chr2:234774050-234777051 | - | 1.79592 | 0.714469 | Pseudogene | ENSG00000224287.2 | MSL3P1 | . | . | . |
| XLOC_045798 | chr20:18548051-18550202 | + | 18.2563 | 22.1203 | LincRNA | ENSG00000232388.2 | LINC00493 | . | . | . |
| XLOC_045852 | chr20:21106643-21227260 | + | 9.43855 | 7.30602 | LincRNA | ENSG00000232712.2 | RP4-777D9.2 | . | . | . |
| XLOC_045876 | chr20:22634093-22666209 | + | 1.12267 | 0.0198333 | LincRNA | ENSG00000230400.1 | RP11-359G22.2 | XLOC_013486 | linc-SSTR4-3 | . |
| XLOC_045918 | chr20:25677644-25716812 | + | 1.47072 | 0.530997 | Pseudogene | ENSG00000230772.1 | VN1R108P | . | . | . |
| XLOC_045941 | chr20:25731841-25733380 | + | 2.52219 | 1.00544 | LincRNA | ENSG00000226465.1 | RP13-401N8.1 | XLOC_013708 | linc-ZNF337-10 | . |
| XLOC_046094 | chr20:37075328-37079566 | + | 2.42789 | 1.76678 | LincRNA | ENSG00000174365.15 | SNHG11 | XLOC_013520 | linc-RALGAPB | . |
| XLOC_046137 | chr20:42839616-42854812 | + | 1.3922 | 1.58927 | LincRNA | ENSG00000223891.1 | OSER1-AS1 | . | . | . |
| XLOC_046187 | chr20:46653889-46680019 | + | 1.96763 | 0.827303 | LincRNA | . | . | XLOC_013547 | linc-ARFGEF2-8 | . |
| XLOC_046198 | chr20:46680131-46687285 | + | 1.25106 | 0.923008 | LincRNA | . | . | XLOC_013547 | linc-ARFGEF2-8 | . |
| XLOC_046219 | chr20:47895142-47905765 | + | 14.9642 | 13.0781 | LincRNA | ENSG00000177410.8 | ZFAS1 | . | . | . |
| XLOC_046269 | chr20:49962279-49986371 | + | 0.336183 | 1.15407 | LincRNA | . | . | . | . | . |
| XLOC_046315 | chr20:57090527-57148810 | + | 1.2356 | 2.6278 | LincRNA | ENSG00000254620.1 | RP5-907D15.3 | . | . | . |
| XLOC_046358 | chr20:58713521-58729661 | + | 1.71153 | 0.959479 | LincRNA | ENSG00000228340.1 | RP5-1043L13.1 | . | . | . |
| XLOC_046446 | chr20:1033422-1039924 | - | 3.0714 | 0 | LincRNA | . | . | . | . | . |
| XLOC_046503 | chr20:1786361-1790866 | - | 0.674102 | 1.0557 | LincRNA | ENSG00000230839.1 | RP5-968J1.1 | . | . | . |
| XLOC_046524 | chr20:1868257-1880764 | - | 2.86369 | 3.34772 | LincRNA | . | . | . | . | . |
| XLOC_046603 | chr20:5477728-5485151 | - | 1.24533 | 0.68923 | LincRNA | ENSG00000205181.5 | LINC00654 | XLOC_013449 | linc-C20orf196-1 | . |
| XLOC_046615 | chr20:6746592-6747267 | - | 1.52675 | 0.861066 | LincRNA | . | . | . | . | . |
| XLOC_046798 | chr20:22576660-22588286 | - | 1.48161 | 0.133054 | LincRNA | ENSG00000237396.1 | RP4-788L20.3 | XLOC_013685 | linc-FOXA2-2 | . |
| XLOC_046931 | chr20:34192486-34194455 | - | 0.159221 | 1.14236 | Pseudogene | ENSG00000088340.11 | FER1L4 | . | . | . |
| XLOC_046975 | chr20:37046782-37059159 | - | 1.169 | 2.04028 | LincRNA | ENSG00000235408.2 | SNORA71B | . | . | . |
| XLOC_046976 | chr20:37059238-37063381 | - | 0.761621 | 1.75497 | LincRNA | ENSG00000235408.2 | SNORA71B | XLOC_013739 | linc-KIAA1755-4 | . |
| XLOC_047243 | chr20:61193628-61200537 | - | 0.478033 | 1.74351 | LincRNA | . | . | XLOC_013602 | linc-SLCO4A1-2 | . |
| XLOC_047341 | chr21:15399766-15516779 | + | 3.01615 | 2.67554 | Antisense | ENSG00000224905.2 | AP001347.6 | . | . | . |
| XLOC_047414 | chr21:17300556-17335218 | + | 1.41349 | 0.386129 | LincRNA | . | . | . | . | . |
| XLOC_047732 | chr21:17999826-18006264 | + | 2.8288 | 0.627205 | LincRNA | ENSG00000228798.1 | AP000473.5 | . | . | . |
| XLOC_047794 | chr21:26934114-26976653 | + | 0.266717 | 13.3313 | Antisense | ENSG00000260583.1 | LINC00515 | XLOC_014038 | linc-TMPRSS15-16 | . |
| XLOC_047906 | chr21:35541900-35564339 | + | 1.0203 | 0.471983 | LincRNA | ENSG00000227456.3 | LINC00310 | XLOC_013923 | linc-KCNE2-3 | . |
| XLOC_047942 | chr21:35665985-35697214 | + | 0.903422 | 4.75285 | LincRNA | ENSG00000214955.5 | AP000318.2 | . | . | . |
| XLOC_047962 | chr21:36118019-36156944 | + | 0.883049 | 1.9054 | LincRNA | ENSG00000234380.1 | AP000330.8 | XLOC_013928 | linc-CBR1-2 | . |
| XLOC_048004 | chr21:37817942-37870038 | + | 2.43264 | 0.540231 | Pseudogene | ENSG00000223741.1 | PSMD4P1 | . | . | . |
| XLOC_048072 | chr21:43188194-43194760 | + | 0.23699 | 3.07771 | LincRNA | . | . | XLOC_013950 | linc-UMODL1-2 | eRNA |
| XLOC_048078 | chr21:43406832-43431780 | + | 2.15377 | 9.52715 | Antisense | ENSG00000237232.3 | ZNF295-AS1 | . | . | . |
| XLOC_048141 | chr21:46269498-46300233 | + | 0.614433 | 23.6698 | Antisense | . | . | . | . | . |
| XLOC_048151 | chr21:46707988-46713790 | + | 4.58793 | 3.08554 | LincRNA | ENSG00000215447.3 | BX322557.10 | XLOC_013981 | linc-COL18A1-4 | . |
| XLOC_048345 | chr21:26796229-26825140 | - | 0.144383 | 2.3311 | LincRNA | ENSG00000185433.4 | LINC00158 | . | . | . |
| XLOC_048423 | chr21:28984539-29019990 | - | 0.0718199 | 2.23055 | LincRNA | ENSG00000234052.1 | AP001607.1 | XLOC_013896 | linc-USP16-9 | . |
| XLOC_048519 | chr21:29812591-30047487 | - | 11.447 | 9.81988 | LincRNA | ENSG00000226935.2 | LINC00161 | XLOC_013901 | linc-USP16-4 | . |
| XLOC_048719 | chr21:36103222-36107256 | - | 0.345082 | 1.00174 | LincRNA | ENSG00000230978.1 | LINC00160 | XLOC_013927 | linc-CBR1-3 | . |
| XLOC_048827 | chr21:43470814-43475824 | - | 0.255665 | 3.23699 | LincRNA | . | . | XLOC_013952 | linc-UMODL1-1 | . |
| XLOC_048882 | chr21:46419024-46424480 | - | 1.68444 | 1.70244 | LincRNA | ENSG00000224930.2 | LINC00162 | XLOC_014121 | linc-ITGB2-3 | . |
| XLOC_048934 | chr22:17528285-17542463 | + | 1.327 | 0.519363 | LincRNA | ENSG00000237438.2 | CECR7 | . | . | . |
| XLOC_049012 | chr22:22652434-22673571 | + | 18.8886 | 21.3918 | Pseudogene | ENSG00000272779.1 | LL22NC03-80A10.6 | . | . | . |
| XLOC_049020 | chr22:22675766-22677365 | + | 0.829856 | 1.05694 | LincRNA | . | . | . | . | . |
| XLOC_049054 | chr22:25817227-26120781 | + | 2.33469 | 1.91598 | Pseudogene | ENSG00000100058.8 | CRYBB2P1 | XLOC_014323 | linc-LRP5L-1 | . |
| XLOC_049091 | chr22:26908481-26910704 | + | 1.15271 | 0.938483 | LincRNA | ENSG00000261188.1 | CTA-445C9.14 | . | . | . |
| XLOC_049092 | chr22:27068758-27176185 | + | 3.5376 | 5.33304 | LincRNA | ENSG00000225783.2 | MIAT | XLOC_014327 | linc-CRYBB1-1 | . |
| XLOC_049188 | chr22:29190552-29444480 | + | 1.60415 | 1.87794 | LincRNA | ENSG00000226471.2 | CTA-292E10.6 | XLOC_014334 | linc-XBP1 | . |
| XLOC_049219 | chr22:31365300-31374989 | + | 18.3021 | 18.0237 | LincRNA | ENSG00000253352.4 | TUG1 | XLOC_014209 | linc-SMTN | . |
| XLOC_049488 | chr22:51222179-51239725 | + | 10.3598 | 8.16756 | Pseudogene | ENSG00000184319.11 | AC002055.4 | . | . | . |
| XLOC_049569 | chr22:24001830-24034133 | - | 4.65145 | 3.05676 | Pseudogene | ENSG00000272578.1 | AP000347.2 | XLOC_014177 | linc-RGL4-1 | . |
| XLOC_049575 | chr22:24034240-24059515 | - | 1.75395 | 1.19428 | Pseudogene | ENSG00000272578.1 | AP000347.2 | . | . | . |
| XLOC_049646 | chr22:30608614-30609027 | - | 0.104598 | 1.95953 | LincRNA | . | . | . | . | . |
| XLOC_049693 | chr22:35588412-35627034 | - | 0 | 1.45129 | Pseudogene | ENSG00000243453.1 | COX7BP1 | . | . | . |
| XLOC_049702 | chr22:36526651-36529733 | - | 0.168523 | 1.06295 | LincRNA | . | . | . | . | . |
| XLOC_049867 | chr22:45529624-45559769 | - | 3.06604 | 3.52648 | LincRNA | ENSG00000226328.2 | CTA-217C2.1 | XLOC_014394 | linc-PHF21B | . |
| XLOC_050403 | chr3:23710334-23711913 | + | 0.0888147 | 1.59485 | LincRNA | . | . | . | . | . |
| XLOC_050466 | chr3:27674249-27689076 | + | 37.6944 | 31.5503 | LincRNA | ENSG00000271943.1 | RP11-222K16.1 | . | . | . |
| XLOC_050548 | chr3:28798613-28799613 | + | 1.22897 | 0.364047 | LincRNA | ENSG00000228214.2 | LINC00693 | . | . | . |
| XLOC_050708 | chr3:40807595-40952516 | + | 0.22957 | 1.79425 | LincRNA | ENSG00000231873.1 | RP11-761N21.1 | XLOC_002622 | linc-CTNNB1-2 | . |
| XLOC_050764 | chr3:40807595-40952516 | + | 0.381927 | 1.36133 | LincRNA | . | . | XLOC_002622 | linc-CTNNB1-2 | . |
| XLOC_050803 | chr3:44040801-44164067 | + | 1.88309 | 1.07735 | LincRNA | ENSG00000261786.1 | RP4-555D20.2 | XLOC_002629 | linc-C3orf77 | . |
| XLOC_050908 | chr3:47192854-47217774 | + | 1.09171 | 0.874661 | LincRNA | ENSG00000227398.3 | KIF9-AS1 | XLOC_002636 | linc-KLHL18-3 | . |
| XLOC_050988 | chr3:50242703-50251944 | + | 1.56247 | 0.0675862 | LincRNA | . | . | . | . | . |
| XLOC_050994 | chr3:50305078-50313282 | + | 3.77102 | 3.86641 | LincRNA | . | . | . | . | . |
| XLOC_051105 | chr3:64608107-64996819 | + | 2.74289 | 1.97071 | LincRNA | ENSG00000241684.1 | ADAMTS9-AS2 | XLOC_003149 | linc-ADAMTS9-2 | . |
| XLOC_051296 | chr3:67224330-67226702 | + | 0.186309 | 1.2488 | LincRNA | . | . | . | . | . |
| XLOC_051300 | chr3:67705134-67891203 | + | 4.30003 | 2.97275 | LincRNA | ENSG00000241316.2 | RP11-81N13.1 | XLOC_002693 | linc-FAM19A1-1 | . |
| XLOC_051428 | chr3:73871646-73929434 | + | 0.039967 | 1.32436 | LincRNA | ENSG00000242741.1 | RP11-20B7.1 | XLOC_002705 | linc-FRG2C-8 | . |
| XLOC_051626 | chr3:106959534-106968104 | + | 2.33536 | 1.3021 | LincRNA | ENSG00000243701.1 | LINC00883 | XLOC_003206 | linc-CBLB-7 | . |
| XLOC_051993 | chr3:125932944-125966855 | + | 1.52308 | 0.315797 | LincRNA | . | . | XLOC_002796 | linc-CCDC37-4 | . |
| XLOC_052006 | chr3:125985672-126010335 | + | 1.78676 | 0.30563 | LincRNA | ENSG00000250934.1 | RP11-71E19.1 | XLOC_002798 | linc-CCDC37-2 | . |
| XLOC_052162 | chr3:139108632-139396803 | + | 2.9774 | 2.40143 | Pseudogene | ENSG00000214280.3 | RP11-553K23.2 | . | . | . |
| XLOC_052429 | chr3:152203323-152205115 | + | 1.27059 | 0.259236 | LincRNA | ENSG00000243305.1 | RP11-362A9.3 | . | . | . |
| XLOC_052515 | chr3:156804948-156806490 | + | 0.615084 | 1.12429 | LincRNA | ENSG00000241135.1 | LINC00881 | XLOC_002892 | linc-PTX3-4 | . |
| XLOC_052526 | chr3:156892905-157033993 | + | 3.47039 | 3.2144 | LincRNA | ENSG00000243176.1 | RP11-550I24.2 | . | . | . |
| XLOC_052774 | chr3:177159527-177470284 | + | 0.588609 | 1.2006 | LincRNA | ENSG00000228221.1 | LINC00578 | XLOC_002931 | linc-KCNMB2-5 | eRNA |
| XLOC_052807 | chr3:177483416-177484716 | + | 0.079183 | 1.54077 | LincRNA | . | . | XLOC_002932 | linc-KCNMB2-3 | . |
| XLOC_052809 | chr3:177545591-177615946 | + | 1.60532 | 5.35371 | LincRNA | ENSG00000231574.1 | RP11-91K9.1 | XLOC_002934 | linc-KCNMB2-2 | . |
| XLOC_052931 | chr3:183165416-183173796 | + | 9.57208 | 9.19109 | Pseudogene | ENSG00000240024.1 | LINC00888 | . | . | . |
| XLOC_052940 | chr3:184432882-184458213 | + | 1.38924 | 0.559581 | LincRNA | ENSG00000272970.1 | RP11-329B9.4 | XLOC_002951 | linc-VPS8-3 | . |
| XLOC_052969 | chr3:185677739-185689876 | + | 4.35945 | 6.78991 | Pseudogene | ENSG00000171658.4 | RP11-443P15.2 | . | . | . |
| XLOC_052975 | chr3:185691693-185698799 | + | 2.97524 | 2.92137 | Pseudogene | ENSG00000171658.4 | RP11-443P15.2 | . | . | . |
| XLOC_053455 | chr3:195390722-195393045 | + | 1.39234 | 3.43562 | LincRNA | ENSG00000242086.4 | LINC00969 | . | . | . |
| XLOC_053458 | chr3:195400411-195414925 | + | 1.97049 | 1.6734 | LincRNA | ENSG00000242086.4 | LINC00969 | . | . | . |
| XLOC_053466 | chr3:195415169-195438831 | + | 10.0775 | 11.8102 | LincRNA | ENSG00000242086.4 | LINC00969 | XLOC_002997 | linc-MUC20-1 | . |
| XLOC_053634 | chr3:9404663-9529762 | - | 3.27253 | 5.85872 | Antisense | ENSG00000206573.4 | SETD5-AS1 | . | . | . |
| XLOC_053696 | chr3:12953725-13205629 | - | 2.14454 | 1.88682 | LincRNA | . | . | . | . | eRNA |
| XLOC_053741 | chr3:14984557-15106835 | - | 27.6199 | 30.1888 | LincRNA | ENSG00000225733.1 | FGD5-AS1 | . | . | . |
| XLOC_054165 | chr3:39942101-40216035 | - | 2.58882 | 2.3921 | Antisense | . | . | . | . | . |
| XLOC_054201 | chr3:40338409-40351031 | - | 0.947267 | 2.46003 | LincRNA | . | . | . | . | . |
| XLOC_054326 | chr3:46556814-46648554 | - | 13.8461 | 5.42389 | Antisense | . | . | . | . | . |
| XLOC_054490 | chr3:62180737-62303523 | - | 2.12109 | 0.565457 | LincRNA | ENSG00000241472.2 | PTPRG-AS1 | . | . | . |
| XLOC_054647 | chr3:75475600-75484312 | - | 2.40611 | 2.11619 | Pseudogene | ENSG00000244026.2 | FAM86DP | . | . | . |
| XLOC_054958 | chr3:106991577-107140189 | - | 1.98427 | 1.01512 | LincRNA | ENSG00000243701.1 | LINC00883 | XLOC_003206 | linc-CBLB-7 | . |
| XLOC_055218 | chr3:125546076-125567418 | - | 1.44325 | 1.06911 | Pseudogene | ENSG00000241278.1 | ENPP7P4 | . | . | . |
| XLOC_055240 | chr3:125590880-125609177 | - | 1.13003 | 1.26088 | Pseudogene | ENSG00000241278.1 | ENPP7P4 | . | . | . |
| XLOC_055252 | chr3:125626012-125634617 | - | 2.23516 | 1.65045 | Pseudogene | ENSG00000241278.1 | ENPP7P4 | . | . | . |
| XLOC_055309 | chr3:128592200-128598269 | - | 1.21475 | 0.542919 | LincRNA | . | . | . | . | . |
| XLOC_055332 | chr3:129103429-129239295 | - | 1.67916 | 1.16132 | Pseudogene | ENSG00000244932.2 | RP11-529F4.1 | . | . | . |
| XLOC_055363 | chr3:131081207-131099574 | - | 1.2928 | 1.3529 | LincRNA | ENSG00000250608.1 | RP11-933H2.4 | . | . | . |
| XLOC_055391 | chr3:134101261-134112946 | - | 0.0874032 | 1.66552 | Pseudogene | ENSG00000248377.1 | HMGN1P9 | . | . | . |
| XLOC_055417 | chr3:137745520-137816689 | - | 1.28629 | 0.647818 | LincRNA | . | . | . | . | . |
| XLOC_055517 | chr3:145701437-145710978 | - | 1.12276 | 1.0952 | LincRNA | . | . | . | . | . |
| XLOC_055679 | chr3:156465853-156469447 | - | 1.23336 | 0.611389 | LincRNA | ENSG00000240875.1 | LINC00886 | . | . | . |
| XLOC_055900 | chr3:178626152-178722317 | - | 3.60855 | 3.94667 | LincRNA | . | . | . | . | . |
| XLOC_055906 | chr3:178626152-178722317 | - | 1.64814 | 1.83775 | LincRNA | . | . | . | . | . |
| XLOC_055944 | chr3:180284598-180302365 | - | 1.89415 | 0.742316 | Pseudogene | ENSG00000241946.1 | RP11-496B10.1 | . | . | . |
| XLOC_056097 | chr3:194535851-194539577 | - | 0.010732 | 1.10831 | LincRNA | . | . | . | . | . |
| XLOC_056116 | chr3:195685913-195717169 | - | 2.72977 | 2.85763 | Pseudogene | ENSG00000185485.10 | SDHAP1 | . | . | . |
| XLOC_056156 | chr3:197330569-197354758 | - | 4.60969 | 5.07124 | Pseudogene | ENSG00000214135.4 | AC024560.3 | . | . | . |
| XLOC_056186 | chr4:124399-156992 | + | 3.31994 | 3.18908 | LincRNA | ENSG00000250312.2 | ZNF718 | . | . | . |
| XLOC_056223 | chr4:206392-248841 | + | 4.57045 | 5.02096 | Pseudogene | ENSG00000198155.5 | ZNF876P | . | . | . |
| XLOC_056288 | chr4:1243646-1247825 | + | 1.18221 | 0.84234 | LincRNA | ENSG00000196810.4 | CTBP1-AS2 | . | . | . |
| XLOC_056315 | chr4:2936616-2965147 | + | 2.30789 | 2.30265 | Antisense | ENSG00000249673.2 | NOP14-AS1 | . | . | . |
| XLOC_056339 | chr4:4543837-4587913 | + | 1.38266 | 0.42836 | Antisense | ENSG00000247708.3 | STX18-AS1 | XLOC_003863 | linc-STX18-1 | . |
| XLOC_056386 | chr4:6675796-6678011 | + | 3.75885 | 2.97639 | LincRNA | ENSG00000251580.1 | RP11-539L10.3 | . | . | . |
| XLOC_056563 | chr4:11370163-11374658 | + | 4.14532 | 3.33795 | Pseudogene | ENSG00000250896.1 | RNPS1P1 | . | . | . |
| XLOC_056609 | chr4:16248711-16262617 | + | 1.12806 | 0.387674 | LincRNA | ENSG00000263327.2 | TAPT1-AS1 | . | . | . |
| XLOC_056625 | chr4:16289222-16324811 | + | 1.15553 | 0.702634 | LincRNA | ENSG00000263327.2 | TAPT1-AS1 | XLOC_003469 | linc-CLRN2-3 | . |
| XLOC_056958 | chr4:52808472-52870014 | + | 0.49183 | 4.48214 | Pseudogene | ENSG00000243929.1 | RP11-61F5.1 | . | . | . |
| XLOC_056977 | chr4:53525612-53532354 | + | 2.44111 | 1.8855 | LincRNA | ENSG00000248866.1 | USP46-AS1 | . | . | . |
| XLOC_056980 | chr4:53578622-53582444 | + | 1.85461 | 1.54265 | LincRNA | ENSG00000226950.2 | DANCR | . | . | . |
| XLOC_057065 | chr4:68566999-68656219 | + | 5.78271 | 4.29641 | Pseudogene | ENSG00000227725.2 | GCOM2 | . | . | . |
| XLOC_057098 | chr4:68660437-68670814 | + | 1.84851 | 0.829915 | Pseudogene | ENSG00000227725.2 | GCOM2 | . | . | . |
| XLOC_057143 | chr4:68904285-68905489 | + | 11.7187 | 10.1781 | Pseudogene | ENSG00000227725.2 | GCOM2 | . | . | . |
| XLOC_057168 | chr4:74135360-74170012 | + | 2.03781 | 1.10003 | LincRNA | ENSG00000250220.1 | RP11-692D12.1 | . | . | . |
| XLOC_057398 | chr4:85741233-85929985 | + | 1.58795 | 1.36735 | LincRNA | . | . | . | . | . |
| XLOC_057548 | chr4:100054266-100077627 | + | 1.57641 | 1.22733 | Antisense | ENSG00000246090.2 | RP11-696N14.1 | . | . | . |
| XLOC_057740 | chr4:119199873-119204897 | + | 2.3899 | 1.87338 | Antisense | ENSG00000269893.2 | SNHG8 | . | . | . |
| XLOC_057745 | chr4:119401464-119414358 | + | 1.42896 | 2.6817 | Pseudogene | ENSG00000154608.9 | CEP170P1 | . | . | . |
| XLOC_057765 | chr4:119757865-119772778 | + | 1.79975 | 2.04261 | LincRNA | . | . | XLOC_003665 | linc-SYNPO2-2 | . |
| XLOC_057787 | chr4:120375975-120550085 | + | 1.7933 | 1.2729 | Pseudogene | ENSG00000245958.2 | RP11-33B1.1 | . | . | . |
| XLOC_058057 | chr4:144480637-144531015 | + | 2.65057 | 0.318109 | Pseudogene | ENSG00000236296.3 | GUSBP5 | . | . | . |
| XLOC_058141 | chr4:144729487-144741804 | + | 0.627154 | 1.4476 | Pseudogene | ENSG00000250345.2 | RP11-780M14.1 | XLOC_004107 | linc-FREM3 | . |
| XLOC_058158 | chr4:144779635-144888296 | + | 1.51349 | 3.02038 | Pseudogene | ENSG00000250345.2 | RP11-780M14.1 | . | . | . |
| XLOC_058267 | chr4:154869980-155122950 | + | 6.48944 | 14.0821 | LincRNA | . | . | . | . | . |
| XLOC_058368 | chr4:155127365-155224132 | + | 0.565782 | 1.01296 | Antisense | . | . | . | . | . |
| XLOC_058567 | chr4:174430853-174432142 | + | 2.62179 | 2.5457 | LincRNA | . | . | XLOC_003783 | linc-KIAA1712-4 | . |
| XLOC_058570 | chr4:174447778-174512356 | + | 0.359543 | 1.84638 | Antisense | ENSG00000237125.4 | HAND2-AS1 | . | . | . |
| XLOC_058736 | chr4:189659524-189663157 | + | 3.22989 | 4.82623 | Pseudogene | ENSG00000180015.11 | RP11-756P10.3 | . | . | . |
| XLOC_058775 | chr4:770695-775638 | - | 3.1031 | 3.81293 | LincRNA | ENSG00000249592.1 | RP11-440L14.1 | . | . | . |
| XLOC_058819 | chr4:3952902-3957196 | - | 2.8034 | 0 | Pseudogene | ENSG00000251669.1 | FAM86EP | . | . | . |
| XLOC_058873 | chr4:9132526-9154033 | - | 1.20731 | 0.556386 | Pseudogene | ENSG00000249767.1 | ENPP7P10 | . | . | . |
| XLOC_059049 | chr4:14986929-14995262 | - | 0.92339 | 1.28821 | LincRNA | ENSG00000247624.2 | CPEB2-AS1 | . | . | . |
| XLOC_059330 | chr4:24455480-24474340 | - | 1.66434 | 1.7205 | LincRNA | . | . | XLOC_003908 | linc-PPARGC1A | . |
| XLOC_059345 | chr4:24763373-24765607 | - | 1.17903 | 0.0987755 | LincRNA | . | . | . | . | . |
| XLOC_059673 | chr4:74568381-74588776 | - | 0.0117882 | 4.84785 | LincRNA | ENSG00000228277.1 | AC112518.3 | XLOC_003573 | linc-IL8-1 | . |
| XLOC_059750 | chr4:77830049-77870708 | - | 1.04297 | 1.14586 | LincRNA | . | . | XLOC_003991 | linc-ANKRD56-1 | . |
| XLOC_060027 | chr4:109538177-109541532 | - | 1.11762 | 0.499027 | LincRNA | ENSG00000234492.4 | RPL34-AS1 | . | . | . |
| XLOC_060080 | chr4:114351762-114357556 | - | 5.2207 | 5.29754 | LincRNA | . | . | . | . | eRNA |
| XLOC_060117 | chr4:115256035-115286136 | - | 1.27477 | 2.66234 | LincRNA | . | . | XLOC_004049 | linc-ARSJ | . |
| XLOC_060150 | chr4:120355777-120375770 | - | 2.1838 | 1.50613 | Pseudogene | ENSG00000248280.1 | RP11-33B1.2 | . | . | . |
| XLOC_060211 | chr4:123651643-123653594 | - | 1.47986 | 1.0931 | Pseudogene | ENSG00000224786.1 | CETN4P | . | . | . |
| XLOC_060647 | chr4:142244712-142253770 | - | 2.79242 | 2.22537 | LincRNA | ENSG00000248810.1 | RP11-362F19.1 | . | . | . |
| XLOC_060725 | chr4:145564119-145567158 | - | 0.886616 | 1.01046 | LincRNA | ENSG00000248890.1 | HHIP-AS1 | . | . | . |
| XLOC_060842 | chr4:158573954-158598790 | - | 0.0131208 | 2.14144 | LincRNA | ENSG00000249275.1 | RP11-364P22.2 | XLOC_003759 | linc-TMEM144-1 | . |
| XLOC_060976 | chr4:174087806-174249092 | - | 1.65836 | 1.14299 | LincRNA | ENSG00000245213.2 | RP11-10K16.1 | . | . | . |
| XLOC_060983 | chr4:174284931-174291879 | - | 2.13556 | 1.18489 | Antisense | ENSG00000272870.1 | RP11-798M19.6 | XLOC_003780 | linc-SAP30 | . |
| XLOC_061064 | chr4:184413344-184425626 | - | 2.26695 | 0.928806 | LincRNA | ENSG00000232648.3 | RP11-367N14.2 | . | . | . |
| XLOC_061178 | chr4_gl000194_random:53607-115069 | - | 2.86415 | 3.64098 | LincRNA | . | . | . | . | . |
| XLOC_061280 | chr5:8457800-8460190 | + | 44.4503 | 57.6557 | LincRNA | ENSG00000249159.2 | RP11-480D4.2 | XLOC_004284 | linc-CCT5-8 | . |
| XLOC_061283 | chr5:9546406-9553232 | + | 1.56616 | 0.644405 | LincRNA | ENSG00000250786.1 | SNHG18 | . | . | . |
| XLOC_061422 | chr5:17604295-17606015 | + | 0.22736 | 3.83248 | Pseudogene | ENSG00000249357.2 | RP11-432M8.8 | . | . | . |
| XLOC_061423 | chr5:17743886-17791828 | + | 1.9545 | 1.24204 | LincRNA | ENSG00000249937.1 | RP11-454P21.1 | . | . | . |
| XLOC_061444 | chr5:21459593-21475202 | + | 7.66555 | 5.58109 | Pseudogene | ENSG00000183666.12 | GUSBP1 | . | . | . |
| XLOC_061490 | chr5:27475700-27486171 | + | 2.81016 | 0.843 | LincRNA | ENSG00000250337.1 | LINC01021 | XLOC_004323 | linc-CDH6-5 | . |
| XLOC_061521 | chr5:32828826-32890967 | + | 2.45808 | 0.699052 | Pseudogene | ENSG00000251062.1 | CTD-2218G20.1 | . | . | . |
| XLOC_061582 | chr5:35366494-35422974 | + | 0.172064 | 3.58667 | LincRNA | . | . | . | . | . |
| XLOC_061661 | chr5:41281482-41350879 | + | 0.138734 | 2.55131 | Antisense | . | . | XLOC_004799 | linc-C6 | . |
| XLOC_061706 | chr5:43013739-43067530 | + | 1.34717 | 1.02076 | LincRNA | ENSG00000272144.1 | CTD-2035E11.5 | XLOC_004364 | linc-ZNF131-3 | . |
| XLOC_061801 | chr5:52405704-52411150 | + | 2.40261 | 1.80243 | Antisense | ENSG00000247796.2 | CTD-2366F13.1 | XLOC_004382 | linc-FST | . |
| XLOC_061904 | chr5:59827821-59843475 | + | 0.288008 | 1.22449 | LincRNA | ENSG00000152931.7 | PART1 | XLOC_004399 | linc-NDUFAF2-3 | . |
| XLOC_061928 | chr5:60476212-60477989 | + | 1.39722 | 2.3811 | LincRNA | ENSG00000251279.1 | CTC-436P18.1 | . | . | . |
| XLOC_061956 | chr5:64327144-64365959 | + | 3.81597 | 16.4397 | LincRNA | . | . | XLOC_004407 | linc-PPWD1 | eRNA |
| XLOC_062169 | chr5:76414261-76443815 | + | 1.33531 | 0.459709 | LincRNA | ENSG00000250802.2 | ZBED3-AS1 | . | . | . |
| XLOC_062280 | chr5:83877293-83899494 | + | 0.647917 | 1.36878 | LincRNA | . | . | . | . | . |
| XLOC_062322 | chr5:87564789-87594967 | + | 2.76795 | 2.01271 | LincRNA | ENSG00000271904.1 | CTC-498M16.4 | XLOC_004456 | linc-POLR3G-11 | . |
| XLOC_062479 | chr5:95308147-95715995 | + | 1.59818 | 0.990353 | Pseudogene | ENSG00000213716.3 | FABP5P5 | XLOC_004933 | linc-ELL2 | eRNA |
| XLOC_063037 | chr5:111496541-111755225 | + | 2.07482 | 1.40692 | Antisense | ENSG00000251076.1 | RP11-526F3.1 | . | . | . |
| XLOC_063090 | chr5:115783139-115806943 | + | 1.77133 | 0.892345 | Antisense | ENSG00000248445.1 | CTB-118N6.3 | . | . | . |
| XLOC_063522 | chr5:141704726-141961462 | + | 2.82808 | 3.2891 | LincRNA | ENSG00000231185.2 | AC005592.2 | XLOC_004587 | linc-ARHGAP26-4 | . |
| XLOC_063809 | chr5:158527577-158535901 | + | 3.42375 | 1.80523 | LincRNA | ENSG00000245812.2 | RP11-175K6.1 | . | . | . |
| XLOC_063823 | chr5:159895208-159922844 | + | 0.0597088 | 9.34461 | LincRNA | ENSG00000253522.2 | MIR146A | . | . | . |
| XLOC_063920 | chr5:171006805-171007589 | + | 0.580126 | 1.08651 | LincRNA | . | . | . | . | . |
| XLOC_063987 | chr5:177378726-177387043 | + | 0.0328867 | 1.04646 | Pseudogene | ENSG00000214351.5 | OR1X5P | . | . | . |
| XLOC_064050 | chr5:180619093-180621685 | + | 2.48314 | 7.55652 | Antisense | ENSG00000250222.1 | CTC-338M12.5 | . | . | . |
| XLOC_064062 | chr5:180688236-180691786 | + | 0.974301 | 1.38522 | LincRNA | ENSG00000248275.1 | TRIM52-AS1 | . | . | . |
| XLOC_064093 | chr5:1572065-1586302 | - | 3.59818 | 2.85402 | Pseudogene | ENSG00000185986.10 | SDHAP3 | . | . | . |
| XLOC_064099 | chr5:1597513-1629733 | - | 3.3765 | 2.90344 | Pseudogene | ENSG00000188002.6 | RP11-43F13.1 | . | . | . |
| XLOC_064213 | chr5:16437064-16448804 | - | 0.586006 | 1.49028 | LincRNA | ENSG00000249737.1 | RP1-167G20.2 | XLOC_004303 | linc-BASP1-3 | . |
| XLOC_064397 | chr5:38466286-38468506 | - | 2.06869 | 1.76617 | LincRNA | ENSG00000251257.1 | CTD-2263F21.1 | . | . | . |
| XLOC_064401 | chr5:38686194-38796576 | - | 1.17836 | 2.50054 | LincRNA | ENSG00000249911.1 | RP11-122C5.1 | XLOC_004346 | linc-OSMR | . |
| XLOC_064439 | chr5:38843603-38845909 | - | 2.50623 | 3.20887 | LincRNA | ENSG00000250629.1 | RP11-122C5.3 | . | . | . |
| XLOC_064652 | chr5:57545844-57555400 | - | 0.482317 | 2.98545 | LincRNA | . | . | . | . | . |
| XLOC_064750 | chr5:64782094-64782753 | - | 0.318063 | 1.22469 | LincRNA | ENSG00000250081.1 | CTD-2116N20.1 | XLOC_004847 | linc-ADAMTS6 | . |
| XLOC_064850 | chr5:72542093-72570795 | - | 1.82686 | 1.9884 | LincRNA | ENSG00000249743.1 | RP11-60A8.1 | XLOC_004423 | linc-BTF3-5 | . |
| XLOC_064941 | chr5:79780526-79842124 | - | 1.02089 | 0.935896 | LincRNA | ENSG00000249042.1 | CTD-2015H6.3 | . | . | . |
| XLOC_064957 | chr5:80533385-80597343 | - | 2.6244 | 1.71528 | Antisense | ENSG00000247572.3 | CKMT2-AS1 | . | . | . |
| XLOC_065146 | chr5:90596973-90610312 | - | 1.254 | 11.9774 | LincRNA | ENSG00000248323.1 | LUCAT1 | XLOC_004468 | linc-NR2F1-7 | . |
| XLOC_065148 | chr5:92746727-92916889 | - | 2.75142 | 3.92486 | LincRNA | ENSG00000237187.4 | NR2F1-AS1 | XLOC_004475 | linc-NR2F1-1 | . |
| XLOC_065331 | chr5:99854042-99870684 | - | 1.31521 | 0.0194418 | LincRNA | ENSG00000247877.2 | CTD-2001C12.1 | . | . | . |
| XLOC_065498 | chr5:108655310-108745772 | - | 102.92 | 135.339 | LincRNA | ENSG00000249476.1 | CTD-2587M2.1 | . | . | . |
| XLOC_065778 | chr5:127350422-127418798 | - | 5.25319 | 2.50472 | LincRNA | ENSG00000245937.3 | CTC-228N24.3 | . | . | . |
| XLOC_065919 | chr5:135465416-135518912 | - | 1.00626 | 0.205029 | Antisense | ENSG00000164621.5 | SMAD5-AS1 | . | . | . |
| XLOC_066017 | chr5:139482534-139488340 | - | 2.22111 | 2.71245 | Antisense | ENSG00000245146.2 | LINC01024 | . | . | . |
| XLOC_066129 | chr5:144733545-144769347 | - | 0.473299 | 2.16266 | LincRNA | ENSG00000250842.1 | CTC-806A22.1 | XLOC_005039 | linc-YIPF5-2 | . |
| XLOC_066138 | chr5:145039239-145214926 | - | 0.796887 | 1.46613 | LincRNA | ENSG00000248125.1 | CTB-73N10.1 | XLOC_005041 | linc-YIPF5-3 | . |
| XLOC_066163 | chr5:146556638-146563169 | - | 1.18843 | 0.294413 | LincRNA | ENSG00000250343.1 | CTC-255N20.1 | XLOC_005046 | linc-PPP2R2B-2 | . |
| XLOC_066283 | chr5:150294822-150333973 | - | 9.21808 | 9.45466 | Pseudogene | ENSG00000197083.7 | ZNF300P1 | . | . | . |
| XLOC_066290 | chr5:150348010-150377882 | - | 0.0239596 | 2.27183 | LincRNA | . | . | . | . | . |
| XLOC_066493 | chr5:177041064-177099238 | - | 1.58259 | 1.03053 | LincRNA | ENSG00000247679.2 | RP11-1277A3.1 | . | . | . |
| XLOC_066615 | chr6:2246002-2397846 | + | 4.68423 | 3.32386 | LincRNA | ENSG00000250903.4 | GMDS-AS1 | XLOC_005127 | linc-WRNIP1-2 | . |
| XLOC_066674 | chr6:2397921-2398996 | + | 0.632073 | 1.21283 | LincRNA | ENSG00000250903.4 | GMDS-AS1 | XLOC_005127 | linc-WRNIP1-2 | . |
| XLOC_066675 | chr6:2399113-2434707 | + | 0.958927 | 7.87007 | LincRNA | ENSG00000250903.4 | GMDS-AS1 | XLOC_005127 | linc-WRNIP1-2 | . |
| XLOC_066734 | chr6:3752338-3754624 | + | 1.15932 | 0.971587 | Pseudogene | ENSG00000219992.2 | RP11-420L9.2 | . | . | . |
| XLOC_066780 | chr6:7673545-7676259 | + | 0.166788 | 1.56789 | LincRNA | . | . | . | . | . |
| XLOC_066793 | chr6:8435856-8559663 | + | 2.57554 | 1.4391 | LincRNA | . | . | . | . | . |
| XLOC_066990 | chr6:14704157-14709984 | + | 0.148749 | 1.6542 | LincRNA | . | . | . | . | . |
| XLOC_067063 | chr6:21873759-22196308 | + | 1.64118 | 1.48668 | Pseudogene | ENSG00000219404.2 | RP11-524C21.1 | XLOC_005639 | linc-MBOAT1-2 | . |
| XLOC_067205 | chr6:26426668-26466203 | + | 1.81908 | 4.86318 | Pseudogene | ENSG00000124549.10 | BTN2A3P | . | . | . |
| XLOC_067241 | chr6:26988142-26990720 | + | 0.573479 | 1.42234 | LincRNA | ENSG00000224843.2 | LINC00240 | . | . | . |
| XLOC_067314 | chr6:28234763-28246288 | + | 7.31378 | 4.84344 | Pseudogene | ENSG00000197062.7 | RP5-874C20.3 | . | . | . |
| XLOC_067355 | chr6:34664746-34665240 | + | 7.34444 | 3.85429 | LincRNA | ENSG00000272288.1 | RP11-140K17.3 | . | . | . |
| XLOC_067394 | chr6:35936578-35968897 | + | 0.218798 | 2.65398 | Pseudogene | ENSG00000271304.1 | DPRXP2 | . | . | . |
| XLOC_067489 | chr6:41040714-41095311 | + | 15.268 | 19.7516 | Pseudogene | ENSG00000161912.13 | ADCY10P1 | . | . | . |
| XLOC_067603 | chr6:52442099-52447757 | + | 3.91119 | 3.50065 | LincRNA | ENSG00000225791.2 | TRAM2-AS1 | . | . | . |
| XLOC_067606 | chr6:52529231-52534103 | + | 10.5178 | 3.78112 | Pseudogene | ENSG00000216775.2 | RP1-152L7.5 | XLOC_005323 | linc-TMEM14A-1 | . |
| XLOC_067619 | chr6:53493079-53496446 | + | 3.62346 | 3.00556 | LincRNA | ENSG00000235899.1 | RP11-345L23.1 | XLOC_005327 | linc-LRRC1-1 | . |
| XLOC_067654 | chr6:57139795-57141891 | + | 0.890397 | 1.65879 | LincRNA | . | . | . | . | . |
| XLOC_067698 | chr6:72313651-72490122 | + | 1.38443 | 1.98925 | LincRNA | . | . | . | . | . |
| XLOC_067711 | chr6:73951075-74019883 | + | 1.11223 | 0.777968 | Pseudogene | ENSG00000239626.1 | RPSAP41 | . | . | . |
| XLOC_067770 | chr6:75994707-76000820 | + | 2.15587 | 1.73405 | LincRNA | ENSG00000225793.2 | RP1-234P15.4 | . | . | . |
| XLOC_067818 | chr6:80194708-80413410 | + | 6.52449 | 2.23866 | LincRNA | . | . | XLOC_005361 | linc-SH3BGRL2-1 | . |
| XLOC_067913 | chr6:81289828-81357379 | + | 2.70623 | 1.85341 | LincRNA | . | . | . | . | . |
| XLOC_068220 | chr6:90539642-90581376 | + | 10.246 | 7.46548 | LincRNA | . | . | . | . | . |
| XLOC_068266 | chr6:99968591-99983754 | + | 2.02601 | 1.2494 | LincRNA | ENSG00000228439.3 | TSTD3 | . | . | . |
| XLOC_068297 | chr6:105585177-105595380 | + | 1.32555 | 1.05075 | LincRNA | ENSG00000203808.6 | BVES-AS1 | . | . | . |
| XLOC_068414 | chr6:111596861-111599533 | + | 1.15158 | 0.967829 | LincRNA | ENSG00000230177.1 | RP5-1112D6.4 | XLOC_005426 | linc-WISP3-3 | . |
| XLOC_068456 | chr6:112337892-112353718 | + | 0 | 2.38924 | LincRNA | . | . | . | . | . |
| XLOC_068537 | chr6:119497178-119748361 | + | 2.83542 | 4.08007 | LincRNA | . | . | XLOC_005448 | linc-GJA1-3 | . |
| XLOC_068746 | chr6:126307584-126521571 | + | 30.3444 | 16.7933 | LincRNA | . | . | XLOC_005459 | linc-CENPW-1 | . |
| XLOC_069015 | chr6:132455027-132490685 | + | 2.64934 | 3.19306 | LincRNA | ENSG00000228495.1 | LINC01013 | XLOC_005465 | linc-TAAR9-1 | . |
| XLOC_069077 | chr6:138014249-138023121 | + | 0.154571 | 1.85035 | LincRNA | . | . | . | . | . |
| XLOC_069177 | chr6:146183785-146199372 | + | 1.17183 | 0.733724 | Antisense | ENSG00000235652.3 | RP11-545I5.3 | . | . | . |
| XLOC_069424 | chr6:157097173-157098295 | + | 3.12416 | 0.557848 | LincRNA | ENSG00000271551.1 | RP11-230C9.2 | . | . | . |
| XLOC_069431 | chr6:158187092-158217344 | + | 0.942315 | 2.48429 | LincRNA | . | . | XLOC_005516 | linc-SNX9 | . |
| XLOC_069862 | chr6:14875558-14877423 | - | 0.0829864 | 1.58611 | LincRNA | . | . | . | . | . |
| XLOC_069886 | chr6:19806422-19811851 | - | 1.44145 | 0.561298 | LincRNA | ENSG00000226786.2 | RP1-167F1.2 | XLOC_005193 | linc-ID4-1 | . |
| XLOC_069920 | chr6:25080488-25138907 | - | 11.1212 | 7.94537 | Pseudogene | ENSG00000168405.10 | CMAHP | . | . | . |
| XLOC_070054 | chr6:27325608-27343032 | - | 2.86849 | 2.00299 | Pseudogene | ENSG00000204789.3 | ZNF204P | . | . | . |
| XLOC_070192 | chr6:40854994-40857146 | - | 0.279115 | 1.64382 | LincRNA | . | . | XLOC_005715 | linc-LRFN2-1 | . |
| XLOC_070323 | chr6:48036929-48079248 | - | 1.56307 | 0.645016 | LincRNA | . | . | . | . | . |
| XLOC_070349 | chr6:51274648-51275317 | - | 56.0624 | 314.391 | Pseudogene | ENSG00000232702.2 | RP3-437C15.1 | . | . | . |
| XLOC_070471 | chr6:58271991-58287707 | - | 8.15523 | 5.65271 | LincRNA | . | . | . | . | . |
| XLOC_070579 | chr6:75346057-75443928 | - | 1.00059 | 0.0637928 | LincRNA | ENSG00000224583.1 | RP11-554D15.4 | XLOC_005348 | linc-SENP6-6 | . |
| XLOC_070748 | chr6:77856652-78078981 | - | 4.45091 | 2.64941 | LincRNA | . | . | XLOC_005768 | linc-IMPG1-1 | . |
| XLOC_071050 | chr6:82554339-82605761 | - | 1.17188 | 0.741563 | LincRNA | ENSG00000226453.1 | RP11-379B8.1 | XLOC_005366 | linc-TPBG-2 | . |
| XLOC_071182 | chr6:86374367-86388496 | - | 13.2343 | 11.156 | LincRNA | ENSG00000203875.6 | SNHG5 | . | . | . |
| XLOC_071237 | chr6:96006587-96025239 | - | 3.42246 | 1.35332 | LincRNA | ENSG00000261366.1 | MANEA-AS1 | . | . | . |
| XLOC_071433 | chr6:112605582-112610269 | - | 0.637486 | 1.67847 | Pseudogene | ENSG00000216663.3 | RP11-506B6.5 | . | . | . |
| XLOC_071446 | chr6:113938137-113946289 | - | 0.01407 | 1.14421 | LincRNA | ENSG00000230943.1 | RP11-367G18.1 | XLOC_005810 | linc-LAMA4-2 | . |
| XLOC_071449 | chr6:113946487-113975857 | - | 0.0468708 | 2.17102 | LincRNA | ENSG00000230943.1 | RP11-367G18.1 | XLOC_005810 | linc-LAMA4-2 | eRNA |
| XLOC_071797 | chr6:127175941-127215669 | - | 0.216655 | 2.24546 | LincRNA | . | . | XLOC_005460 | linc-RSPO3 | . |
| XLOC_072067 | chr6:138175998-138186493 | - | 1.10512 | 2.8659 | LincRNA | ENSG00000237499.2 | RP11-356I2.4 | XLOC_005479 | linc-TNFAIP3-1 | . |
| XLOC_072138 | chr6:141168506-141219545 | - | 5.43718 | 3.20995 | LincRNA | ENSG00000234147.1 | RP3-460G2.2 | . | . | . |
| XLOC_072160 | chr6:143360554-143363440 | - | 1.99481 | 2.33521 | LincRNA | ENSG00000227192.1 | RP1-45I4.3 | . | . | . |
| XLOC_072228 | chr6:147167519-147293203 | - | 1.38347 | 1.4306 | LincRNA | ENSG00000272397.1 | RP11-497D6.5 | XLOC_005495 | linc-STXBP5-2 | eRNA |
| XLOC_072255 | chr6:147167519-147293203 | - | 1.40872 | 1.82876 | LincRNA | ENSG00000272397.1 | RP11-497D6.5 | XLOC_005495 | linc-STXBP5-2 | eRNA |
| XLOC_072411 | chr6:159586993-159588613 | - | 0.305333 | 1.33782 | LincRNA | ENSG00000233682.2 | RP11-13P5.2 | XLOC_005522 | linc-FNDC1-1 | . |
| XLOC_072456 | chr6:160007870-160013184 | - | 0.0848209 | 3.7715 | LincRNA | ENSG00000237927.1 | RP3-393E18.2 | XLOC_005900 | linc-TAGAP-1 | . |
| XLOC_072471 | chr6:160898178-160977144 | - | 1.96176 | 1.18576 | Pseudogene | ENSG00000213071.6 | LPAL2 | . | . | . |
| XLOC_072568 | chr6_cox_hap2:2752099-2753128 | - | 35.006 | 36.9063 | LincRNA | . | . | . | . | . |
| XLOC_072569 | chr6_cox_hap2:2835106-2837726 | - | 31.5388 | 42.7233 | LincRNA | . | . | . | . | . |
| XLOC_072577 | chr6_dbb_hap3:2534447-2535480 | - | 1.7735 | 2.47793 | LincRNA | . | . | . | . | . |
| XLOC_072582 | chr6_mann_hap4:2669981-2671584 | - | 1.7995 | 2.38362 | LincRNA | . | . | . | . | . |
| XLOC_072586 | chr6_mcf_hap5:2617060-2704524 | - | 37.0496 | 36.638 | LincRNA | . | . | . | . | . |
| XLOC_072590 | chr6_qbl_hap6:1208116-1210166 | + | 0.527708 | 1.09796 | LincRNA | . | . | . | . | . |
| XLOC_072596 | chr6_qbl_hap6:2661929-2676693 | + | 2.33664 | 2.14781 | LincRNA | . | . | . | . | . |
| XLOC_072604 | chr6_qbl_hap6:2617460-2618499 | - | 2.69937 | 5.80173 | LincRNA | . | . | . | . | . |
| XLOC_072609 | chr6_ssto_hap7:1032091-1033953 | - | 1.7175 | 1.51649 | LincRNA | . | . | . | . | . |
| XLOC_072624 | chr7:591021-770227 | + | 1.7003 | 2.60213 | Antisense | ENSG00000237181.1 | AC147651.4 | . | . | . |
| XLOC_072629 | chr7:1200019-1205669 | + | 2.07328 | 1.49328 | LincRNA | ENSG00000229043.2 | AC091729.9 | . | . | . |
| XLOC_072634 | chr7:1606967-1615669 | + | 1.87472 | 0.737665 | Antisense | ENSG00000230487.3 | PSMG3-AS1 | . | . | . |
| XLOC_072651 | chr7:2482181-2483511 | + | 1.99439 | 1.11676 | LincRNA | ENSG00000175873.3 | AC004840.9 | XLOC_005968 | linc-LFNG | . |
| XLOC_072663 | chr7:2930288-2932310 | + | 0.0855585 | 1.29678 | LincRNA | . | . | . | . | . |
| XLOC_072718 | chr7:6770030-6793571 | + | 2.52139 | 3.23337 | Pseudogene | ENSG00000187953.6 | PMS2CL | . | . | . |
| XLOC_072861 | chr7:17414371-17507370 | + | 1.60012 | 2.26291 | LincRNA | ENSG00000236318.1 | AC019117.1 | . | . | . |
| XLOC_072981 | chr7:19592362-19628875 | + | 1.04917 | 0.14373 | LincRNA | ENSG00000223838.1 | AC007091.1 | . | . | . |
| XLOC_073038 | chr7:22893876-22901318 | + | 1.84432 | 2.76303 | LincRNA | ENSG00000228649.4 | AC005682.5 | . | . | eRNA |
| XLOC_073074 | chr7:25878714-25888251 | + | 0.775728 | 1.01164 | LincRNA | . | . | XLOC_006016 | linc-NFE2L3-1 | . |
| XLOC_073085 | chr7:27135742-27141203 | + | 0.862918 | 1.24485 | Antisense | ENSG00000233429.5 | HOTAIRM1 | . | . | . |
| XLOC_073140 | chr7:30174486-30226478 | + | 6.72939 | 8.78362 | LincRNA | ENSG00000227017.1 | AC007036.6 | XLOC_006034 | linc-ZNRF2 | . |
| XLOC_073154 | chr7:30256337-30273686 | + | 1.03508 | 2.06383 | LincRNA | . | . | . | . | . |
| XLOC_073354 | chr7:39773059-39818967 | + | 1.22728 | 0.576825 | LincRNA | ENSG00000188185.7 | LINC00265 | XLOC_006416 | linc-VPS41-3 | . |
| XLOC_073404 | chr7:41743563-41750728 | + | 0.333471 | 3.12811 | LincRNA | ENSG00000224116.2 | INHBA-AS1 | . | . | . |
| XLOC_073502 | chr7:45964203-45968791 | + | 0.628757 | 1.18671 | LincRNA | . | . | . | . | . |
| XLOC_073505 | chr7:46072766-46076950 | + | 0 | 1.02754 | LincRNA | . | . | . | . | . |
| XLOC_073614 | chr7:64408060-64409790 | + | 1.09224 | 1.51977 | LincRNA | . | . | . | . | . |
| XLOC_073647 | chr7:65217889-65229576 | + | 1.82339 | 1.92863 | Pseudogene | ENSG00000228409.1 | CCT6P1 | . | . | . |
| XLOC_073687 | chr7:66767591-66796469 | + | 5.78807 | 4.9121 | Pseudogene | ENSG00000067601.6 | PMS2P4 | XLOC_006485 | linc-SBDS-4 | . |
| XLOC_073716 | chr7:72300007-72307265 | + | 64.9038 | 67.8424 | Pseudogene | ENSG00000225648.1 | SBDSP1 | . | . | . |
| XLOC_073835 | chr7:78954080-79092253 | + | 13.9403 | 28.3723 | Antisense | ENSG00000234456.3 | MAGI2-AS3 | XLOC_006507 | linc-MAGI2-3 | . |
| XLOC_073967 | chr7:84335287-84388592 | + | 0.699894 | 3.39278 | LincRNA | . | . | . | . | . |
| XLOC_074117 | chr7:93690218-93697178 | + | 1.05607 | 2.15037 | LincRNA | ENSG00000236453.1 | AC003092.1 | . | . | . |
| XLOC_074222 | chr7:98895298-98895600 | + | 0 | 5.10982 | Pseudogene | ENSG00000002079.8 | MYH16 | . | . | . |
| XLOC_074266 | chr7:99517273-99520812 | + | 1.44008 | 0.762312 | LincRNA | . | . | XLOC_006187 | linc-ZKSCAN1-2 | . |
| XLOC_074299 | chr7:100951562-100954190 | + | 1.1019 | 0.314558 | LincRNA | ENSG00000232445.1 | RP11-132A1.4 | . | . | . |
| XLOC_074491 | chr7:114762272-114765872 | + | 0.861166 | 1.17473 | LincRNA | ENSG00000225535.2 | AC068610.3 | . | . | . |
| XLOC_074723 | chr7:128171544-128269470 | + | 11.2348 | 11.2506 | Pseudogene | ENSG00000242588.2 | RP11-274B21.1 | . | . | . |
| XLOC_074750 | chr7:128271599-128285417 | + | 2.67637 | 2.30811 | LincRNA | . | . | XLOC_006242 | linc-FAM71F2-1 | . |
| XLOC_074820 | chr7:133781951-133787047 | + | 0.508618 | 1.13737 | LincRNA | . | . | XLOC_006259 | linc-LRGUK-1 | . |
| XLOC_074828 | chr7:133803999-133948697 | + | 15.223 | 19.0816 | LincRNA | . | . | . | . | . |
| XLOC_074956 | chr7:139953048-139957857 | + | 0.420158 | 6.3814 | LincRNA | . | . | . | . | . |
| XLOC_075030 | chr7:143521109-143599248 | + | 1.37535 | 0.594743 | Pseudogene | ENSG00000232145.1 | RP11-307I2.1 | . | . | . |
| XLOC_075121 | chr7:155005908-155019377 | + | 0.363989 | 1.20011 | LincRNA | . | . | XLOC_006303 | linc-INSIG1-2 | . |
| XLOC_075204 | chr7:4684757-4701106 | - | 0.536025 | 3.80146 | Antisense | . | . | . | . | . |
| XLOC_075258 | chr7:7591079-7592579 | - | 1.70554 | 0.144651 | LincRNA | ENSG00000272894.1 | RP5-1159O4.1 | XLOC_006350 | linc-COL28A1 | . |
| XLOC_075354 | chr7:17522222-17523142 | - | 1.96864 | 1.09746 | LincRNA | ENSG00000236318.1 | AC019117.1 | . | . | . |
| XLOC_075404 | chr7:19178631-19187338 | - | 2.04477 | 1.76045 | LincRNA | . | . | . | . | . |
| XLOC_075413 | chr7:20085136-20118050 | - | 6.59717 | 1.10649 | LincRNA | . | . | . | . | . |
| XLOC_075421 | chr7:20368217-20369253 | - | 1.00623 | 1.21592 | LincRNA | ENSG00000271133.1 | CTA-293F17.1 | . | . | . |
| XLOC_075501 | chr7:22927739-22944037 | - | 0.247121 | 1.12287 | LincRNA | ENSG00000235664.1 | AC005682.8 | . | . | . |
| XLOC_075605 | chr7:25627606-25790627 | - | 4.53863 | 6.0714 | LincRNA | ENSG00000223561.2 | AC003090.1 | . | . | . |
| XLOC_076037 | chr7:41676661-41715590 | - | 0.813931 | 3.40018 | LincRNA | . | . | . | . | . |
| XLOC_076041 | chr7:41743563-41750728 | - | 0.594615 | 2.99909 | LincRNA | ENSG00000224116.2 | INHBA-AS1 | . | . | . |
| XLOC_076113 | chr7:43999383-44027526 | - | 1.18087 | 0.899493 | Pseudogene | ENSG00000241057.1 | AC004985.12 | . | . | . |
| XLOC_076129 | chr7:44027586-44058820 | - | 5.87735 | 3.76134 | Pseudogene | ENSG00000272655.1 | POLR2J4 | . | . | . |
| XLOC_076155 | chr7:45022628-45026250 | - | 3.89733 | 9.6932 | LincRNA | ENSG00000232956.4 | SNHG15 | . | . | . |
| XLOC_076162 | chr7:45763208-45808593 | - | 5.51396 | 4.27723 | Pseudogene | ENSG00000214765.4 | SEPT7P2 | . | . | . |
| XLOC_076315 | chr7:65888290-66057375 | - | 17.5293 | 18.5862 | Pseudogene | ENSG00000232491.1 | RP4-756H11.1 | XLOC_006133 | linc-KCTD7-2 | . |
| XLOC_076324 | chr7:65888290-66057375 | - | 0.789126 | 1.42484 | LincRNA | ENSG00000223473.1 | GS1-124K5.3 | XLOC_006133 | linc-KCTD7-2 | . |
| XLOC_076383 | chr7:66756141-66767397 | - | 1.01822 | 0.590946 | Pseudogene | ENSG00000067601.6 | PMS2P4 | . | . | . |
| XLOC_076426 | chr7:72474155-72476485 | - | 6.57249 | 3.53441 | Pseudogene | ENSG00000174353.13 | STAG3L3 | . | . | . |
| XLOC_076468 | chr7:74298021-74304787 | - | 2.051 | 1.13982 | Pseudogene | ENSG00000160828.13 | STAG3L2 | . | . | . |
| XLOC_076500 | chr7:75608764-75623961 | - | 5.30147 | 4.89093 | LincRNA | ENSG00000189077.6 | TMEM120A | . | . | . |
| XLOC_076556 | chr7:77312336-77325581 | - | 1.65519 | 1.01136 | LincRNA | ENSG00000214293.4 | RSBN1L-AS1 | . | . | . |
| XLOC_076579 | chr7:80553659-80558813 | - | 0.0868848 | 1.37605 | LincRNA | . | . | . | . | . |
| XLOC_076672 | chr7:86954620-86974850 | - | 10.5504 | 9.41238 | LincRNA | ENSG00000182165.13 | TP53TG1 | XLOC_006516 | linc-C7orf23 | . |
| XLOC_076702 | chr7:89945470-89950267 | - | 0.0219028 | 1.8159 | LincRNA | ENSG00000225498.1 | AC002064.5 | . | . | . |
| XLOC_076808 | chr7:93904914-94021537 | - | 3.31905 | 1.68296 | LincRNA | . | . | XLOC_006525 | linc-BET1-1 | eRNA |
| XLOC_076905 | chr7:97595345-97601668 | - | 4.90936 | 3.3489 | Pseudogene | ENSG00000243554.1 | AC004967.7 | . | . | . |
| XLOC_076954 | chr7:99927460-99933030 | - | 2.7278 | 2.51092 | Pseudogene | ENSG00000078319.7 | PMS2P1 | . | . | . |
| XLOC_076969 | chr7:100657663-100662204 | - | 0.416277 | 2.73196 | Antisense | ENSG00000227053.1 | RP11-395B7.4 | . | . | . |
| XLOC_076991 | chr7:102895256-102915641 | - | 1.41994 | 1.07585 | Pseudogene | ENSG00000170629.10 | DPY19L2P2 | . | . | . |
| XLOC_077041 | chr7:104533967-104553691 | - | 0.12111 | 1.17321 | Antisense | . | . | . | . | . |
| XLOC_077050 | chr7:104556361-104561443 | - | 0.0840428 | 1.12243 | LincRNA | ENSG00000225329.1 | RP11-325F22.5 | . | . | . |
| XLOC_077066 | chr7:104594167-104646316 | - | 3.47473 | 8.59256 | LincRNA | ENSG00000228393.3 | LINC01004 | XLOC_006195 | linc-MLL5 | . |
| XLOC_077102 | chr7:107380222-107402615 | - | 0.423378 | 1.00545 | Antisense | ENSG00000241764.3 | AC002467.7 | . | . | . |
| XLOC_077302 | chr7:129244383-129250779 | - | 1.12151 | 0.604904 | LincRNA | ENSG00000273329.1 | RP11-448A19.1 | XLOC_006604 | linc-TNPO3-3 | . |
| XLOC_077333 | chr7:130546147-130554024 | - | 1.04399 | 0.879461 | LincRNA | ENSG00000226380.3 | MIR29B1 | . | . | . |
| XLOC_077337 | chr7:130557540-130794735 | - | 11.7536 | 15.9649 | LincRNA | ENSG00000226380.3 | MIR29B1 | XLOC_006253 | linc-MKLN1-1 | eRNA |
| XLOC_077338 | chr7:130557540-130794735 | - | 1.92083 | 3.25467 | LincRNA | ENSG00000226380.3 | MIR29B1 | XLOC_006253 | linc-MKLN1-1 | eRNA |
| XLOC_077387 | chr7:134102965-134115130 | - | 1.60511 | 1.93149 | LincRNA | ENSG00000273297.1 | RP11-38M8.1 | . | . | . |
| XLOC_077553 | chr7:143075825-143078109 | - | 0.993009 | 1.39788 | LincRNA | ENSG00000232533.1 | AC093673.5 | . | . | . |
| XLOC_077632 | chr7:149169675-149321738 | - | 2.24001 | 2.73096 | Pseudogene | ENSG00000133624.9 | ZNF767 | XLOC_006288 | linc-KRBA1-1 | . |
| XLOC_077633 | chr7:149169675-149321738 | - | 2.45008 | 2.15159 | Pseudogene | ENSG00000133624.9 | ZNF767 | XLOC_006288 | linc-KRBA1-1 | . |
| XLOC_077659 | chr7:149169675-149321738 | - | 1.99376 | 1.23121 | Pseudogene | ENSG00000133624.9 | ZNF767 | XLOC_006288 | linc-KRBA1-1 | . |
| XLOC_077713 | chr7:155020985-155022081 | - | 1.80026 | 2.34201 | LincRNA | . | . | . | . | . |
| XLOC_077793 | chr7_gl000195_random:43008-86678 | - | 5.49704 | 7.11898 | LincRNA | . | . | XLOC_014513 | - | . |
| XLOC_077828 | chr8:2584508-2692093 | + | 3.97054 | 1.76098 | LincRNA | ENSG00000253853.1 | GS1-57L11.1 | XLOC_006980 | linc-ERICH1-9 | . |
| XLOC_078057 | chr8:11190195-11197344 | + | 0.809314 | 1.19933 | Pseudogene | ENSG00000154316.10 | TDH | XLOC_006710 | linc-BLK | . |
| XLOC_078095 | chr8:12294697-12458905 | + | 5.01282 | 4.40288 | LincRNA | ENSG00000270154.1 | RP11-419I17.1 | XLOC_006721 | linc-C8orf79-2 | . |
| XLOC_078180 | chr8:17658850-17679894 | + | 10.8838 | 1.93975 | LincRNA | ENSG00000253944.1 | RP11-156K13.1 | XLOC_006726 | linc-PCM1 | . |
| XLOC_078207 | chr8:17942203-17953412 | + | 2.11208 | 2.35613 | Antisense | ENSG00000245281.2 | CTD-2547L16.1 | . | . | . |
| XLOC_078336 | chr8:27558697-27566722 | + | 1.02445 | 0.732292 | LincRNA | . | . | . | . | . |
| XLOC_078384 | chr8:30601675-30624719 | + | 15.2069 | 13.6523 | Pseudogene | ENSG00000271081.1 | RP11-465K4.4 | . | . | . |
| XLOC_078394 | chr8:32785488-32907849 | + | 1.7284 | 1.00417 | LincRNA | ENSG00000247134.2 | RP11-11N9.4 | . | . | . |
| XLOC_078595 | chr8:52811904-52859748 | + | 1.14712 | 1.5229 | LincRNA | ENSG00000228801.5 | RP11-110G21.1 | . | . | . |
| XLOC_078680 | chr8:58658687-58661025 | + | 0.935755 | 1.55023 | LincRNA | ENSG00000253322.1 | RP11-388G22.1 | XLOC_006809 | linc-FAM110B-1 | . |
| XLOC_078796 | chr8:77318806-77345905 | + | 0.504881 | 4.85246 | LincRNA | ENSG00000253661.1 | ZFHX4-AS1 | XLOC_006850 | linc-ZFHX4-1 | . |
| XLOC_078832 | chr8:79717154-79798424 | + | 0.148547 | 34.5858 | LincRNA | . | . | . | . | . |
| XLOC_079015 | chr8:95566033-95582584 | + | 3.57365 | 0.630707 | Pseudogene | ENSG00000253175.1 | RP11-267M23.6 | . | . | . |
| XLOC_079128 | chr8:103817069-103822627 | + | 0.847878 | 1.14272 | LincRNA | ENSG00000253669.3 | KB-1732A1.1 | XLOC_006894 | linc-ATP6V1C1-3 | . |
| XLOC_079477 | chr8:129009511-129027298 | + | 1.73214 | 1.25056 | LincRNA | ENSG00000249859.3 | PVT1 | . | . | . |
| XLOC_079655 | chr8:157448-197313 | - | 3.00924 | 2.82763 | Pseudogene | ENSG00000223508.5 | RPL23AP53 | . | . | . |
| XLOC_079748 | chr8:7182277-7212817 | - | 2.16535 | 0.705585 | LincRNA | ENSG00000215374.5 | FAM66B | . | . | . |
| XLOC_079884 | chr8:19084676-19093127 | - | 0.758782 | 6.00324 | LincRNA | ENSG00000253280.1 | RP11-618M23.4 | . | . | . |
| XLOC_079964 | chr8:25036190-25041997 | - | 1.5365 | 1.13979 | LincRNA | . | . | . | . | . |
| XLOC_080060 | chr8:37260657-37351452 | - | 3.50935 | 4.80532 | LincRNA | ENSG00000253161.1 | RP11-150O12.1 | XLOC_007053 | linc-DUSP26-5 | . |
| XLOC_080204 | chr8:54427503-54436571 | - | 1.17868 | 2.48378 | LincRNA | ENSG00000254204.1 | RP11-400K9.3 | XLOC_007085 | linc-OPRK1-4 | . |
| XLOC_080240 | chr8:57406928-57472411 | - | 17.3129 | 12.5698 | LincRNA | ENSG00000254254.1 | RP11-17A4.2 | XLOC_007088 | linc-PENK-2 | . |
| XLOC_080381 | chr8:67123874-67170360 | - | 1.84358 | 1.47781 | LincRNA | . | . | XLOC_007110 | linc-CRH-4 | . |
| XLOC_080407 | chr8:67834228-67837824 | - | 252.862 | 230.258 | Antisense | ENSG00000245910.4 | SNHG6 | XLOC_006829 | linc-CSPP1 | . |
| XLOC_080451 | chr8:74153616-74162569 | - | 0.127555 | 1.61941 | LincRNA | . | . | . | . | . |
| XLOC_080608 | chr8:90570147-90627831 | - | 1.81308 | 17.529 | LincRNA | ENSG00000251136.4 | RP11-37B2.1 | . | . | . |
| XLOC_080615 | chr8:90627962-90765918 | - | 1.8534 | 11.0078 | LincRNA | ENSG00000251136.4 | RP11-37B2.1 | . | . | . |
| XLOC_080727 | chr8:92080152-92082384 | - | 24.5502 | 24.2617 | LincRNA | ENSG00000253738.1 | GS1-251I9.4 | . | . | . |
| XLOC_080861 | chr8:97081734-97091800 | - | 1.09986 | 0.139511 | Pseudogene | ENSG00000183663.6 | RP11-44N17.1 | . | . | . |
| XLOC_080992 | chr8:104258464-104344887 | - | 1.14634 | 1.02437 | LincRNA | ENSG00000247081.3 | RP11-318M2.2 | XLOC_006898 | linc-FZD6 | . |
| XLOC_081149 | chr8:115806280-115810438 | - | 1.20303 | 0.295534 | LincRNA | . | . | . | . | . |
| XLOC_081228 | chr8:117266655-117416017 | - | 1.47986 | 2.32415 | LincRNA | ENSG00000249917.2 | LINC00536 | . | . | . |
| XLOC_081334 | chr8:118691106-118725932 | - | 0.901304 | 1.8549 | LincRNA | . | . | XLOC_007189 | linc-RAD21-3 | . |
| XLOC_081351 | chr8:118744509-118758502 | - | 0.909276 | 1.50222 | LincRNA | . | . | XLOC_007191 | linc-RAD21-4 | . |
| XLOC_081489 | chr8:135796359-135817109 | - | 1.2222 | 0.496301 | LincRNA | ENSG00000259820.1 | AC083843.1 | . | . | . |
| XLOC_081536 | chr8:143746920-143756249 | - | 1.26384 | 0.221449 | Antisense | . | . | . | . | . |
| XLOC_081550 | chr8:144098310-144099621 | - | 2.48307 | 0.638574 | Antisense | ENSG00000247317.3 | RP11-273G15.2 | . | . | . |
| XLOC_081552 | chr8:144362784-144364657 | - | 1.10333 | 1.44529 | LincRNA | ENSG00000253716.1 | RP13-582O9.5 | XLOC_007249 | linc-LY6H | . |
| XLOC_081624 | chr8:146198979-146228279 | - | 6.01967 | 4.39062 | Pseudogene | ENSG00000196922.6 | ZNF252P | . | . | . |
| XLOC_081689 | chr9:3632466-3652163 | + | 1.49059 | 1.06954 | LincRNA | ENSG00000232104.2 | RP11-509J21.1 | . | . | . |
| XLOC_081703 | chr9:3667846-3690724 | + | 1.07627 | 1.4725 | LincRNA | ENSG00000226669.2 | RP11-509J21.4 | XLOC_007633 | linc-RFX3 | . |
| XLOC_081715 | chr9:3734567-3762403 | + | 0.504447 | 1.45708 | LincRNA | . | . | . | . | . |
| XLOC_081732 | chr9:3781364-3802716 | + | 0.164178 | 1.63371 | LincRNA | . | . | . | . | . |
| XLOC_081892 | chr9:17018335-17034087 | + | 1.78531 | 0.619006 | LincRNA | . | . | . | . | eRNA |
| XLOC_081995 | chr9:21682903-21689760 | + | 10.5986 | 93.1486 | LincRNA | . | . | . | . | eRNA |
| XLOC_082004 | chr9:22029519-22049339 | + | 1.53927 | 0.927605 | LincRNA | ENSG00000240498.2 | CDKN2B-AS1 | . | . | . |
| XLOC_082021 | chr9:22097053-22112339 | + | 1.38722 | 1.31975 | LincRNA | ENSG00000240498.2 | CDKN2B-AS1 | . | . | . |
| XLOC_082040 | chr9:22238152-22304481 | + | 2.21799 | 6.18761 | LincRNA | . | . | . | . | . |
| XLOC_082183 | chr9:22723322-22767588 | + | 1.00729 | 0.746879 | LincRNA | ENSG00000224549.1 | RP11-370B11.3 | . | . | . |
| XLOC_082273 | chr9:33178895-33179959 | + | 2.0349 | 3.88719 | LincRNA | ENSG00000233554.1 | RP11-326F20.5 | . | . | . |
| XLOC_082370 | chr9:37079993-37090745 | + | 11.6107 | 14.0455 | LincRNA | ENSG00000233137.2 | RP11-220I1.1 | . | . | . |
| XLOC_082596 | chr9:73216375-73219498 | + | 1.40118 | 1.67997 | Antisense | . | . | . | . | eRNA |
| XLOC_082648 | chr9:79130366-79180730 | + | 2.28541 | 2.69834 | Pseudogene | ENSG00000219149.4 | RP11-214N16.2 | XLOC_007415 | linc-FOXB2-2 | . |
| XLOC_082669 | chr9:79220769-79251773 | + | 1.92348 | 1.07174 | Antisense | . | . | . | . | . |
| XLOC_082711 | chr9:88420897-88466140 | + | 1.15687 | 0.857481 | Pseudogene | ENSG00000165121.10 | RP11-213G2.3 | . | . | . |
| XLOC_082737 | chr9:89366247-89373190 | + | 1.0812 | 0.965801 | LincRNA | . | . | XLOC_007437 | linc-C9orf170-2 | . |
| XLOC_082745 | chr9:89562974-89604199 | + | 1.43638 | 0.908906 | LincRNA | ENSG00000226237.1 | RP11-276H19.1 | XLOC_007438 | linc-C9orf170-1 | . |
| XLOC_082809 | chr9:93759915-93764094 | + | 0.532234 | 1.42824 | LincRNA | ENSG00000260454.1 | RP11-367F23.2 | . | . | . |
| XLOC_082852 | chr9:96734176-96740453 | + | 1.40909 | 1.11874 | LincRNA | . | . | . | . | . |
| XLOC_082869 | chr9:96928504-96963227 | + | 2.93172 | 2.14464 | LincRNA | ENSG00000269929.1 | RP11-2B6.2 | . | . | eRNA |
| XLOC_082895 | chr9:97440530-97444298 | + | 1.77993 | 0.250654 | LincRNA | . | . | . | . | . |
| XLOC_082902 | chr9:97853658-97885336 | + | 3.4768 | 1.17906 | Antisense | ENSG00000229065.1 | RP11-80I15.4 | . | . | . |
| XLOC_082934 | chr9:99449337-99491109 | + | 1.48946 | 0.823042 | LincRNA | ENSG00000224848.1 | RP11-535M15.1 | . | . | . |
| XLOC_083136 | chr9:113391589-113404403 | + | 0.170879 | 3.26707 | LincRNA | . | . | . | . | . |
| XLOC_083238 | chr9:116169513-116172955 | + | 1.93078 | 1.81859 | Antisense | . | . | . | . | . |
| XLOC_083259 | chr9:117366153-117366631 | + | 1.14595 | 1.75801 | LincRNA | . | . | . | . | . |
| XLOC_083355 | chr9:123555846-123562488 | + | 2.11108 | 1.50043 | Pseudogene | ENSG00000214654.5 | RP11-27I1.4 | XLOC_007549 | linc-CEP110-2 | . |
| XLOC_083360 | chr9:123577184-123639478 | + | 1.1805 | 0.705954 | LincRNA | ENSG00000226752.3 | PSMD5-AS1 | . | . | . |
| XLOC_083544 | chr9:132092284-132104440 | + | 2.78935 | 2.80042 | LincRNA | ENSG00000233901.1 | RP11-65J3.1 | XLOC_007865 | linc-IER5L-3 | . |
| XLOC_083551 | chr9:132251004-132267596 | + | 6.5038 | 4.67258 | LincRNA | ENSG00000204054.7 | LINC00963 | XLOC_007868 | linc-IER5L-2 | eRNA |
| XLOC_083563 | chr9:132337490-132343755 | + | 1.60087 | 1.12591 | LincRNA | ENSG00000227619.1 | RP11-492E3.2 | . | . | . |
| XLOC_083608 | chr9:136890595-136916855 | + | 2.37088 | 1.56398 | Antisense | ENSG00000235106.4 | LINC00094 | . | . | . |
| XLOC_083633 | chr9:138799751-138812960 | + | 2.51105 | 2.37094 | LincRNA | ENSG00000238058.1 | RP11-432J22.2 | . | . | . |
| XLOC_083702 | chr9:2506424-2523120 | - | 1.86805 | 2.23558 | LincRNA | ENSG00000231052.1 | RP11-91N2.3 | . | . | . |
| XLOC_083766 | chr9:4676485-4679479 | - | 3.26226 | 2.64078 | LincRNA | ENSG00000273061.1 | RP11-6J24.6 | . | . | . |
| XLOC_083772 | chr9:4880133-4887721 | - | 0.846059 | 1.25279 | Antisense | . | . | . | . | . |
| XLOC_083786 | chr9:5576765-5628940 | - | 2.26581 | 1.33872 | LincRNA | ENSG00000231509.1 | RP11-574F11.3 | . | . | . |
| XLOC_084130 | chr9:38069338-38075256 | - | 2.47273 | 1.39931 | LincRNA | . | . | XLOC_007686 | linc-SHB | . |
| XLOC_084141 | chr9:41953960-41956275 | - | 1.16537 | 0.911941 | Pseudogene | ENSG00000229273.1 | RP11-104G3.2 | XLOC_007690 | linc-FAM75A7-3 | . |
| XLOC_084181 | chr9:66830734-66863336 | - | 2.63446 | 1.48806 | Pseudogene | ENSG00000270092.2 | RP11-318K12.3 | . | . | . |
| XLOC_084184 | chr9:67252645-67289861 | - | 1.18823 | 0.708812 | Pseudogene | ENSG00000186466.4 | AQP7P1 | XLOC_007374 | linc-ANKRD20A1-4 | . |
| XLOC_084202 | chr9:67325416-67340112 | - | 17.9339 | 16.9841 | Pseudogene | ENSG00000237238.2 | BMS1P10 | . | . | . |
| XLOC_084219 | chr9:68433479-68440347 | - | 1.92379 | 0.632907 | Pseudogene | ENSG00000215548.2 | RP11-764K9.4 | . | . | . |
| XLOC_084523 | chr9:94289970-94290507 | - | 0 | 1.24414 | LincRNA | . | . | . | . | . |
| XLOC_084714 | chr9:99917560-99950861 | - | 2.2104 | 1.67164 | LincRNA | ENSG00000203279.3 | RP11-498P14.5 | . | . | . |
| XLOC_084726 | chr9:99973695-100013887 | - | 4.78715 | 2.22775 | Antisense | ENSG00000203279.3 | RP11-498P14.5 | XLOC_007483 | linc-C9orf174 | . |
| XLOC_084827 | chr9:102567864-102574135 | - | 2.13478 | 1.72994 | LincRNA | ENSG00000237461.1 | RP11-554F20.1 | XLOC_007808 | linc-ALG2-5 | . |
| XLOC_085072 | chr9:115759145-115774647 | - | 3.42085 | 2.63179 | LincRNA | ENSG00000228623.2 | ZNF883 | . | . | . |
| XLOC_085151 | chr9:118016154-118178107 | - | 0.910252 | 5.42524 | Antisense | ENSG00000234692.1 | RP11-445L6.3 | . | . | . |
| XLOC_085235 | chr9:118402663-118431095 | - | 0.274163 | 2.04231 | LincRNA | . | . | XLOC_007533 | linc-PAPPA-2 | eRNA |
| XLOC_085275 | chr9:120602610-120605604 | - | 0.178086 | 1.68919 | LincRNA | ENSG00000231901.1 | RP11-281A20.1 | XLOC_007537 | linc-CEP110-15 | . |
| XLOC_085308 | chr9:123714543-123837171 | - | 7.94983 | 4.78891 | LincRNA | . | . | . | . | . |
| XLOC_085571 | chr9:139618805-139622634 | - | 3.12353 | 2.86443 | LincRNA | ENSG00000233016.2 | SNHG7 | . | . | . |
| XLOC_085680 | chrUn_gl000218:46649-97434 | - | 1.23193 | 1.68198 | LincRNA | . | . | . | . | . |
| XLOC_085733 | chrUn_gl000219:51697-99642 | - | 12.7079 | 9.98259 | LincRNA | . | . | . | . | . |
| XLOC_085801 | chrUn_gl000241:14838-20767 | - | 17.388 | 22.7865 | LincRNA | . | . | . | . | . |
| XLOC_085803 | chrUn_gl000241:22263-36882 | - | 8.59827 | 8.53115 | LincRNA | . | . | . | . | . |
| XLOC_086004 | chrX:23093701-23096498 | + | 39.9562 | 38.5606 | Pseudogene | ENSG00000174028.6 | FAM3C2 | . | . | . |
| XLOC_086038 | chrX:30648241-30649098 | + | 2.59674 | 1.89134 | Pseudogene | ENSG00000232368.1 | FTLP2 | . | . | . |
| XLOC_086107 | chrX:45212810-45384096 | + | 2.48486 | 2.36688 | LincRNA | ENSG00000229563.2 | RP11-245M24.1 | XLOC_007966 | linc-ZNF673-4 | eRNA |
| XLOC_086137 | chrX:46359171-46408474 | + | 0.971689 | 1.82308 | Antisense | ENSG00000230844.2 | ZNF674-AS1 | XLOC_007970 | linc-CHST7 | . |
| XLOC_086227 | chrX:53123359-53200164 | + | 1.4098 | 0.302868 | LincRNA | ENSG00000234019.1 | RP11-236P24.3 | XLOC_008171 | linc-FAM156A-1 | . |
| XLOC_086448 | chrX:56753678-56846325 | + | 6.46517 | 5.6669 | Pseudogene | ENSG00000237748.1 | UQCRBP1 | . | . | . |
| XLOC_086562 | chrX:64271211-64589035 | + | 2.90949 | 3.05813 | Pseudogene | ENSG00000225569.1 | CCT4P2 | XLOC_008001 | linc-ZC3H12B-1 | . |
| XLOC_086774 | chrX:73164154-73229819 | + | 7.194 | 6.89851 | LincRNA | ENSG00000228906.1 | RP13-216E22.4 | XLOC_008186 | linc-NAP1L2-3 | . |
| XLOC_086823 | chrX:74743111-74824842 | + | 1.26367 | 0.311043 | Pseudogene | ENSG00000237089.2 | PCNPP4 | . | . | . |
| XLOC_087204 | chrX:102023833-102025316 | + | 2.62547 | 1.71925 | LincRNA | ENSG00000223546.2 | LINC00630 | . | . | . |
| XLOC_087297 | chrX:102081667-102438706 | + | 1.34138 | 1.00696 | LincRNA | . | . | XLOC_008033 | linc-BEX4 | . |
| XLOC_087300 | chrX:102081667-102438706 | + | 1.01161 | 0 | LincRNA | . | . | XLOC_008033 | linc-BEX4 | . |
| XLOC_087305 | chrX:102659744-102754728 | + | 3.66548 | 1.60161 | LincRNA | . | . | . | . | . |
| XLOC_087528 | chrX:119802396-119852057 | + | 0.394559 | 1.55699 | LincRNA | . | . | . | . | . |
| XLOC_087536 | chrX:119868870-119873534 | + | 0.337175 | 1.45772 | LincRNA | . | . | . | . | . |
| XLOC_088209 | chrX:45598253-45632802 | - | 18.5932 | 27.9604 | LincRNA | ENSG00000270069.1 | RP6-99M1.2 | XLOC_008149 | linc-CXorf36-1 | eRNA |
| XLOC_088213 | chrX:45659194-45666681 | - | 1.01288 | 1.90185 | LincRNA | . | . | . | . | . |
| XLOC_088217 | chrX:45673569-45710969 | - | 3.51284 | 3.77219 | LincRNA | ENSG00000231566.1 | RP5-1158E12.3 | . | . | . |
| XLOC_088388 | chrX:56736141-56740785 | - | 0.154149 | 1.90902 | LincRNA | . | . | . | . | . |
| XLOC_088534 | chrX:62646398-62780975 | - | 7.6923 | 4.78962 | LincRNA | . | . | XLOC_008174 | linc-SPIN4 | . |
| XLOC_088688 | chrX:70293193-70315602 | - | 7.08662 | 0 | LincRNA | . | . | . | . | . |
| XLOC_088713 | chrX:73040530-73063124 | - | 1.85109 | 2.12314 | LincRNA | ENSG00000229807.5 | XIST | XLOC_008015 | linc-ZCCHC13-2 | . |
| XLOC_088755 | chrX:73371685-73382871 | - | 1.21324 | 0.848348 | LincRNA | ENSG00000228906.1 | RP13-216E22.4 | . | . | . |
| XLOC_088806 | chrX:74956551-74966986 | - | 12.4236 | 8.39045 | Pseudogene | ENSG00000215105.3 | TTC3P1 | . | . | . |
| XLOC_088851 | chrX:77785222-77914960 | - | 7.68217 | 2.00721 | LincRNA | . | . | . | . | . |
| XLOC_088883 | chrX:79544414-79565735 | - | 0.742089 | 1.64948 | Pseudogene | ENSG00000215104.4 | RP11-217H19.1 | . | . | . |
| XLOC_088937 | chrX:90669904-90673852 | - | 5.64472 | 5.24215 | Pseudogene | ENSG00000236413.2 | RP13-24G8.2 | . | . | . |
| XLOC_089234 | chrX:119371612-119379061 | - | 1.01763 | 0.513717 | LincRNA | ENSG00000228139.1 | GS1-421I3.4 | XLOC_008225 | linc-RHOXF1-4 | . |
| XLOC_089279 | chrX:124117144-124338220 | - | 1.34536 | 1.0434 | Pseudogene | ENSG00000232599.1 | RP1-161N10.1 | . | . | . |
| XLOC_089467 | chrX:128809663-128812329 | - | 1.06711 | 0.273599 | LincRNA | . | . | XLOC_008233 | linc-APLN-1 | . |
| XLOC_089643 | chrX:153652728-153656821 | - | 4.87624 | 4.23202 | Pseudogene | ENSG00000197180.1 | BX936347.1 | . | . | . |
| XLOC_089773 | chrY:7142092-7245093 | + | 1.48995 | 1.53364 | Pseudogene | ENSG00000099725.10 | PRKY | . | . | . |
| XLOC_089855 | chrY:14774177-14972768 | + | 145.113 | 109.438 | LincRNA | ENSG00000233864.3 | TTTY15 | XLOC_008323 | linc-AMELY-6 | . |
| XLOC_089997 | chrY:21729211-21765735 | + | 6.73077 | 5.46656 | Pseudogene | ENSG00000131002.7 | TXLNG2P | . | . | . |
| XLOC_090027 | chrY:28810932-28819127 | + | 3.50981 | 2.05932 | LincRNA | . | . | . | . | . |
| XLOC_090088 | chrY:18750592-18751758 | - | 0.0289092 | 1.07095 | LincRNA | . | . | . | . | . |
| XLOC_090089 | chrY:21034404-21239256 | - | 1.62739 | 0.866387 | LincRNA | ENSG00000176728.3 | TTTY14 | XLOC_008326 | linc-HSFY2 | . |
| XLOC_090110 | chrY:21034404-21239256 | - | 1.25342 | 0.685891 | LincRNA | ENSG00000176728.3 | TTTY14 | XLOC_008326 | linc-HSFY2 | . |
| XLOC_090156 | chrY:21034404-21239256 | - | 2.03357 | 1.49612 | LincRNA | ENSG00000176728.3 | TTTY14 | XLOC_008326 | linc-HSFY2 | . |
| XLOC_090236 | chrY:22628319-22631787 | - | 1.98111 | 2.92555 | LincRNA | ENSG00000229236.1 | TTTY10 | . | . | . |
| XLOC_090248 | chrY:22669174-22689226 | - | 1.64792 | 0.996615 | LincRNA | ENSG00000229236.1 | TTTY10 | XLOC_008328 | linc-KDM5D-2 | . |
