# Supplementary material for: Long Intergenic Noncoding RNAs Mediate the Human Chondrocyte Inflammatory Response and Are Differentially Expressed in Osteoarthritis Cartilage
Source: Arthritis Rheumatol. 2016 Mar 28;68(4):845–56. doi: 10.1002/art.39520 (PMC4950001; doi:10.1002/art.39520)
Supplement: Supplementary file 7 — Supplementary Table 4 [file ART-68-845-s007.docx]

Supplementary Table 4

| **LncRNA number** | **Position** | **Strand** | **Control (FPKM)** | **IL1b (FPKM)** | **Absolute Change (FPKM)** | **Log2 (fold Change)** | **p-value** | **q-value** | **LncRNA Class** | **GenCode Number** | **Gencode Name** | **Human Body Map Number** | **Human Body Map Name** | **Map Name** |
| --- | --- | --- | --- | --- | --- | --- | --- | --- | --- | --- | --- | --- | --- | --- |
| XLOC_070349 | chr6:51274648-51275317 | - | 56.0624 | 314.391 | 258.3286 | 2.48746 | 5.00E-05 | 0.000794074 | Pseudogene | ENSG00000232702.2 | RP3-437C15.1 | . | . |  |
| XLOC_028931 | chr16:56651381-56652154 | + | 47.386 | 133.375 | 85.989 | 1.49295 | 0.0006 | 0.00598863 | Pseudogene | ENSG00000260549.1 | MT1L | . | . |  |
| XLOC_081995 | chr9:21682903-21689760 | + | 10.5986 | 93.1486 | 82.55 | 3.13567 | 5.00E-05 | 0.000794074 | LincRNA | . | . | . | . |  |
| XLOC_043077 | chr6:143267747-143280112 | - | 0.959518 | 59.511 | 58.551482 | 5.9547 | 5.00E-05 | 0.000673267 | LincRNA | ENSG00000232618.1 | RP11-439L18.1 | - | - | HIVEP2 |
| XLOC_078832 | chr8:79717154-79798424 | + | 0.148547 | 34.5858 | 34.437253 | 7.86312 | 5.00E-05 | 0.000794074 | LincRNA | . | . | . | . | Linc-IL7 |
| XLOC_013921 | chr11:125962203-125978095 | + | 0.439307 | 23.7498 | 23.310493 | 5.75654 | 5.00E-05 | 0.000794074 | LincRNA | ENSG00000261257.1 | RP11-673E11.2 | XLOC_009320 | linc-FAM118B |  |
| XLOC_016045 | chr11:133890833-133917442 | - | 0.743462 | 19.3298 | 18.586338 | 4.70042 | 5.00E-05 | 0.000794074 | LincRNA | ENSG00000204241.3 | RP11-713P17.3 | XLOC_009341 | linc-JAM3-2 |  |
| XLOC_080608 | chr8:90570147-90627831 | - | 1.81308 | 17.529 | 15.71592 | 3.27323 | 5.00E-05 | 0.000794074 | LincRNA | ENSG00000251136.4 | RP11-37B2.1 | . | . |  |
| XLOC_047794 | chr21:26934114-26976653 | + | 0.266717 | 13.3313 | 13.064583 | 5.64336 | 5.00E-05 | 0.000794074 | Antisense | ENSG00000260583.1 | LINC00515 | XLOC_014038 | linc-TMPRSS15-16 |  |
| XLOC_061956 | chr5:64327144-64365959 | + | 3.81597 | 16.4397 | 12.62373 | 2.10706 | 5.00E-05 | 0.000794074 | LincRNA | . | . | XLOC_004407 | linc-PPWD1 |  |
| XLOC_065146 | chr5:90596973-90610312 | - | 1.254 | 11.9774 | 10.7234 | 3.25571 | 5.00E-05 | 0.000794074 | LincRNA | ENSG00000248323.1 | LUCAT1 | XLOC_004468 | linc-NR2F1-7 |  |
| XLOC_043698 | chr2:152127077-152196200 | - | 0.39188 | 10.9272 | 10.53532 | 4.80137 | 0.0003 | 0.00338689 | LincRNA | . | . | . | . |  |
| XLOC_018205 | chr12:102675067-102676048 | + | 0 | 10.4673 | 10.4673 | inf | 0.00035 | 0.00384313 | LincRNA | . | . | . | . |  |
| XLOC_063823 | chr5:159895208-159922844 | + | 0.0597088 | 9.34461 | 9.2849012 | 7.29005 | 5.00E-05 | 0.000794074 | LincRNA | ENSG00000253522.2 | MIR146A | . | . |  |
| XLOC_080615 | chr8:90627962-90765918 | - | 1.8534 | 11.0078 | 9.1544 | 2.57029 | 5.00E-05 | 0.000794074 | LincRNA | ENSG00000251136.4 | RP11-37B2.1 | . | . |  |
| XLOC_033343 | chr17:66686917-66691401 | - | 2.96227 | 11.0901 | 8.12783 | 1.9045 | 5.00E-05 | 0.000794074 | LincRNA | ENSG00000267659.1 | RP11-118B18.1 | . | . |  |
| XLOC_029874 | chr16:10522758-10592329 | - | 0.818083 | 8.20796 | 7.389877 | 3.3267 | 5.00E-05 | 0.000794074 | Antisense | . | . | . | . |  |
| XLOC_006031 | chr1:119349587-119409083 | - | 1.92565 | 9.23502 | 7.30937 | 2.26177 | 0.00675 | 0.0443608 | LincRNA | . | . | . | . |  |
| XLOC_027307 | chr15:38330295-38365459 | - | 0.53219 | 7.43766 | 6.90547 | 3.80483 | 5.00E-05 | 0.000794074 | LincRNA | ENSG00000259225.2 | RP11-1008C21.1 | XLOC_011213 | linc-SPRED1 |  |
| **LncRNA number** | **Position** | **Strand** | **Control (FPKM)** | **IL1b (FPKM)** | **Absolute Change (FPKM)** | **Log2 (fold Change)** | **p-value** | **q-value** | **LncRNA Class** | **GenCode Number** | **Gencode Name** | **Human Body Map Number** | **Human Body Map Name** | **Map Name** |
| XLOC_074956 | chr7:139953048-139957857 | + | 0.420158 | 6.3814 | 5.961242 | 3.92487 | 5.00E-05 | 0.000794074 | LincRNA | . | . | . | . |  |
| XLOC_076155 | chr7:45022628-45026250 | - | 3.89733 | 9.6932 | 5.79587 | 1.31448 | 0.0019 | 0.0151006 | LincRNA | ENSG00000232956.4 | SNHG15 | . | . |  |
| XLOC_001518 | chr1:89873116-89891927 | + | 0.58582 | 5.96536 | 5.37954 | 3.34808 | 5.00E-05 | 0.000794074 | Pseudogene | ENSG00000225492.2 | GBP1P1 | . | . |  |
| XLOC_077066 | chr7:104594167-104646316 | - | 3.47473 | 8.59256 | 5.11783 | 1.30619 | 0.00105 | 0.00919186 | LincRNA | ENSG00000228393.3 | LINC01004 | XLOC_006195 | linc-MLL5 |  |
| XLOC_074222 | chr7:98895298-98895600 | + | 0 | 5.10982 | 5.10982 | inf | 0.00025 | 0.00293431 | Pseudogene | ENSG00000002079.8 | MYH16 | . | . |  |
| XLOC_013478 | chr11:102251554-102261900 | + | 1.74929 | 6.39946 | 4.65017 | 1.87118 | 5.00E-05 | 0.000794074 | LincRNA | . | . | . | . |  |
| XLOC_007480 | chr1:219597501-219613213 | - | 2.04872 | 6.20003 | 4.15131 | 1.59755 | 5.00E-05 | 0.000794074 | LincRNA | ENSG00000230024.1 | RP11-95P13.1 | XLOC_000567 | linc-IARS2-2 |  |
| XLOC_072456 | chr6:160007870-160013184 | - | 0.0848209 | 3.7715 | 3.6866791 | 5.47458 | 5.00E-05 | 0.000794074 | LincRNA | ENSG00000237927.1 | RP3-393E18.2 | XLOC_005900 | linc-TAGAP-1 |  |
| XLOC_061422 | chr5:17604295-17606015 | + | 0.22736 | 3.83248 | 3.60512 | 4.07523 | 5.00E-05 | 0.000794074 | Pseudogene | ENSG00000249357.2 | RP11-432M8.8 | . | . |  |
| XLOC_003097 | chr1:186649747-186654651 | + | 0.743088 | 4.33362 | 3.590532 | 2.54397 | 5.00E-05 | 0.000794074 | LincRNA | ENSG00000273129.1 | RP5-973M2.2 | . | . | PACER |
| XLOC_005041 | chr1:65437492-65468317 | - | 1.2302 | 4.67309 | 3.44289 | 1.92548 | 5.00E-05 | 0.000794074 | Pseudogene | ENSG00000185031.6 | SLC2A3P2 | . | . |  |
| XLOC_061582 | chr5:35366494-35422974 | + | 0.172064 | 3.58667 | 3.414606 | 4.38163 | 5.00E-05 | 0.000794074 | LincRNA | . | . | . | . |  |
| XLOC_075204 | chr7:4684757-4701106 | - | 0.536025 | 3.80146 | 3.265435 | 2.82618 | 0.0002 | 0.00243059 | Antisense | . | . | . | . |  |
| XLOC_003348 | chr1:207540496-207549020 | + | 2.0128 | 5.19924 | 3.18644 | 1.36909 | 0.0008 | 0.00747734 | LincRNA | . | . | . | . |  |
| XLOC_018712 | chr12:9381199-9382950 | - | 0 | 3.01365 | 3.01365 | inf | 0.00095 | 0.00853248 | LincRNA | ENSG00000256427.1 | RP11-118B22.4 | . | . |  |
| XLOC_048827 | chr21:43470814-43475824 | - | 0.255665 | 3.23699 | 2.981325 | 3.66233 | 5.00E-05 | 0.000794074 | LincRNA | . | . | XLOC_013952 | linc-UMODL1-1 |  |
| XLOC_035007 | chr18:67332445-67392454 | - | 1.11129 | 4.03935 | 2.92806 | 1.86189 | 5.00E-05 | 0.000794074 | Antisense | ENSG00000266840.1 | RP11-543H23.2 | . | . |  |
| XLOC_006385 | chr1:153761245-153762791 | - | 0.0864682 | 2.95862 | 2.8721518 | 5.09661 | 0.00735 | 0.0475723 | LincRNA | . | . | . | . |  |
| XLOC_043678 | chr2:151560131-151567657 | - | 0.17628 | 3.03823 | 2.86195 | 4.10729 | 5.00E-05 | 0.000794074 | LincRNA | . | . | . | . |  |
| XLOC_048072 | chr21:43188194-43194760 | + | 0.23699 | 3.07771 | 2.84072 | 3.69896 | 0.0003 | 0.00338689 | LincRNA | . | . | XLOC_013950 | linc-UMODL1-2 |  |
| XLOC_028824 | chr16:31711899-31717488 | + | 1.42769 | 4.20848 | 2.78079 | 1.55962 | 0.0011 | 0.00955936 | LincRNA | ENSG00000261731.2 | CTD-2358C21.4 | . | . |  |
| XLOC_024596 | chr14:50500383-50507672 | - | 0.650167 | 3.29792 | 2.647753 | 2.34267 | 5.00E-05 | 0.000794074 | LincRNA | ENSG00000270788.1 | PDLIM1P1 | . | . |  |
| **LncRNA number** | **Position** | **Strand** | **Control (FPKM)** | **IL1b (FPKM)** | **Absolute Change (FPKM)** | **Log2 (fold Change)** | **p-value** | **q-value** | **LncRNA Class** | **GenCode Number** | **Gencode Name** | **Human Body Map Number** | **Human Body Map Name** | **Map Name** |
| XLOC_005753 | chr1:111905991-111911108 | - | 0 | 2.499 | 2.499 | inf | 5.00E-05 | 0.000794074 | Pseudogene | ENSG00000234020.1 | CHIAP3 | . | . |  |
| XLOC_067394 | chr6:35936578-35968897 | + | 0.218798 | 2.65398 | 2.435182 | 3.60049 | 5.00E-05 | 0.000794074 | Antisense | ENSG00000271304.1 | DPRXP2 | . | . |  |
| XLOC_061661 | chr5:41281482-41350879 | + | 0.138734 | 2.55131 | 2.412576 | 4.20084 | 0.0025 | 0.0188568 | Antisense | . | . | XLOC_004799 | linc-C6 |  |
| XLOC_076041 | chr7:41743563-41750728 | - | 0.594615 | 2.99909 | 2.404475 | 2.3345 | 0.00065 | 0.0064072 | LincRNA | ENSG00000224116.2 | INHBA-AS1 | . | . |  |
| XLOC_068456 | chr6:112337892-112353718 | + | 0 | 2.38924 | 2.38924 | inf | 5.00E-05 | 0.000794074 | LincRNA | . | . | . | . |  |
| XLOC_066290 | chr5:150348010-150377882 | - | 0.0239596 | 2.27183 | 2.2478704 | 6.56711 | 5.00E-05 | 0.000794074 | LincRNA | . | . | . | . |  |
| XLOC_031059 | chr17:14285012-14326180 | + | 0.0952417 | 2.30413 | 2.2088883 | 4.59648 | 0.00355 | 0.0255959 | LincRNA | ENSG00000230647.1 | AC022816.2 | XLOC_012116 | linc-ZNF286A-6 |  |
| XLOC_048345 | chr21:26796229-26825140 | - | 0.144383 | 2.3311 | 2.186717 | 4.01304 | 5.00E-05 | 0.000794074 | LincRNA | ENSG00000185433.4 | LINC00158 | . | . |  |
| XLOC_002240 | chr1:145382754-145383571 | + | 1.11593 | 3.29549 | 2.17956 | 1.56224 | 0.00685 | 0.0448682 | LincRNA | . | . | . | . |  |
| XLOC_048423 | chr21:28984539-29019990 | - | 0.0718199 | 2.23055 | 2.1587301 | 4.95687 | 0.0006 | 0.00598863 | LincRNA | ENSG00000234052.1 | AP001607.1 | XLOC_013896 | linc-USP16-9 |  |
| XLOC_034244 | chr18:53833409-53838003 | + | 0.0528565 | 2.20493 | 2.1520735 | 5.38251 | 0.00095 | 0.00853248 | LincRNA | ENSG00000267327.1 | CTD-2008L17.1 | XLOC_012687 | linc-WDR7-1 |  |
| XLOC_060842 | chr4:158573954-158598790 | - | 0.0131208 | 2.14144 | 2.1283192 | 7.35058 | 5.00E-05 | 0.000794074 | LincRNA | ENSG00000249275.1 | RP11-364P22.2 | XLOC_003759 | linc-TMEM144-1 |  |
| XLOC_071449 | chr6:113946487-113975857 | - | 0.0468708 | 2.17102 | 2.1241492 | 5.53354 | 0.00225 | 0.0172673 | LincRNA | ENSG00000230943.1 | RP11-367G18.1 | XLOC_005810 | linc-LAMA4-2 |  |
| XLOC_004949 | chr1:56880628-56925281 | - | 0.416454 | 2.46429 | 2.047836 | 2.56494 | 0.0007 | 0.00676896 | LincRNA | ENSG00000223956.1 | RP4-710M16.2 | XLOC_000214 | linc-PRKAA2-1 |  |
| XLOC_071797 | chr6:127175941-127215669 | - | 0.216655 | 2.24546 | 2.028805 | 3.37354 | 5.00E-05 | 0.000794074 | LincRNA | . | . | XLOC_005460 | linc-RSPO3 |  |
| XLOC_011641 | chr10:106110780-106113335 | - | 0.347492 | 2.32445 | 1.976958 | 2.74184 | 0.0002 | 0.00243059 | LincRNA | ENSG00000231233.1 | CCDC147-AS1 | XLOC_008937 | linc-ITPRIP-1 |  |
| XLOC_027305 | chr15:38330295-38365459 | - | 0.0983273 | 1.94247 | 1.8441427 | 4.30416 | 0.00055 | 0.00556313 | LincRNA | ENSG00000259225.2 | RP11-1008C21.1 | XLOC_011213 | linc-SPRED1 |  |
| XLOC_016326 | chr12:9855072-9866376 | + | 0.065488 | 1.888572 | 1.823084 | 4.76219 | 5.00E-05 | 0.000794074 | LincRNA | ENSG00000256582.1 | RP11-75L1.1 | . | . |  |
| XLOC_024539 | chr14:42056652-42075504 | - | 0.900455 | 2.70922 | 1.808765 | 1.58915 | 0.00075 | 0.00720477 | LincRNA | ENSG00000258636.1 | CTD-2298J14.2 | . | . |  |
| XLOC_018058 | chr12:90485171-90507675 | + | 0.0346874 | 1.83942 | 1.8047326 | 5.7287 | 5.00E-05 | 0.000794074 | LincRNA | ENSG00000257194.2 | RP11-567C2.1 | XLOC_009829 | linc-CLLU1-6 |  |
| XLOC_085235 | chr9:118402663-118431095 | - | 0.274163 | 2.04231 | 1.768147 | 2.8971 | 5.00E-05 | 0.000794074 | LincRNA | . | . | XLOC_007533 | linc-PAPPA-2 |  |
| XLOC_072067 | chr6:138175998-138186493 | - | 1.10512 | 2.8659 | 1.76078 | 1.37478 | 0.0027 | 0.0202072 | LincRNA | ENSG00000237499.2 | RP11-356I2.4 | XLOC_005479 | linc-TNFAIP3-1 |  |
| **LncRNA number** | **Position** | **Strand** | **Control (FPKM)** | **IL1b (FPKM)** | **Absolute Change (FPKM)** | **Log2 (fold Change)** | **p-value** | **q-value** | **LncRNA Class** | **GenCode Number** | **Gencode Name** | **Human Body Map Number** | **Human Body Map Name** | **Map Name** |
| XLOC_069077 | chr6:138014249-138023121 | + | 0.154571 | 1.85035 | 1.695779 | 3.58146 | 0.0034 | 0.0247429 | LincRNA | . | . | . | . |  |
| XLOC_009474 | chr10:104593023-104594458 | + | 0.384285 | 2.05774 | 1.673455 | 2.42081 | 0.0028 | 0.0208694 | Antisense | ENSG00000203886.4 | CYP17A1-AS1 | . | . |  |
| XLOC_001610 | chr1:96208581-96227515 | + | 1.2109 | 2.87229 | 1.66139 | 1.24612 | 0.0047 | 0.0325507 | LincRNA | ENSG00000271252.1 | RP11-286B14.2 | . | . |  |
| XLOC_026242 | chr15:45825678-45848355 | + | 0.0859807 | 1.72905 | 1.6430693 | 4.32983 | 5.00E-05 | 0.000794074 | LincRNA | ENSG00000259354.1 | RP11-519G16.3 | . | . |  |
| XLOC_034872 | chr18:53865316-53893620 | - | 0.117971 | 1.71416 | 1.596189 | 3.861 | 0.0005 | 0.00515642 | LincRNA | . | . | . | . |  |
| XLOC_024029 | chr14:94959361-94964321 | + | 0.122587 | 1.7013 | 1.578713 | 3.79477 | 0.00015 | 0.00193289 | Antisense | . | . | . | . |  |
| XLOC_054201 | chr3:40338409-40351031 | - | 0.947267 | 2.46003 | 1.512763 | 1.37683 | 0.00025 | 0.00293431 | LincRNA | . | . | . | . |  |
| XLOC_050403 | chr3:23710334-23711913 | + | 0.0888147 | 1.59485 | 1.5060353 | 4.16648 | 0.0056 | 0.037852 | LincRNA | . | . | . | . |  |
| XLOC_066990 | chr6:14704157-14709984 | + | 0.148749 | 1.6542 | 1.505451 | 3.47517 | 5.00E-05 | 0.000794074 | LincRNA | . | . | . | . |  |
| XLOC_000013 | chr1:998476-1001791 | + | 0.382595 | 1.88678 | 1.504185 | 2.30203 | 0.00345 | 0.0250103 | Pseudogene | ENSG00000217801.5 | RP11-465B22.3 | . | . |  |
| XLOC_058570 | chr4:174447778-174512356 | + | 0.359543 | 1.84638 | 1.486837 | 2.36046 | 0.00055 | 0.00556313 | Antisense | ENSG00000237125.4 | HAND2-AS1 | . | . |  |
| XLOC_081732 | chr9:3781364-3802716 | + | 0.164178 | 1.63371 | 1.469532 | 3.31482 | 0.0041 | 0.0289486 | LincRNA | . | . | . | . |  |
| XLOC_049693 | chr22:35588412-35627034 | - | 0 | 1.45129 | 1.45129 | inf | 0.00015 | 0.00193289 | Pseudogene | ENSG00000243453.1 | COX7BP1 | . | . |  |
| XLOC_066780 | chr6:7673545-7676259 | + | 0.166788 | 1.56789 | 1.401102 | 3.23274 | 0.0017 | 0.0136718 | LincRNA | . | . | . | . |  |
| XLOC_043002 | chr2:101339479-101359483 | - | 0.0800317 | 1.47531 | 1.3952783 | 4.20431 | 0.00385 | 0.0274185 | LincRNA | . | . | XLOC_001591 | linc-NPAS2-1 |  |
| XLOC_019309 | chr12:31267625-31270542 | - | 0 | 1.36645 | 1.36645 | inf | 5.00E-05 | 0.000794074 | Pseudogene | ENSG00000177359.13 | RP11-551L14.1 | . | . |  |
| XLOC_076579 | chr7:80553659-80558813 | - | 0.0868848 | 1.37605 | 1.2891652 | 3.98529 | 0.00015 | 0.00193289 | LincRNA | . | . | . | . |  |
| XLOC_023820 | chr14:73929077-73933619 | + | 0.284817 | 1.56993 | 1.285113 | 2.46259 | 5.00E-05 | 0.000794074 | Antisense | ENSG00000251393.3 | RP1-240K6.3 | XLOC_011068 | linc-NUMB |  |
| XLOC_042424 | chr2:64989523-64995661 | - | 0.244116 | 1.46934 | 1.225224 | 2.58953 | 0.00035 | 0.00384313 | LincRNA | . | . | . | . |  |
| XLOC_025981 | chr15:38656159-38665564 | + | 0.24804 | 1.40212 | 1.15408 | 2.49897 | 5.00E-05 | 0.000794074 | LincRNA | . | . | . | . |  |
| XLOC_031342 | chr17:29956710-29965823 | + | 0.0949079 | 1.20545 | 1.1105421 | 3.6669 | 0.00225 | 0.0172673 | LincRNA | ENSG00000266877.1 | RP1-41C23.1 | XLOC_012166 | linc-SUZ12-3 |  |
| XLOC_077041 | chr7:104533967-104553691 | - | 0.12111 | 1.17321 | 1.0521 | 3.27606 | 5.00E-05 | 0.000794074 | Antisense | . | . | . | . |  |
| XLOC_071433 | chr6:112605582-112610269 | - | 0.637486 | 1.67847 | 1.040984 | 1.39668 | 0.00115 | 0.00994997 | Pseudogene | ENSG00000216663.3 | RP11-506B6.5 | . | . |  |
| **LncRNA number** | **Position** | **Strand** | **Control (FPKM)** | **IL1b (FPKM)** | **Absolute Change (FPKM)** | **Log2 (fold Change)** | **p-value** | **q-value** | **LncRNA Class** | **GenCode Number** | **Gencode Name** | **Human Body Map Number** | **Human Body Map Name** | **Map Name** |
| XLOC_040838 | chr2:202835756-202845480 | + | 0 | 1.04083 | 1.04083 | inf | 0.0005 | 0.00515642 | LincRNA | . | . | . | . |  |
| XLOC_032065 | chr17:76840091-76843218 | + | 0.27879 | 1.31911 | 1.04032 | 2.24231 | 0.0001 | 0.00141323 | LincRNA | ENSG00000267355.2 | RPL9P29 | . | . |  |
| XLOC_077050 | chr7:104556361-104561443 | - | 0.0840428 | 1.12243 | 1.0383872 | 3.73936 | 0.0001 | 0.00141323 | LincRNA | ENSG00000225329.1 | RP11-325F22.5 | . | . |  |
| XLOC_041210 | chr2:225016338-225035395 | + | 0.15341 | 1.18994 | 1.03653 | 2.95543 | 0.004 | 0.028322 | Pseudogene | ENSG00000224826.1 | AC019109.1 | . | . |  |
| XLOC_072411 | chr6:159586993-159588613 | - | 0.305333 | 1.33782 | 1.032487 | 2.13143 | 0.00615 | 0.0409103 | LincRNA | ENSG00000233682.2 | RP11-13P5.2 | XLOC_005522 | linc-FNDC1-1 |  |
| XLOC_073505 | chr7:46072766-46076950 | + | 0 | 1.02754 | 1.02754 | inf | 5.00E-05 | 0.000794074 | LincRNA | . | . | . | . |  |
| XLOC_015977 | chr11:125793401-125800822 | - | 0.200828 | 1.2109 | 1.010072 | 2.59205 | 5.00E-05 | 0.000794074 | LincRNA | ENSG00000255027.2 | RP11-680F20.9 | XLOC_009594 | linc-PUS3-2 |  |
| XLOC_040968 | chr2:208104033-208113615 | + | 0.287034 | 1.26093 | 0.973896 | 2.13519 | 0.00065 | 0.0064072 | LincRNA | ENSG00000223725.2 | AC007879.5 | XLOC_002473 | linc-KLF7-2 |  |
| XLOC_009027 | chr10:77191281-77211897 | + | 0.72112 | 1.65377 | 0.93265 | 1.19745 | 0.0004 | 0.00425496 | LincRNA | ENSG00000236842.1 | RP11-399K21.10 | . | . |  |
| XLOC_064213 | chr5:16437064-16448804 | - | 0.586006 | 1.49028 | 0.904274 | 1.3466 | 0.0006 | 0.00598863 | LincRNA | ENSG00000249737.1 | RP1-167G20.2 | XLOC_004303 | linc-BASP1-3 |  |
| XLOC_075501 | chr7:22927739-22944037 | - | 0.247121 | 1.12287 | 0.875749 | 2.1839 | 5.00E-05 | 0.000794074 | LincRNA | ENSG00000235664.1 | AC005682.8 | . | . |  |
| XLOC_062280 | chr5:83877293-83899494 | + | 0.647917 | 1.36878 | 0.720863 | 1.07901 | 0.0033 | 0.0240693 | LincRNA | . | . | . | . |  |
| XLOC_021529 | chr13:92001273-92021299 | + | 0.696672 | 1.38164 | 0.684968 | 0.987827 | 0.0044 | 0.0307837 | LincRNA | ENSG00000215417.6 | MIR17HG | . | . |  |
| XLOC_047906 | chr21:35541900-35564339 | + | 1.0203 | 0.471983 | -0.548317 | -1.11218 | 0.0011 | 0.00955936 | LincRNA | ENSG00000227456.3 | LINC00310 | XLOC_013923 | linc-KCNE2-3 |  |
| XLOC_081489 | chr8:135796359-135817109 | - | 1.2222 | 0.496301 | -0.725899 | -1.30019 | 5.00E-05 | 0.000794074 | LincRNA | ENSG00000259820.1 | AC083843.1 | . | . |  |
| XLOC_044575 | chr2:190252753-190260012 | - | 1.05366 | 0.259826 | -0.793834 | -2.0198 | 0.0001 | 0.00141323 | LincRNA | . | . | XLOC_002433 | linc-COL5A2 |  |
| XLOC_038735 | chr2:95873143-95888872 | + | 1.56552 | 0.731502 | -0.834018 | -1.09771 | 0.00155 | 0.0125952 | LincRNA | ENSG00000233757.2 | AC092835.2 | . | . |  |
| XLOC_066163 | chr5:146556638-146563169 | - | 1.18843 | 0.294413 | -0.894017 | -2.01315 | 0.00375 | 0.0268414 | LincRNA | ENSG00000250343.1 | CTC-255N20.1 | XLOC_005046 | linc-PPP2R2B-2 |  |
| XLOC_061283 | chr5:9546406-9553232 | + | 1.56616 | 0.644405 | -0.921755 | -1.28119 | 0.0007 | 0.00676896 | LincRNA | ENSG00000250786.1 | SNHG18 | . | . |  |
| XLOC_013218 | chr11:87078182-87083856 | + | 1.00693 | 0 | -1.00693 | #NAME? | 5.00E-05 | 0.000794074 | LincRNA | . | . | . | . |  |
| XLOC_052429 | chr3:152203323-152205115 | + | 1.27059 | 0.259236 | -1.011354 | -2.29316 | 0.00095 | 0.00853248 | LincRNA | ENSG00000243305.1 | RP11-362A9.3 | . | . |  |
| XLOC_045353 | chr2:234774050-234777051 | - | 1.79592 | 0.714469 | -1.081451 | -1.32978 | 0.00295 | 0.0217724 | Pseudogene | ENSG00000224287.2 | MSL3P1 | . | . |  |
| XLOC_025813 | chr15:30395911-30402607 | + | 1.1037 | 0 | -1.1037 | #NAME? | 5.00E-05 | 0.000794074 | Pseudogene | ENSG00000178081.8 | ULK4P3 | . | . |  |
| **LncRNA number** | **Position** | **Strand** | **Control (FPKM)** | **IL1b (FPKM)** | **Absolute Change (FPKM)** | **Log2 (fold Change)** | **p-value** | **q-value** | **LncRNA Class** | **GenCode Number** | **Gencode Name** | **Human Body Map Number** | **Human Body Map Name** | **Map Name** |
| XLOC_005601 | chr1:100724075-100731683 | - | 1.80544 | 0.653495 | -1.151945 | -1.4661 | 5.00E-05 | 0.000794074 | LincRNA | ENSG00000224616.1 | RP11-305E17.6 | XLOC_000939 | linc-DBT-3 |  |
| XLOC_004137 | chr1:16793578-16805433 | - | 1.97739 | 0.713471 | -1.263919 | -1.47067 | 0.00035 | 0.00384313 | Pseudogene | ENSG00000080947.10 | CROCCP3 | . | . |  |
| XLOC_061064 | chr4:184413344-184425626 | - | 2.26695 | 0.928806 | -1.338144 | -1.28731 | 0.0002 | 0.00243059 | LincRNA | ENSG00000232648.3 | RP11-367N14.2 | . | . |  |
| XLOC_082895 | chr9:97440530-97444298 | + | 1.77993 | 0.250654 | -1.529276 | -2.82805 | 5.00E-05 | 0.000794074 | LincRNA | . | . | . | . |  |
| XLOC_061490 | chr5:27475700-27486171 | + | 2.81016 | 0.843 | -1.96716 | -1.73705 | 0.00155 | 0.0125952 | LincRNA | ENSG00000250337.1 | LINC01021 | XLOC_004323 | linc-CDH6-5 |  |
| XLOC_047732 | chr21:17999826-18006264 | + | 2.8288 | 0.627205 | -2.201595 | -2.17318 | 5.00E-05 | 0.000794074 | LincRNA | ENSG00000228798.1 | AP000473.5 | . | . |  |
| XLOC_082902 | chr9:97853658-97885336 | + | 3.4768 | 1.17906 | -2.29774 | -1.56012 | 5.00E-05 | 0.000794074 | Antisense | ENSG00000229065.1 | RP11-80I15.4 | . | . |  |
| XLOC_058057 | chr4:144480637-144531015 | + | 2.65057 | 0.318109 | -2.332461 | -3.05871 | 0.0029 | 0.0214523 | Pseudogene | ENSG00000236296.3 | GUSBP5 | . | . |  |
| XLOC_058819 | chr4:3952902-3957196 | - | 2.8034 | 0 | -2.8034 | #NAME? | 0.00025 | 0.00293431 | Pseudogene | ENSG00000251669.1 | FAM86EP | . | . |  |
| XLOC_046446 | chr20:1033422-1039924 | - | 3.0714 | 0 | -3.0714 | #NAME? | 0.00035 | 0.00384313 | LincRNA | . | . | . | . |  |
| XLOC_021107 | chr13:42916696-42958604 | + | 3.30001 | 0 | -3.30001 | #NAME? | 5.00E-05 | 0.000794074 | Pseudogene | ENSG00000233259.3 | FABP3P2 | . | . |  |
| XLOC_020092 | chr12:72065447-72079801 | - | 4.54157 | 0 | -4.54157 | #NAME? | 0.0002 | 0.00243059 | Antisense | . | . | . | . |  |
| XLOC_088851 | chrX:77785222-77914960 | - | 7.68217 | 2.00721 | -5.67496 | -1.93632 | 0.0003 | 0.00338689 | LincRNA | . | . | . | . |  |
| XLOC_067606 | chr6:52529231-52534103 | + | 10.5178 | 3.78112 | -6.73668 | -1.47594 | 0.0001 | 0.00141323 | Pseudogene | ENSG00000216775.2 | RP1-152L7.5 | XLOC_005323 | linc-TMEM14A-1 |  |
| XLOC_088688 | chrX:70293193-70315602 | - | 7.08662 | 0 | -7.08662 | #NAME? | 0.0021 | 0.0162261 | LincRNA | . | . | . | . |  |
| XLOC_078180 | chr8:17658850-17679894 | + | 10.8838 | 1.93975 | -8.94405 | -2.48824 | 5.00E-05 | 0.000794074 | LincRNA | ENSG00000253944.1 | RP11-156K13.1 | XLOC_006726 | linc-PCM1 |  |
