# Supplementary material for: Long Intergenic Noncoding RNAs Mediate the Human Chondrocyte Inflammatory Response and Are Differentially Expressed in Osteoarthritis Cartilage
Source: Arthritis Rheumatol. 2016 Mar 28;68(4):845–56. doi: 10.1002/art.39520 (PMC4950001; doi:10.1002/art.39520)
Supplement: Supplementary file 8 — Supplementary Table 5. Human Bio‐plex 17‐plex data following 4h IL‐1β stimulation of human TC28 chondrocytes transfected with LNA GAPmeRs. Fold change is shown for those analytes which were significantly different (P < 0.05) relative to the non‐targeting control LNA. Data which were not statistically significant are shown as ‘NS’ whilst analytes which were not detectable are shown as ‘n.d’. IL‐1β (1ng/ml) was used as the cell stimulant and thus was discounted from these data. [file ART-68-845-s008.docx]

**Supplementary Table 5.**

Human Bio-plex 17-plex data following 4h IL-1β stimulation of human TC28 chondrocytes transfected with LNA GAPmeRs. Fold change is shown for those analytes which were significantly different (P<0.05) relative to the non-targeting control LNA. Data which were not statistically significant are shown as ‘*NS*’ whilst analytes which were not detectable are shown as ‘n.d’. IL-1β (1ng/ml) was used as the cell stimulant and thus was discounted from these data.

|  | Fold change | |
| --- | --- | --- |
| Analyte | **CILinc01** | **CILinc02** |
| IL-1b | - | - |
| IL-2 | *NS* | *NS* |
| IL-4 | n.d | n.d |
| IL-5 | *NS* | NS |
| IL-6 | *+2.3* | *+1.7* |
| IL-7 | n.d | n.d |
| IL-8 | +2.3 | *NS* |
| IL-10 | *NS* | *NS* |
| IL-12 (p70) | *NS* | *NS* |
| IL-13 | n.d | n.d |
| IL-17 | n.d | n.d |
| G-CSF | +3.6 | *NS* |
| GM-CSF | *NS* | *NS* |
| IFNg | *NS* | *NS* |
| MCP-1 | *NS* | *NS* |
| MIP-1b | +2.2 | *NS* |
| TNFa | +1.5 | *NS* |
